# Design, synthesis and evaluation of arylpurine-based sinefungin mimetics as zika virus methyltransferase inhibitors

**DOI:** 10.1039/d5ra05362e

**Published:** 2025-10-07

**Authors:** Natalia del Río, Iván Arribas-Álvarez, José-María Orduña, Priscila Sutto-Ortiz, Johan Neyts, Suzanne Kaptein, Etienne Decroly, Eva-María Priego, María-Jesús Pérez-Pérez

**Affiliations:** a Instituto de Química Médica (IQM, CSIC) Juan de la Cierva 3 28006 Madrid Spain mjperez@iqm.csic.es empriego@iqm.csic.es; b Escuela de Doctorado, Universidad Autónoma de Madrid Spain; c Architecture et Fonction des Macromolécules Biologiques (AFMB), Aix-Marseille Univ., CNRS Faculté des Sciences Campus Luminy Marseille France; d KU Leuven, Department of Microbiology, Immunology and Transplantation, Rega Institute for Medical Research, Virology, Antiviral Drug & Vaccine Research Group Leuven Belgium

## Abstract

Arylpurine derivatives were designed and synthesized to mimic sinefungin by targeting the SAM/SAH binding site of zika virus (ZIKV) methyltransferase (MTase). These compounds incorporate adenine or 6-methyl-7-deazapurine bases, while the ribose of sinefungin has been replaced by an aniline, linked to its amino acid chain *via* a CO or a CH_2_ unit. Compounds 18, 29 and 31 inhibited ZIKV 2′-*O*-MTase activity. Docking studies showed that compounds 18 and 29 interact with both the purine and amino acid binding sites, effectively mimicking sinefungin. In contrast, compound 31 has its amino acid chain positioned above the ribose binding site. Notably, compound 18 exhibited modest antiviral activity against ZIKV.

## Abstract 2

Two new series of arylpurines mimicking sinefungin have been synthesized and evaluated as ZIKV methyltransferase inhibitors.

## Graphical abstract

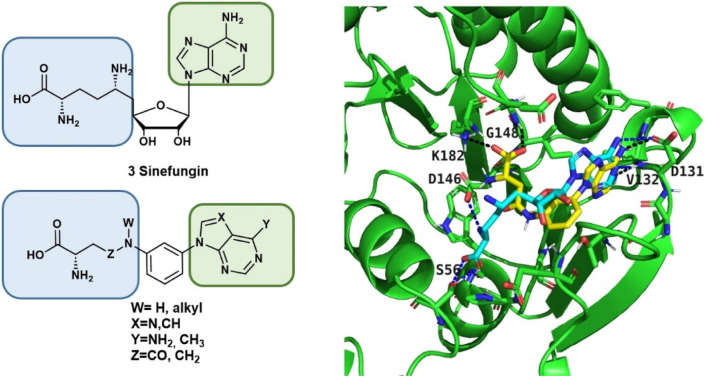

## 1 Introduction

**Fig. 1:**
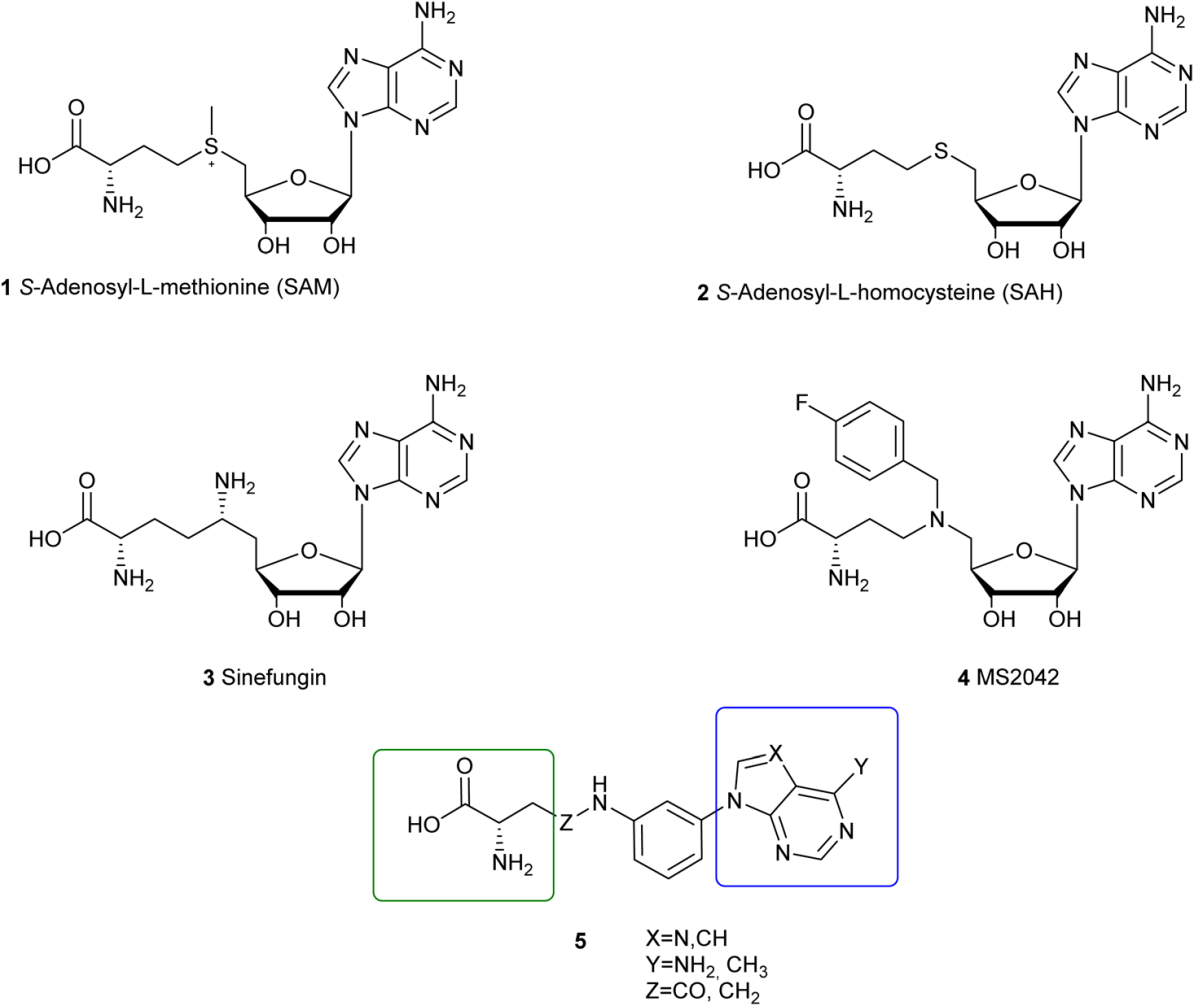
Chemical structures of SAM, SAH, sinefungin, MS2042 and the general formula of the newly synthesized arylpurines.

*S*-Adenosyl-l-methionine (SAM) (1, Fig. 1) is the universal substrate for enzymes involved in methyltransferase activities, being converted to *S*-adenosyl-l-homocysteine (SAH) (2, Fig. 1), which acts as an endogenous competitive inhibitor. Methyltransferases (MTases) are considered as key players in epigenetics, regulating gene expression through covalent modification of histones or nucleic acids (DNA or RNA).^1–3^ Among the numerous MTases that are being considered as relevant targets for therapeutic intervention, viral MTases of single-stranded positive sense (+ss) RNA viruses constitute a particularly appealing group of enzymes,^4,5^ generating the RNA cap structure present at the 5′ end of the viral mRNA. This process plays a key role in viral replication including efficient recognition for translation, protection from degradation by exonucleases or involvement in the immune response.^6–8^ Recent reports have largely focused on SARS-CoV-2 methyltransferases, particularly nsp14, reflecting the urgent need for targeted therapeutics during the COVID-19 pandemic.^9–11^

In the *Orthoflavivirus* genus, one of the most relevant genera of (re)emerging (+ss) RNA viruses, the MTase activity is located at the N-terminal of the viral NS5 protein. This enzyme exhibits bifunctionality by catalyzing both N7 and 2′-*O* methylations of the RNA cap structure, sequentially modifying it into a cap1 structure. In both methylation steps, *S*-adenosylmethionine (SAM) serves as the methyl group donor.^12^ Interestingly, key mutations in the *Orthoflavivirus* methyltransferase domain reduce or even abolish viral replication,^12^ thus supporting the interest of this target in antiviral drug development. Moreover, the similarities in sequence and structure of this domain in different *Orthoflavivirus*, particularly zika and dengue viruses (ZIKV and DENV, respectively) suggest that broad-spectrum anti-orthoflaviviral compounds can be developed by designing methyltransferases inhibitors.^13–15^ Advances in the search for ZIKV and/or DENV methyltransferase inhibitors have been recently reviewed.^14,16,17^

The structure of the ZIKV methyltransferase has been solved with SAH (PDB: 5TMH)^18^ or SAM (PDB ID: 5KQR).^19^ Sinefungin (3, Fig. 1), a naturally occurring nucleoside isolated from *Streptomyces* cultures, has been identified as a potent ZIKV and DENV MTase inhibitor,^13^ and its complex with ZIKV methyltransferase has also been reported^20^ (PDB ID: 5MRK). However, the poor cellular permeability of sinefungin may account for its lack of antiviral activity in cell culture.^21^ In these ligand–protein complexes, residues that are involved in adenine binding of nucleosides 1–3 include T104–E111 and D131–V132, while residues S56, K61, R84–W98, I145–D146, K182, and E218 make contact with the amino acid side chain at the 5′ position of the nucleosides.^17^ Interestingly, the SAM analogue MS2042 (4, Fig. 1) has also been co-crystalized with ZIKV methyltransferase (PDB: 5ULP),^22^ where the 4-fluorobenzyl group exploits a cavity oriented towards the RNA tunnel.

Based on our previous results in the synthesis of aryl derivatives of purines and related heterocycles,^23–25^ we conceptualized the synthesis of compounds based on general formula 5 (Fig. 1) that could bind the SAH/SAM binding pocket of the ZIKV MTase. In this series, the ribose ring of SAH or sinefungin is replaced by an aniline that incorporate the amino acid chain (in a green box in general formula 5) through a linker (Z = CH_2_ or CO). By replacing the ribose by an aryl ring, we aim to reduce the hydrophilicity of the compounds and thus increase their permeability in cell cultures in order to obtain antiviral activity. As purines (blue box in general formula 5), we selected adenine (X = N; Y = NH_2_) as in nucleosides 1–4, or 6-methyl-7-deazapurine (X = CH; Y = CH_3_). This deaza analogue has shown significant promise, as its substitution for adenine in (carba)nucleosides has yielded highly potent inhibitors of protein arginine methyltransferase 5 (PRMT5), with several candidates advancing to clinical trials.^26–28^ Cocrystal structures of PRMT5 with these inhibitors (*i.e.*, PF-06939999 in PDB: 7MX7)^27^ have revealed that 6-methyl-7-deazapurine perfectly occupies the adenine pocket at the SAM/SAH binding site. Thus, we here describe the synthesis of compounds of general structure 5, their enzymatic inhibition of ZIKV and DENV MTases, docking studies at the SAM/SAH binding site of ZIKV MTase, and their effectiveness in inhibiting ZIKV replication.

## 2 Results and discussion

### 2.1 Chemistry

**Scheme 1:**
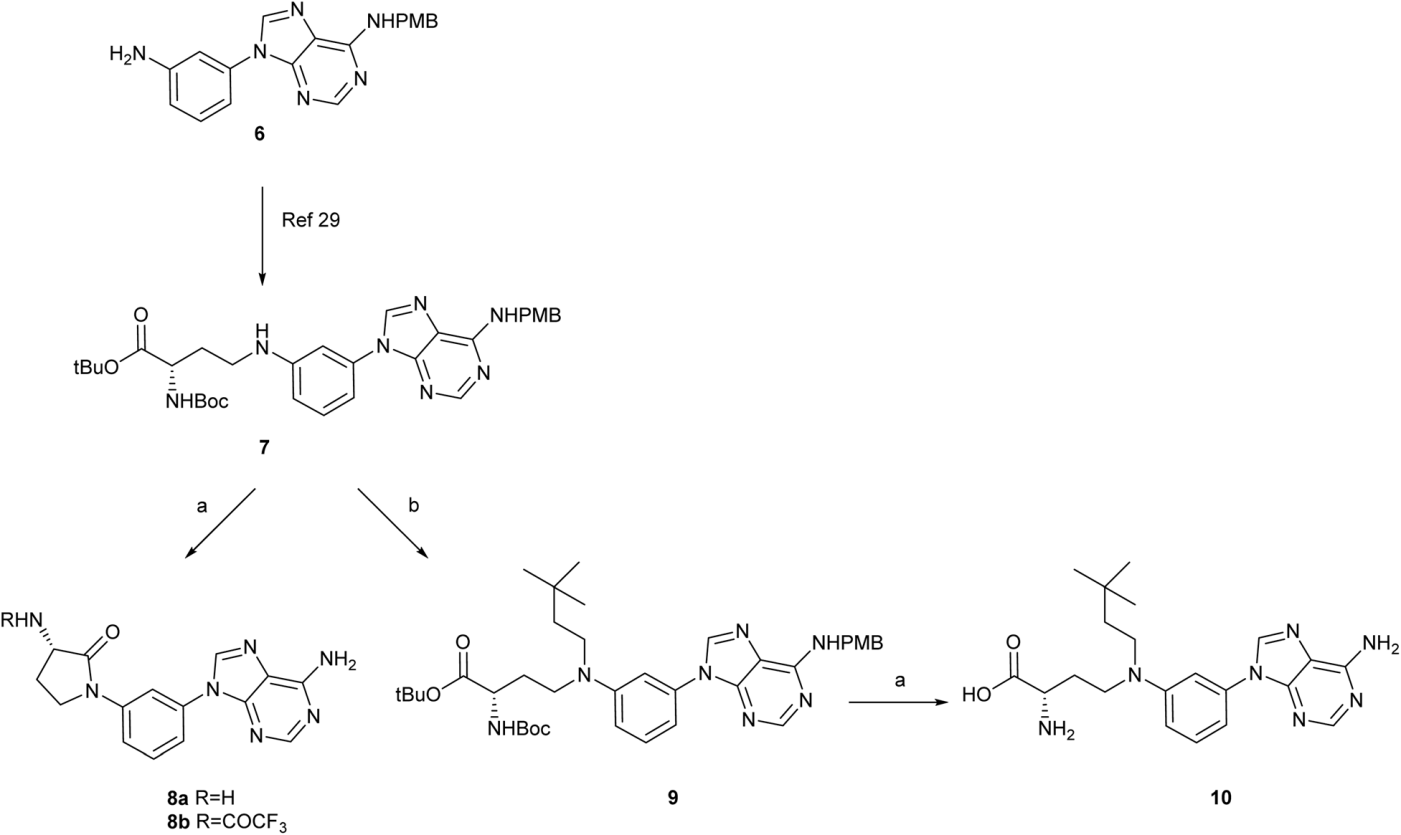
Reagents and conditions: (a) neat TFA, 70 °C, 16 h, for 10: 76%; (b) 3,3-dimethylbutanal, H-cube, 10% Pd/C (CatCart™, 30 mm), 150 °C, 60 Bar, 0.5 mL min^−1^, MeOH, 6 h, 54%.

Aniline derivative 6 (ref. 29) (Scheme 1), with a *p*-methoxybenzyl (PMB) protecting group at position 6 of the adenine, was employed as a suitable synthon for the synthesis of the adenine derivatives (X = N; Y = NH_2_ in the general formula 5). Compound 6 was synthesized starting from 4,6-dichloropyrimidin-5-amine and 3-nitroaniline in 4 steps, as previously described.^29^ Reaction of 6 with *tert*-butyl (*S*)-2-((*tert*-butoxycarbonyl)amino)-4-oxobutanoate afforded compound 7 (ref. 29) in 45% yield.

To remove in a single step the Boc and *tert*-butyl groups at the amino acid side chain as well as the *p*-methoxybenzyl group at position 6 of the adenine, compound 7 was treated with TFA at 70 °C overnight, as applied in previous examples.^29,30^ Analysis of the reaction mixture by HPLC/MS indicated a major peak with a *m*/*z* = 310, *i.e.* 18 units less than expected for the fully deprotected compound, that was tentatively assigned as the cyclized product 8a. This was accompanied by a second peak with *m*/*z* = 406, that might correspond to the trifluoroacetyl derivative 8b (see SI Fig. S1 for HPLC/MS chromatograms). A follow-up of the reaction by HPLC/MS indicated that after 90 min, a major compound with *m*/*z* = 430 is formed that might correspond to the cyclization of the carboxylic acid generated after *tert*-butyl deprotection with the amino of the aniline leading to a 3-aminopyrrolidine-2-one, while the *p*-methoxybenzyl group at 6 is still present (see SI Fig. S1). A previous study involving the synthesis of aspartamides from aniline derivatives^31^ described that attempts to deprotect the aspartic acid ester, using basic hydrolysis or elevated temperatures, resulted in intramolecular cyclization, yielding 3-aminopyrrolidine-2,5-diones.

Based on these results, it was clear that intramolecular cyclization took place prior to removal of the PMB group, that is only released after long reaction times and heating. Thus, we reasoned that introduction of a second substituent at the aniline could prevent the cyclization reaction during deprotection. Moreover, the X-ray structure of the double substituted nucleoside MS2042 (4) with ZIKV MTase,^22^ evidenced the existence of a hydrophobic cavity that could lodge a second substituent at the aniline (see the Computational studies section). Thus, reaction of 7 with 3,3-dimethylbutanal in the H-cube at 150 °C and 60 Bar under recirculation conditions, as set up by us for similar analogues,^29^ afforded the double-substituted compound 9 in 54% yield (Scheme 1). Treatment of 9 with TFA at 70 °C afforded 10 as the main product that was isolated by reverse phase flash chromatography in 76% yield.

**Scheme 2:**
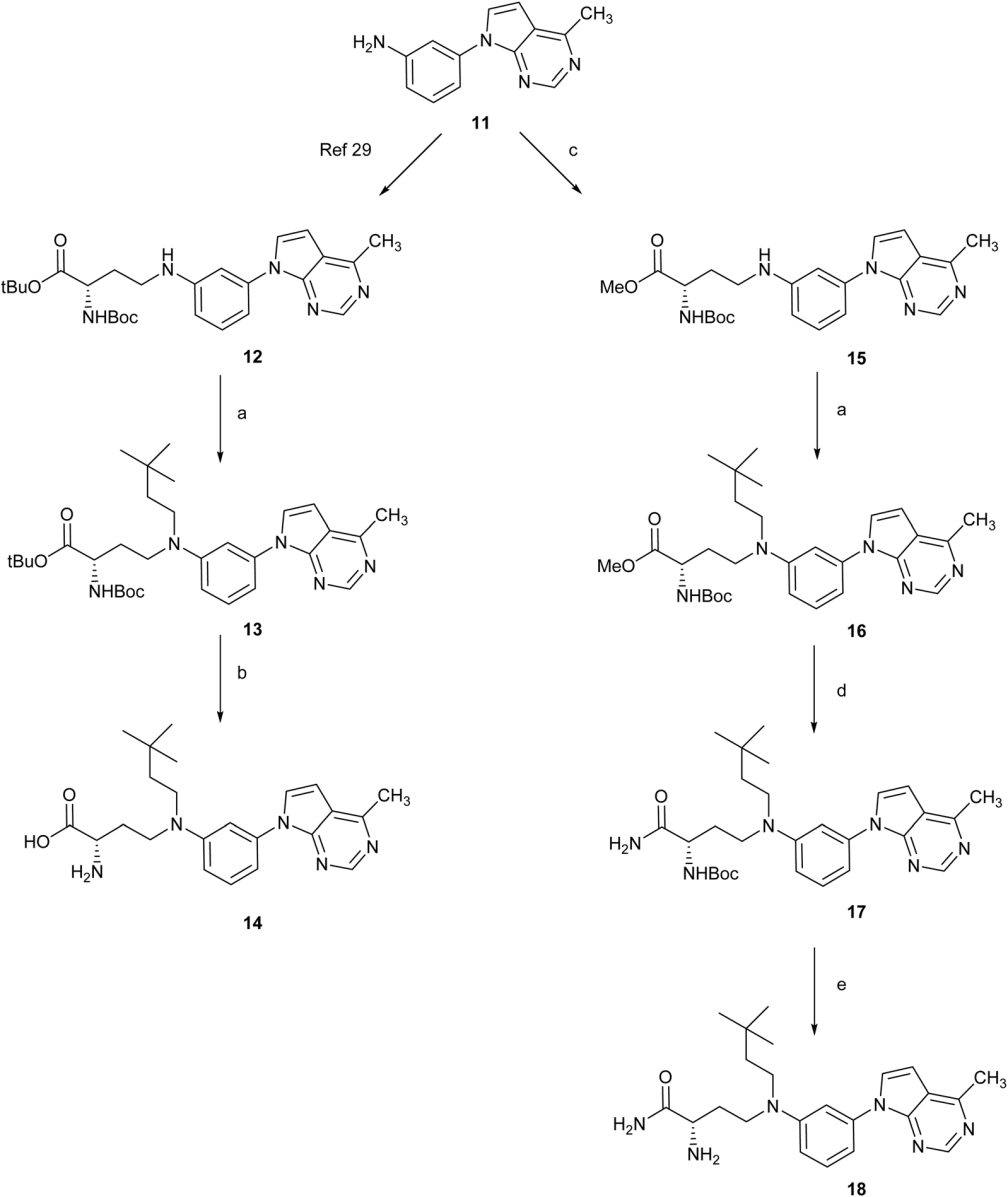
Reagents and conditions: (a) 3,3-dimethylbutanal, H-cube, 10% Pd/C (CatCart™, 30 mm), 150 °C, 60 Bar, 0.5 mL min^−1^, MeOH 0.05 M, recirculation, 4 h, for 13: 25%, for 16: 50%; (b) TFA, DCM, 25 °C, 2 h, quantitative; (c) methyl (*S*)-2-((*tert*-butoxycarbonyl)amino)-4-oxobutanoate, 10% Pd/C (CatCart™, 30 mm), 65 °C, 40 Bar, 0.5 mL min^−1^, MeOH 0.05 M, recirculation, 2 h, 41%; (d) NH_3_/MeOH 7M, 25 °C, 4 days, 33%; (e) HCl/dioxane 4N, 25 °C, 1 h, 72%.

Next, we addressed a similar strategy for the 7-deaza-6-methylpurine derivatives (Scheme 2). The aniline 11 (ref. 29) reacted with *tert*-butyl (*S*)-2-((*tert*-butoxycarbonyl)amino)-4-oxobutanoate as described^29^ to provide 12 in a 73% yield. Then, reaction of 12 with 3,3-dimethylbutanal afforded 13 in 25% yield. Treatment of 13 with TFA in dichloromethane led to its quantitative conversion into compound 14. In order to reduce the ionic character of the final compounds, we considered an analogue of 14 with a distal α-amino amide. To this end, the aniline 11 reacted with the aldehyde of the amino acid now protected as a methyl ester providing compound 15 in 41% yield. This compound was subjected to a second reductive amination reaction with 3,3-dimethylbutanal to afford 16 (50% yield). Treatment of this methyl ester with NH_3_/MeOH for 4 days led to the amide 17 whose treatment with HCl 4N in dioxane provided compound 18 in 72% yield.

**Scheme 3:**
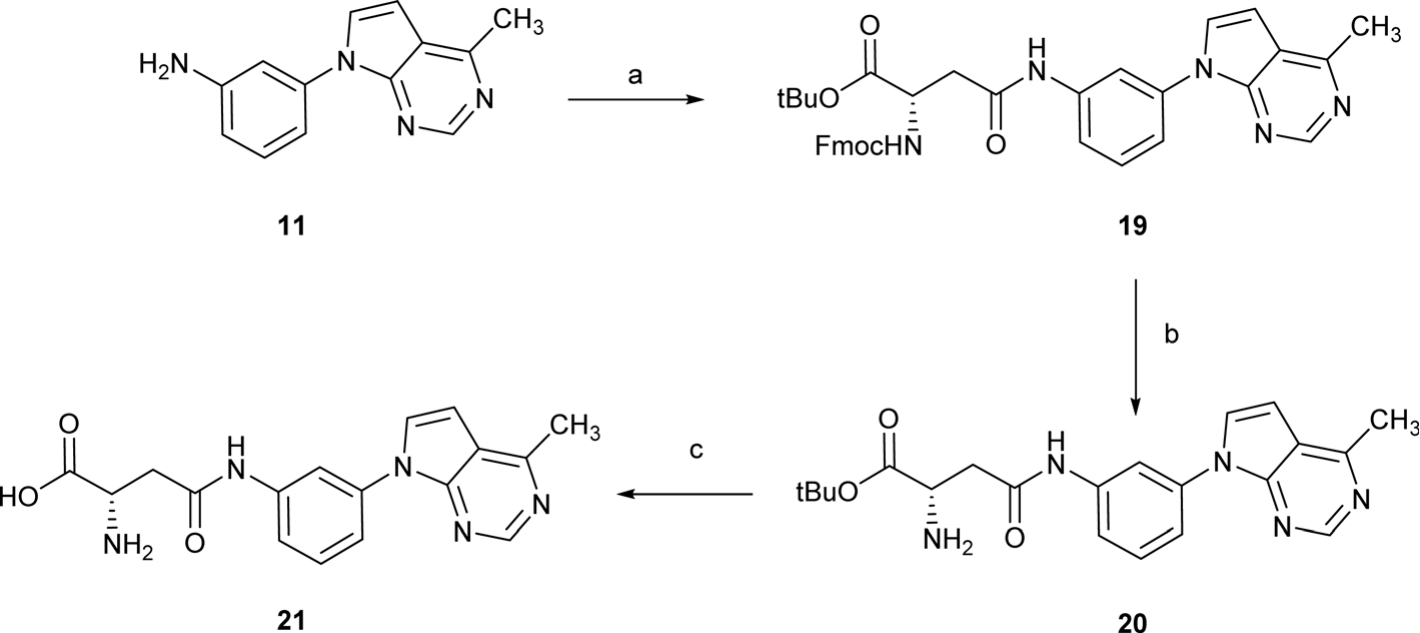
Reagents and conditions: (a) Fmoc-Asp-OtBu, HATU, DIPEA, DMF, 40 °C, 2.5 h, 50%; (b) piperidine, DCM, 25 °C, 2 h, 70%; (c) TFA, DCM, 25 °C, 16 h, quantitative.

On the other hand, reaction of the aniline 11 with Fmoc-Asp-OtBu in the presence of HATU and DIPEA in DMF at 40 °C afforded the amide 19 in 50% yield (Scheme 3). Removal of the Fmoc group by treatment with piperidine afforded compound 20 (70% yield). Further reaction of 20 with TFA in DCM led to its quantitative conversion into the fully deprotect compound 21.

**Scheme 4:**
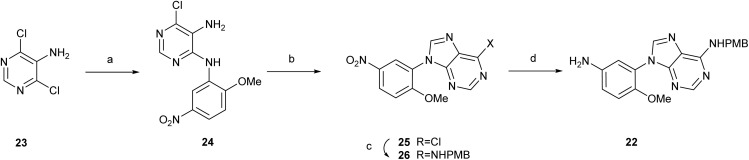
Reagents and conditions: (a) 2-methoxy-5-nitroaniline, MWI, isobutanol, 150 °C, 10 min, 46%; (b) trimethylorthoformate, HCl, 120 °C, 1 h, 83%; (c) *p*-methoxybenzylamine, DIPEA, isopropanol, MWI, 100 °C, 30 min, 83%; (d) SnCl_2_, AcOEt, EtOH, 80 °C, 2 h, 60%.

**Fig. 2:**
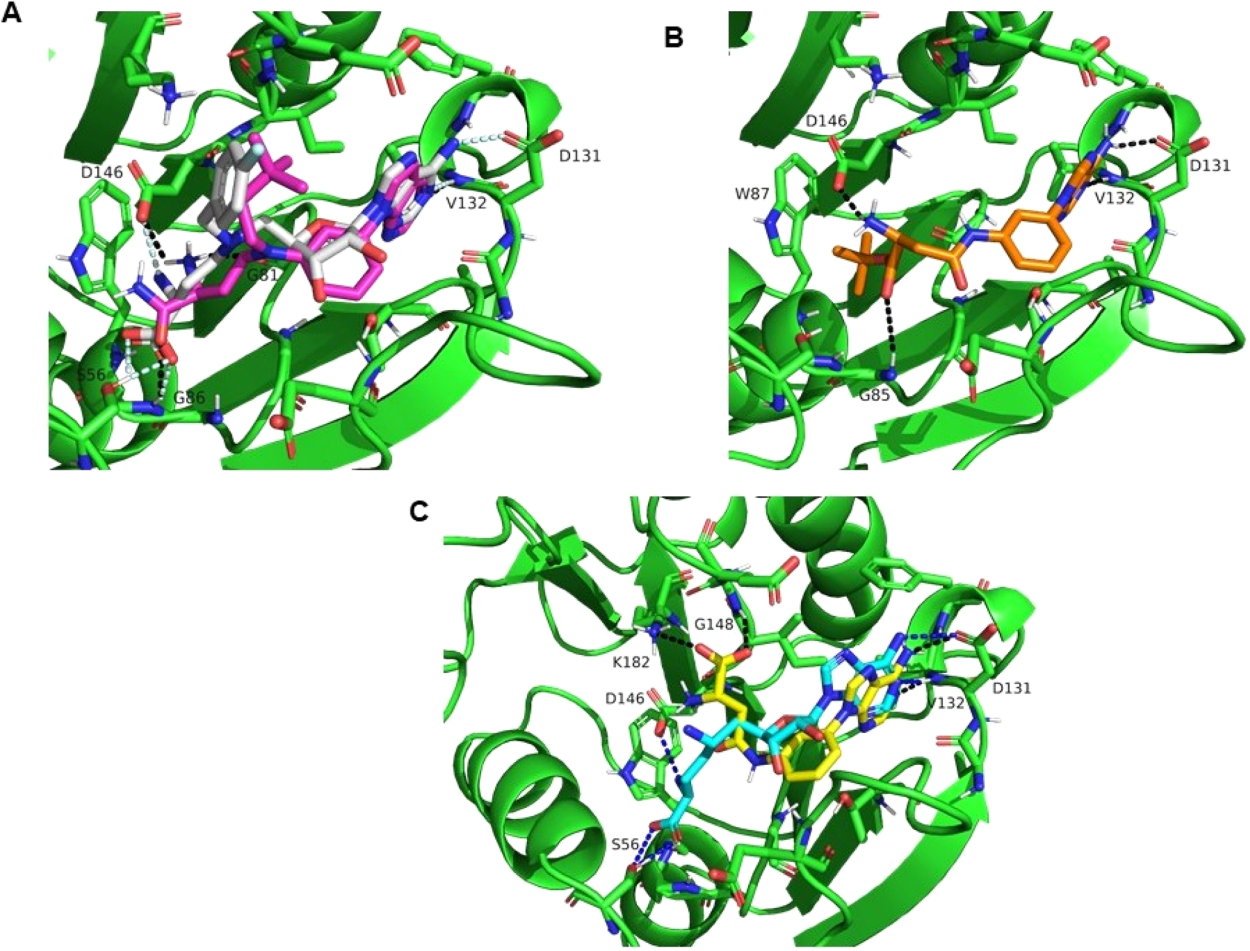
Docking poses of compounds 18, 29 and 31 in ZIKV MTase. (A) Binding mode of 18 (pink sticks) and its superimposition with MS2042 (PDB: 5ULP). (B) Binding mode of 29 (orange sticks). (C) Binding mode of 31 (yellow sticks) and its superimposition with sinefungin (cyan sticks, PDB: 5MRK). In all cases ZIKV MTase is shown as green cartoon, selected residues interacting with the compounds are shown in sticks and labelled, hydrogens bonds are shown as dashes in black for 18, 29 and 31, in pale cyan for MS2042 and in blue for sinefungin.

A similar synthetic strategy was applied to the adenine derivatives, although in this case two different anilines were used as starting materials: compound 6 and an analogous aniline bearing a 2-methoxy substituent (22) (Scheme 4). Docking studies indicate that the binding pocket provides sufficient room at this position (see Fig. 2C in the Computation studies section and Fig. S2 at the SI), and suggest that a methoxy group may participate in hydrophobic and/or hydrogen bond-mediated interactions with ZIKV MTase. Thus, reaction of 4,6-dichloropyrimidin-5-amine (23) with 2-methoxy-5-nitroaniline under MWI at 150 °C for 10 minutes provided the pyrimidine derivative 24 in 46% yield. This poorer yield compared to previously described examples with other anilines^23,29^ is probably due to the presence of the OMe group at position 2. Treatment of 24 with trimethylorthoformate at 120 °C under MWI for 1 hour afforded the 6-chloropurine 25. Further reaction of 25 with *p*-methoxylbenzylamine under MWI at 100 °C for 1 hour provided compound 26. Treatment of 26 with SnCl_2_ in a EtOH/EtOAc mixture under reflux for 2 h led to the aniline derivative 22 in 60% yield.

**Scheme 5:**
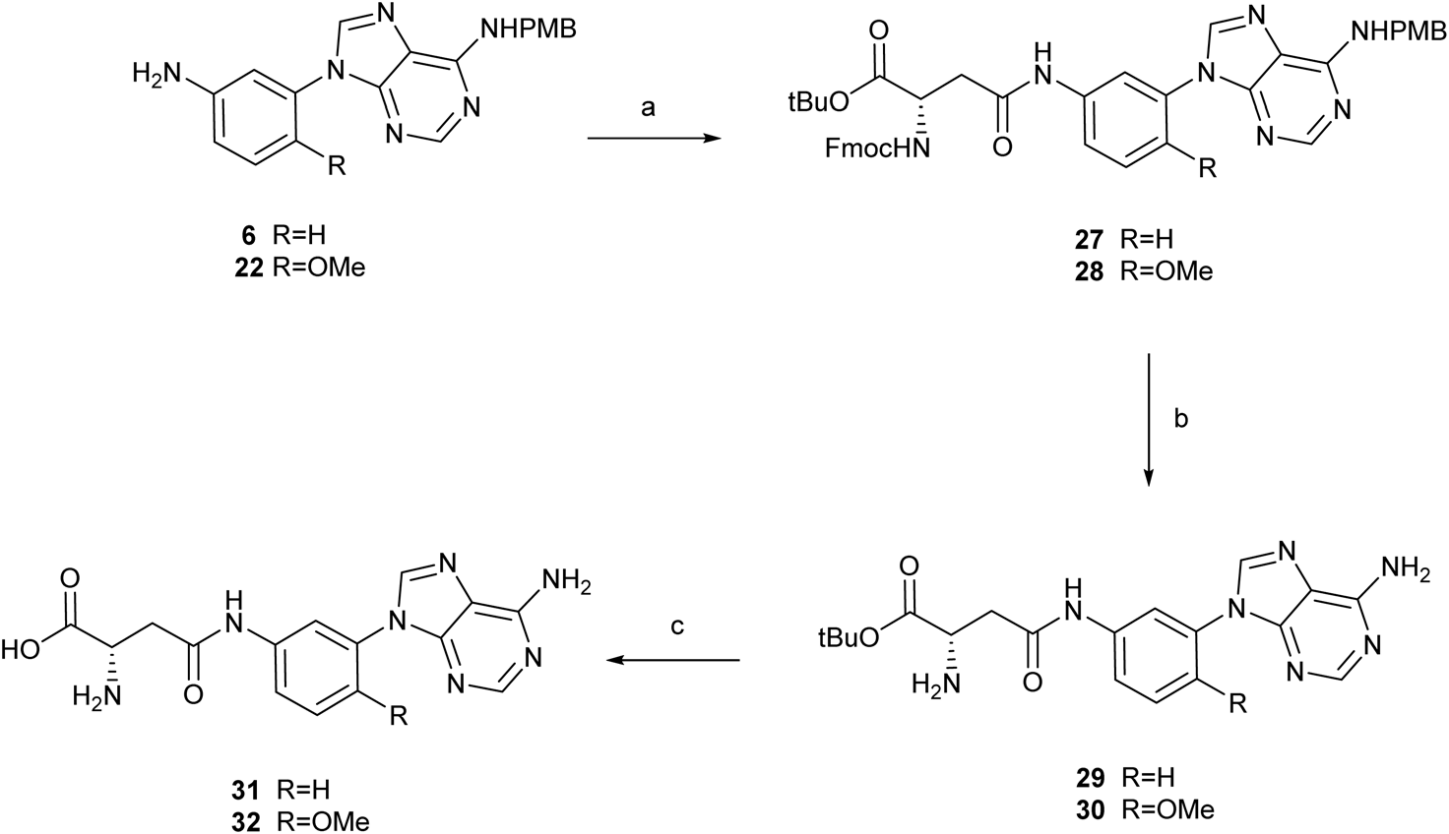
Reagents and conditions: (a) Fmoc-Asp-OtBu, HATU, DIPEA, DMF, 40 °C, 2.5 h, for 27: 65%; for 28: 97%; (b) (i) (NH_4_)_2_S_2_O_8_, PBS/CH_3_CN (1 : 1), MWI, 80 °C, 45 min; (ii) piperidine, DCM, 25 °C, 2 h; for 29: 25% (2 steps); for 30: 23% (2 steps); (c) TFA, DCM, 25 °C, 6–48 h: for 31: 73%; for 32: 62%.

The anilines 6 and 22 reacted with Fmoc-Asp-OtBu, as described for 19, to afford the amides 27 and 28 in 55% and 85% yields, respectively (Scheme 5).

Then, compound 28 was treated with TFA at 70 °C to remove the *tert*-butyl ester and the *p*-methoxybenzyl group. Analysis of the reaction by HPLC/MS after 1 h indicated a mixture of two compounds with *m*/*z* = 695 and *m*/*z* = 575, that might correspond to cyclized products with and without the *p*-methoxybenzyl group at position 6 (see Fig. S3 at the SI). This indicated that for the acylated anilines intramolecular cyclization is also highly favored. In exploring alternative methods for removing the PMB group, we came across a report by Sako *et al.*, which described the oxidative debenzylation of 6-*N*-benzyladenosines under neutral pH conditions.^32^ Since our compounds are more lipophilic than the nucleosides described by Sato, we changed the PBS : CH_3_CN ratio from 2 : 1, as described, to 1 : 1 to favor the solubility of our compounds. Additionally, we used microwave irradiation to shorten the reaction times. Thus, treatment of 27 with (NH_4_)_2_S_2_O in PBS : CH_3_CN (1 : 1) at 80 °C for 45 min under MW irradiation led to the removal of the *p*-methoxybenzyl group, as detected by HPLC/MS. Further treatment of the reaction product with piperidine afforded 29 (25% yield over the 2 steps). A similar reaction sequence applied to 28 led to compound 30 (21% yield). Modification of the deprotection order, specifically, performing Fmoc deprotection prior to treatment with (NH_4_)_2_S_2_O, resulted in lower yields, underscoring the importance of the reaction sequence. Finally, treatment of 29 and 30 with TFA in DCM at rt afforded compounds 31 and 32 in 73% and 62% yields, respectively. To rule out the possibility of spontaneous cyclization following removal of both protecting groups from the amino acid, compounds 21, 31 and 32 were dissolved in a PBS : DMSO (95 : 5) mixture. HPLC analysis of the solutions over a 24-hour period revealed no additional peaks, indicating that the compounds remained intact. These results confirm that no spontaneous cyclization occurred under the tested conditions.

### 2.2 Evaluation of the inhibitory effect on the 2′-*O*-MTase activities of ZIKV and DENV3

The synthesized arylpurines were evaluated for their inhibitory activity against the 2′-*O*-MTase activity of ZIKV and DENV3 MTases by quantification of [^3^H]-methyl transfer from the [^3^H]-SAM on short synthetic capped RNA (GpppAC_4_), by using a filter binding assay (FBA).^13,33^ The universal methyltransferase inhibitor sinefungin was included as a positive control (Table 1), whose IC_50_ value against ZIKV MTase has already been reported using a similar assay.^13^ We assessed MTase inhibition by testing the synthesized compounds at a concentration of 50 μM.

**Table 1:** Percentage of inhibition of ZIKV and DENV3 2′-*O*-MTase activities by selected compounds at 50 μM

Compound	Percentage of inhibition of ZIKV MTase	Percentage of inhibition of DENV3 MTase
10	0	0
14	0	0
17	11.1	7.2
18	10.8	10.1
20	0	0
21	0	0
29	19.2	26.3
30	0	0
31	16.7	14.8
32	0	0
Sinefungin	97.9	96.8

Compounds 10, 14, 20, 21, 30 and 32 had no effect on the 2′-*O*-MTase activity of ZIKV and DENV3. Compounds 17 and 18, with a 7-deaza-6-methylpurine as the base and a double substitution in the aniline, showed a modest inhibition of both enzymes. Among the compounds with a CO linker between the aniline and the amino acid substituent, only compounds 29 and 31 with an adenine core showed some inhibition against both enzymes. In addition, the presence of a *tert*-butyl ester in 29 might indicate that a free carboxylic acid is not strictly required for inhibition.

### 2.3 Computational studies

In order to shed light on the molecular details of the interaction of the new derivatives within the SAM/SAH binding pocket in ZIKV MTase, docking studies using PDB ID: 5ULP as template were carried out with compounds 18, 29 and 31, that were found to inhibit the ZIKV MTase, as shown in Table 1.

The best docking pose of compound 18 (Fig. 2A) provides a good overlay with the conformation of MS2042 (ref. 22) in complex with the ZIKV MTase (PDB: 5ULP). Thus, the 3,3-dimethylbutyl substituent is correctly oriented towards the hydrophobic cavity explored by the 4-fluorophenyl group of MS2042. Moreover, the 6-methyl-7-deazapurine ring interacted with V132 at the adenine binding site of MS2042. As for the amino acid side chain of compound 18, the CO of the primary amide could form a hydrogen bond with the NH of G86, while the terminal amino group could interact with the side chain of D146.

Regarding adenine derivative 29 (Fig. 2B), the compound nicely fits at the SAM/SAH binding site, with the adenine base interacting with D131 and V132, while the amino and carbonyl groups of the amino acid chain of 29 are at hydrogen-bond distance of the carboxylic acid side chain of D146 and the amino group of the backbone of G85, respectively. As a result of these interactions, the *tert*-butoxy substituent is oriented towards the cavity delimited by the side chain of W87.

With respect to the docking pose of compound 31, the binding of the adenine base is very similar to that of sinefungin (PDB ID: 5MRK)^20^ (Fig. 2C), *i.e.* it is stabilized by two polar interactions with D131 and V132. However, the amino acid chain in 31 is directed towards the upper region of the binding pocket, so that the terminal carboxylic acid is situated at hydrogen-bond distance of the side chain of K182 and the backbone NH of G148, while the amino group could interact with the backbone CO of D146. The stability of this binding pose was checked by molecular dynamics simulations by monitoring the RMSD values and the evolution of the interactions (see Fig. S4 in the SI), showing that compound 31 remained stable in the SAH binding pocket, with RMSD values between 0.86 and 2.8 Å, while the interactions with V132 and D146 were kept along the simulation. Moreover, we carried out a binding free energy estimation along the MD simulation for compound 31 using the software MM-ISMSA.^34^ The estimated Δ*G* for the complex MTase-31 is −31.09 ± 16.34 kcal mol^−1^. Altogether it can be concluded that the proposed binding mode of the adenine derivative 31 within ZIKV MTase is theoretically possible, although other factors, *i.e.* desolvation phenomena and/or short time of residence, among others, may account for the weak *in vitro* inhibition value.

### 2.4 Antiviral evaluation against ZIKV in a cell-based assay

These compounds were next evaluated for their antiviral effect on ZIKV replication in Vero E6 and SH-SY5Y cells. 7-Deaza-2′-*C*-methyladenosine (7DMA), a viral polymerase inhibitor,^35^ was included as a positive control. The results obtained are shown in Table 2.

**Table 2:** Antiviral effect of selected compounds against ZIKV in VeroE6 and SH-SY5Y cells

Compound	VeroE6		SH-SY5Y	
	EC_50_^a^ (μM)	CC_50_^b^ (μM)	EC_50_^a^ (μM)	CC_50_^a^ (μM)
10	>100	>100	>100	>100
14	>100	>100	>100	>100
17	>100	22.8 ± 0.5	>100	7.0
18	27.2 ± 0.2	66.2 ± 3.9	>100	61.0
20	>100	>33	>100	>100
21	>100	>100	>100	>100
29	>100	>100	>100	>100
30	>100	>100	>100	>100
31	>100	>100	>100	>100
32	>100	>100	>100	>100
7DMA	9.0 ± 0.2	>200	33.7 ± 5.1	>50

a EC_50_ or 50% effective concentration indicates the concentration that protects 50% of the cells from the cytopathic effect induced by the virus.

b CC_50_ or 50% cytotoxic concentration indicates the concentration that shows a cytotoxic/cytostatic effect in 50% of the non-infected cells.

Although most compounds were non-toxic to the host cells, they also did not exert an antiviral effect against ZIKV. In VeroE6 cells, compound 17 showed a CC_50_ value of 22.8 μM while compound 20 showed some toxicity at concentrations higher than 33 μM. Interestingly, compound 18 presented an EC_50_ value of 27 μM while its CC_50_ value is almost 3-fold higher. Notably, this compound was also found to inhibit the ZIKV MTase (Table 1).

Besides the standard assays in VeroE6 cells, the compounds were also tested in SH-SY5Y cells. These cells have been shown to be good representatives of immature neurons as they express immature neuronal markers.^36,37^ Thus, they function as a relevant neuronal cell-based model of ZIKV infection.^38^ However, the mild antiviral activity of compound 18 was not confirmed in the neuroblastoma SH-SY5Y cells, a cell line where the reference compound 7DMA is also less active (2–3 fold) than in Vero E6 cells.

## 3 Conclusions

The synthesis of arylpurine derivatives mimicking sinefungin was explored. To this end, two series of aniline derivatives have been synthesized, each containing either an adenine or a 6-methyl-7-deazapurine as the heterocyclic base. These compounds incorporate an amino acid-mimicking the substituent at the 5′-position of sinefungin. Unexpected intramolecular cyclization reactions affecting the amino acid chain occurred while removing the *p*-methoxybenzyl group at position 6 in the adenine series with neat TFA under heating conditions. Adaptation of previously described oxidative conditions ((NH_4_)_2_S_2_O in a mixture PBS : CH_3_CN) allowed removal of the *p*-methoxybenzyl group in the amide series, but no satisfactory results using this approach were obtained in the alkyl series. Alternatively, a second reductive amination was performed on the NH-alkylated compounds to generate bis-substituted analogues, effectively preventing cyclization during the subsequent deprotection steps.

The synthesized aryl purines were evaluated for their inhibitory activity against ZIKV and DENV3 2′-*O*-MTase. The adenine derivatives 29 and 31 at 50 μM showed around 20% of inhibition of both targets although no antiviral activity against ZIKV was observed in a cell-based assay. Alternatively, compound 18, with a slight reduction in enzymatic inhibition of the ZIKV MTase afforded antiviral activity in cell culture with an IC_50_ = 27.2 ± 0.2 μM in Vero E6 cells. It may be argued that the high polar nature of 29 and 31 could account for the lack of antiviral activity in cell culture while the less polar nature of 18 may facilitate cell entry and enzymatic inhibition.

Docking studies performed with compound 18 and 29 at the SAM/SAH binding site of the ZIKV MTase indicate a similar orientation of both the purine base and the amino acid chain as in MS2042. Moreover, in the case of 18, the *tert*-butyl group is lodged at the cavity where the 4-fluorobenzyl group of MS2042 is located. However, the pose obtained for compound 31 fixes the amino acid side chain towards a cavity located above the furanose ring of SAH. Hence, this cavity might be considered in the design of new analogues. Although docking studies confirmed that the synthesized arylpurines fit well within both the adenine-binding pocket and the amino acid sites of ZIKV MTase, experimental data revealed that these compounds exhibited only modest inhibition of the ZIKV and DENV MTases. These findings suggest that binding at those regions is insufficient for potent inhibition. To enhance efficacy, future inhibitor designs may need to focus on interactions with the hydroxyl groups at the 2′ and 3′ positions of the ribose moiety in molecules such as SAM/SAH or MS2042.

## 4 Experimental section

### 4.1 Chemistry

Melting points were measured on a M170 apparatus (Mettler Toledo, Columbus, Ohio, USA) apparatus and are uncorrected. The elemental analysis was performed with a CHN-*O*-RAPID instrument (Heraus, Hanau, Germany). The elemental compositions of the compounds agreed within ±0.4% of the calculated values.

^1^H and ^13^C NMR spectra were recorded on a Brucker AVANCE III HD-400 or a JEOL JNM-ECZ400R operating at 399 MHZ (^1^H) and 99 MHz (^13^C), respectively, and a VARIAN SYSTEM-500 operating at 499 MHz (^1^H) and 125 MHz (^13^C), respectively. Monodimensional ^1^H and ^13^C spectra were obtained using standard conditions. For compounds 18 and 29, 2D inverse proton detected heteronuclear one-bond shift correlation spectra were obtained using the Pulsed Field Gradient HSQC pulse sequence. Data were collected in a 1024 × 256 matrix with a spectral width of 5197 or 6009 Hz in the proton domain and 16 611 or 17 099 Hz in the carbon domain, and processed in a 2048 × 1024 matrix. The experiment was optimized for one bond heteronuclear coupling constant of 150 Hz. 2D inverse proton detected heteronuclear long range shift correlation spectra were obtained using the Pulsed Field Gradient HMBC pulse sequence. The HMBC experiment was acquired in a 2048 × 256 or 1024 × 128 matrix with a spectral width of 6009 or 5197 Hz in the proton domain and 25 150 or 22 123 Hz in the carbon domain, and processed in a 2048 × 1024 matrix. The experiment was optimized for long range coupling constants of 7 Hz.

Microwave reactions were performed using the Biotage Initiator 2.0 single-mode cavity instrument from Biotage (Uppsala). Experiments were carried out in sealed microwave process vials using the standard absorbance level (400 W maximum power). The temperature was measured with an IR sensor on the outside of the reaction vessel. Reductive amination reactions were performed with a standard H-Cube Pro™ flow reactor (ThalesNano Technology, Inc. Budapest, Hungary) equipped with a 30 mm cartridge loaded with 10% Pd/C.

Compounds were analysed by HPLC/MS with a e2695 LC (Waters, Milford, Massachusetts, USA), coupled to a Waters 2996 photodiode array detector and a Waters Micromass ZQ. The column used is a Waters SunFire C18 2.1 × 50 mm, 3.5 μm, and the mobile phases were acetonitrile and H_2_O, together with a constant 5% of H_2_O with 2% formic acid. For high-resolution mass spectrometry (HRMS), an Agilent 6520 accurate mass quadrupole time-of-flight (QTOF) platform coupled with LC/MS and equipped with an electrospray interface (ESI) working in the positive-ion (ESI+) and negative-ion (ESI−) modes was used.

The conversion of starting material to reaction products was followed by HPLC analysis performed in Agilent 1120 compact LC, column ACE 5 C18-300 (15 cm × 4.6 mm), UV detection was performed at *λ* = 254 nm, and the flow rate was 1 mL min^−1^, using as mobile phase A H_2_O and as mobile phase B CH_3_CN (both containing 0.05% TFA).

Analytical TLC was performed on silica gel 60 F254 (Merck, Dramstand, Germany)-precoated plates (0.2 mm). Spots were detected under UV light (254 nm) and/or charring with ninhydrin or phosphomolybdic acid.

Separations on silica gel were performed by preparative centrifugal circular thin-layer chromatography (CCTLC) on a Chromatotron (Kiesegel 60 PF254 gipshaltig (Merck)), with a layer thickness of 1 and 2 mm and a flow rate of 4 or 8 mL min^−1^, respectively, and by flash chromatography on a Biotage Selekt system with cartridges of silica gel Biotage Sfär Silica HC Duo 20 μm and Biotage Sfär C18 Duo 30 μm.

#### 4.1.1 *tert*-Butyl (*S*)-2-((*tert*-butoxycarbonyl)amino)-4-((3,3-dimethylbutyl)(3-(6-((4-methoxybenzyl)amino)-9*H*-purin-9-yl)phenyl)amino)butanoate (9)

A solution containing 7 (ref. 29) (600 mg, 0.99 mmol) and 3,3-dimethylbutanal (192 mg, 1.98 mmol) in 20 mL of MeOH was recirculated through a 30 mm CatCart™ in the H-cube at 60 Bar, 100% of hydrogen production, at 150 °C and a flow rate of 0.5 mL min^−1^. After 6 h of recirculation, volatiles were removed and the residue was purified by flash chromatography (gradient 0–50% ethyl acetate in hexane), obtaining 366 mg (54% yield) of 9 as a white solid. Mp: 91–93 °C. MS (ES, positive mode): *m*/*z* 688 (M + H). ^1^H NMR (400 MHz, CDCl_3_) *δ*: 0.97 (s, 9H, (CH_3_)_3_), 1.40 (s, 9H, OC(CH_3_)_3_), 1.46 (s, 9H, OC(CH_3_)_3_), 1.48–1.58 (m, 2H, CH̲_2_*t*Bu), 1.96 (m, 1H, CH_2_), 2.14 (m, 1H, CH_2_), 3.27–3.47 (m, 4H, CH_2_, NCH̲_2_CH_2_*t*Bu), 3.79 (s, 3H, OCH_3_), 4.14–4.28 (m, 1H, Hα), 4.83 (br s, 2H, NHCH̲_2_Ar), 5.55 (d, *J* = 7.9 Hz, 1H, NHBoc), 6.24 (t, *J* = 5.3 Hz, 1H, NHPMB), 6.65 (dd, *J* = 2.2, 8.5 Hz, 1H, Ar), 6.78 (d, *J* = 7.8 Hz, 1H, Ar), 6.87 (d, *J* = 8.5 Hz, 2H, PMB), 7.05 (s, 1H, Ar), 7.33 (dd, *J* = 4.4, 8.2 Hz, 3H, PMB, Ar), 7.99 (s, 1H, H_2_/H_8_), 8.51 (s, 1H, H_2_/H_8_). ^13^C NMR (100 MHz, CDCl_3_) *δ*: 28.1 (C(C̲H_3_)_3_), 28.4 (C(C̲H_3_)_3_), 29.5 (C(C̲H_3_)_3_), 29.9 (CH_2_), 30.0 (C̲(CH_3_)_3_), 39.9 (NCH_2_C̲H_2_*t*Bu), 44.2 (CH_2_NH), 47.4 (CH_2_), 47.5 (NC̲H_2_CH_2_*t*Bu), 52.6 (Cα), 55.4 (OCH_3_), 79.9 (COC̲(CH_3_)_3_), 82.3 (COC̲(CH_3_)_3_), 106.8, 109.7, 111.0, 114.2, 120.4, 129.3, 130.5, 130.6, 136.2, 139.2, 149.2, 154.1, 155.0 (Ar), 155.8 (CO), 159.2 (Ar), 171.7 (CO).

#### 4.1.2 (*S*)-2-Amino-4-((3-(6-amino-9*H*-purin-9-yl)phenyl)(3,3-dimethylbutyl)amino)butanoic acid (10)

Compound 9 (176 mg, 0.26 mmol) was stirred in 2.6 mL of TFA at 70 °C for 16 h. Then, volatiles were removed. The residue obtained was treated with dichloromethane and concentrated to dryness. This procedure was repeated several times. The solid obtained was triturated in Et_2_O, then purified by flash chromatography (C18), (gradient 0–50% acetonitrile in water), obtaining 102 mg (76% yield) of 10 as a white solid, corresponding to the compound as TFA salt. MS (ES, positive mode): *m*/*z* 412 (M + H)^+^. ^1^H NMR (400 MHz, DMSO-*d*_6_) *δ*: 0.94 (s, 9H, (CH_3_)_3_), 1.43–1.51 (m, 2H, CH̲_2_*t*Bu), 1.95–2.12 (m, 2H, CH_2_), 3.29–3.39 (m, 2H, NCH̲_2_CH_2_*t*Bu), 3.44 (m, 1H, CH_2_), 3.54 (m, 1H, CH_2_), 3.84 (m, 1H, Hα), 6.71 (d, *J* = 8.3 Hz, 1H, Ar), 7.01 (d, *J* = 7.7 Hz, 1H, Ar), 7.16 (s, 1H, Ar), 7.31 (t, *J* = 8.1 Hz, 1H, Ar), 7.36 (br s, 2H, 6-NH_2_), 8.17 (s, 1H, H_2_/H_8_), 8.53 (s, 1H, H_2_/H_8_) ^13^C NMR (100 MHz, DMSO-*d*_6_) *δ*: 27.9 (CH_2_), 29.2 (C(C̲H_3_)_3_), 29.6 (C̲(CH_3_)_3_), 46.5 (CH_2_), 46.6 (NC̲H_2_CH_2_*t*Bu), 50.8 (Cα), 106.0, 109.4, 110.3 (Ar), 118.8 (q, *J*_C–F_ = 298.9 Hz, CF_3_-TFA), 119.4, 130.1, 136.3, 139.8, 148.2, 149.2, 153.0, 156.3 (Ar), 158.3 (q, *J* = 28.9 Hz, COCF_3._-TFA), 171.0 (COOH). HRMS (ESI): calcd for C_21_H_29_N_7_O_2_ 411.2383, found 411.2390.

#### 4.1.3 *tert*-Butyl (*S*)-2-((*tert*-butoxycarbonyl)amino)-4-((3,3-dimethylbutyl)(3-(4-methyl-7*H*-pyrrolo[2,3-*d*]pyrimidin-7-yl)phenyl)amino)butanoate (13)

A solution containing 12 (ref. 29) (188 mg, 0.39 mmol) and 3,3-dimethylbutyraldehyde (196 mg, 1.95 mmol) in MeOH (8 mL) was recirculated through a 30 mm CatCart™ in the H-cube at 60 Bar, 100% of hydrogen production, at 150 °C and a flow rate 0.5 mL min^−1^. After 4 h of recirculation, volatiles were removed and the crude was purified by CCTLC (DCM : MeOH 20 : 1) to yield 55 mg (25% yield) of 13 as a white solid. Mp: 59–61 °C. MS (ES, positive mode): *m*/*z* 566 (M + H)^+^. ^1^H NMR (400 MHz, CDCl_3_) *δ*: 0.97 (s, 9H, (CH_3_)_3_), 1.40 (s, 9H, OC(CH_3_)_3_), 1.45 (s, 9H, OC(CH_3_)_3_), 1.53 (m, 2H, CH_2_), 1.95 (m, 1H, CH_2_), 2.15 (m, 1H, CH_2_), 2.78 (s, 3H, CH_3_), 3.25–3.46 (m, 4H, CH_2_, CH_2_), 4.20 (m, 1H, Hα), 5.35 (br d, *J* = 8.2 Hz, 1H, NH), 6.62 (dd, *J* = 8.4, 2.5 Hz, 1H, Ar), 6.69 (d, *J* = 3.7 Hz, 1H, Ar), 6.83 (dd, *J* = 7.5 Hz, 1.9 Hz, 1H, Ar), 7.09 (t, *J* = 2.3 Hz, 1H, Ar), 7.31 (t, *J* = 8.1 Hz, 1H, Ar), 7.50 (d, *J* = 3.7 Hz, 1H, Ar), 8.83 (s, 1H, Ar). ^13^C NMR (100 MHz, CDCl_3_) *δ*: 21.6 (CH_3_), 28.1, (C(C̲H_3_)_3_) 28.4 (C(C̲H_3_)_3_), 29.5 (C(C̲H_3_)_3_), 29.9 (C̲(CH_3_)_3_), 30.3 (CH_2_), 39.9 (CH_2_), 47.4 (CH_2_), 47.5 (CH_2_), 52.6 (Cα), 79.9 (C̲(CH_3_)_3_), 82.3 (C̲(CH_3_)_3_), 100.6, 107.7, 110.4, 110.7, 118.9, 128.2, 130.3, 138.7, 148.6, 150.2, 151.9 (Ar), 155.7 (CO), 159.6 (Ar), 171.7 (CO).

#### 4.1.4 (*S*)-2-Amino-4-((3,3-dimethylbutyl)(3-(4-methyl-7*H*-pyrrolo[2,3-*d*]pyrimidin-7-yl)phenyl)amino)butanoic acid (14)

To a cooled solution of 13 (13 mg, 0.02 mmol) in DCM (0.23 mL), trifluoroacetic acid (90 μL, 1.15 mmol) was added. The resulting mixture was stirred at 25 °C for 2 h. Volatiles were removed by co-evaporation with chloroform, and the solid obtained was lyophilized to yield 12 mg (quantitative yield) of 14 as a white solid, corresponding to the compound as TFA salt. MS (ES, positive mode): *m*/*z* 410 (M + H)^+^. ^1^H NMR (400 MHz, DMSO-*d*_6_) *δ*: 0.96 (s, 9H, (CH_3_)_3_), 1.48 (m, 2H, CH_2_), 2.07 (m, 1H, CH_2_), 2.81 (s, 3H, CH_3_), 3.35 (m, 2H, CH_2_), 3.42 (m, 1H, CH_2_), 3.54 (m, 1H, CH_2_), 4.03 (d, *J* = 6.6 Hz, 1H, Hα), 6.72 (dd, *J* = 8.5, 2.4 Hz, 1H, Ar), 6.97 (dd, *J* = 7.7, 1.8 Hz, 1H, Ar), 7.10 (d, *J* = 3.7 Hz, 1H, Ar), 7.11 (t, *J* = 2.3 Hz, 1H, Ar), 7.35 (t, *J* = 8.2 Hz, 1H, Ar), 8.06 (d, *J* = 3.8 Hz, 1H, Ar), 8.33 (br s, 3H, NH_3_^+^), 8.85 (s, 1H, Ar). ^13^C NMR (100 MHz, DMSO-*d*_6_) *δ*: 19.7 (CH_3_), 27.6 (CH_2_), 29.2 (C(C̲H_3_)_3_), 29.5 (C̲(CH_3_)_3_), 39.6 (CH_2_), 46.3 (CH_2_), 46.5 (CH_2_), 50.1 (Cα), 101.8, 107.1, 110.4, 110.6, 116.2 (*J*_C–F_ = 294.4 Hz, CF_3_-TFA), 118.3, 130.1, 130.7, 137.8, 148.0, 148.6, 149.2, 157.9 (Ar), 158.0 (*J*_C–F_ = 32.2 Hz, C̲OCF_3_-TFA), 171.0 (CO). HRMS (ESI): calcd for C_23_H_31_N_5_O_2_ 409.2478, found 409.2478.

#### 4.1.5 Methyl (*S*)-2-((*tert*-butoxycarbonyl)amino)-4-((3-(4-methyl-7*H*-pyrrolo[2,3-*d*]pyrimidin-7-yl)phenyl)amino)butanoate (15)

A solution containing compound 11 (ref. 29) (150 mg, 0.67 mmol) and methyl (*S*)-2-((*tert*-butoxycarbonyl)amino)-4-oxobutanoate (whose synthesis is described in the SI) (187 mg, 0.81 mmol) in MeOH (13 mL) was recirculated through a 30 mm CatCart™ in the H-cube at 40 Bar, 100% of hydrogen production, at 65 °C and a flow rate 0.5 mL min^−1^. After 2 h, volatiles were removed, and the residue was purified by CCTLC (hexane : EtOAc, 1 : 3) to yield 120 mg (41% yield) of 15 as a white solid. Mp: 67–69 °C. MS (ES, positive mode): *m*/*z* 440 (M + H)^+^. ^1^H NMR (400 MHz, CDCl_3_) *δ*: 1.44 (s, 9H, (CH_3_)_3_), 1.87 (m, 1H, CH_2_), 2.20 (m, 1H, CH_2_), 2.77 (s, 3H, CH_3_), 3.27 (m, 1H, CH_2_), 3.36 (m, 1H, CH_2_), 3.71 (s, 3H, OCH_3_), 4.35 (br s, 1H, NH), 4.44 (m, 1H, Hα), 5.28 (d, *J* = 8.2 Hz, 1H, NHBoc), 6.61 (dd, *J* = 8.2, 2.3 Hz, 1H, Ar), 6.68 (d, *J* = 3.7 Hz, 1H, Ar), 6.93–7.00 (m, 2H, Ar), 7.30 (t, *J* = 8.0 Hz, 1H, Ar), 7.46 (d, *J* = 3.7 Hz, 1H, Ar), 8.82 (s, 1H, Ar). ^13^C NMR (100 MHz, CDCl_3_) *δ*: 21.7 (CH_3_), 28.4 (C(C̲H_3_)_3_), 32.5 (CH_2_), 39.9 (CH_2_), 51.5 (Cα), 52.6 (OCH_3_), 80.4 (C̲(CH_3_)_3_), 100.5, 108.4, 111.7, 112.8, 118.9, 128.0, 130.4, 138.7, 148.9, 150.2, 152.1 (Ar), 155.8 (CO), 159.8 (Ar), 173.2 (CO).

#### 4.1.6 Methyl (*S*)-2-((*tert*-butoxycarbonyl)amino)-4-((3,3-dimethylbutyl)(3-(4-methyl-7*H*-pyrrolo[2,3-*d*]pyrimidin-7-yl)phenyl)amino)butanoate (16)

A solution containing 15 (260 mg, 0.59 mmol) and 3,3-dimethylbutyraldehyde (119 mg, 1.18 mmol) in MeOH (12 mL) was recirculated through a 30 mm CatCart™ in the H-cube at 60 Bar, 100% of hydrogen production, at 150 °C and a flow rate 0.5 mL min^−1^. After 6 hours of recirculation, volatiles were removed and the crude was purified by CCTLC (Hex : EtOAc, 1 : 1) to yield 153 mg (50% yield) of 16 as a white solid. Mp: 78–80 °C. MS (ES, positive mode): *m*/*z* 524 (M + H)^+^. ^1^H NMR (400 MHz, CDCl_3_) *δ*: *δ* 0.97 (s, 9H, (CH_3_)_3_), 1.40 (s, 9H, OC(CH_3_)_3_), 1.52 (m, 2H, CH_2_), 1.99 (m, 1H, CH_2_), 2.21 (m, 1H, CH_2_), 2.78 (s, 3H, CH_3_), 3.30–3.46 (m, 4H, CH_2_), 3.71 (s, 3H, OCH_3_), 4.34 (m, 1H, Hα), 5.40 (d, *J* = 8.4 Hz, 1H, NH), 6.62 (dd, *J* = 8.3, 2.6 Hz, 1H, Ar), 6.70 (d, *J* = 3.7 Hz, 1H, Ar), 6.83 (dd, *J* = 7.8, 1.9 Hz, 1H, Ar), 7.11 (t, *J* = 2.3 Hz, 1H, Ar), 7.31 (t, *J* = 8.1 Hz, 1H, Ar), 7.50 (d, *J* = 3.7 Hz, 1H, Ar), 8.84 (s, 1H, Ar). ^13^C NMR (100 MHz, CDCl_3_) *δ*: 21.3 (CH_3_), 28.4 (C(C̲H_3_)_3_), 29.5 (C(C̲H_3_)_3_), 29.9 (C̲(CH_3_)_3_), 30.2 (CH_2_), 39.9 (CH_2_), 47.4 (CH_2_), 47.7(CH_2_), 52.0 (Cα), 52.6 (OCH_3_), 80.2 (OC̲(CH_3_)_3_), 100.8, 107.9, 110.7, 110.8, 118.9, 128.5, 130.3, 138.6, 148.5, 150.1, 151.4 (Ar), 155.7 (CO), 159.4 (Ar), 173.0 (CO).

#### 4.1.7 *tert*-Butyl (*S*)-(1-amino-4-((3,3-dimethylbutyl)(3-(4-methyl-7*H*-pyrrolo[2,3-*d*]pyrimidin-7-yl)phenyl)amino)-1-oxobutan-2-yl)carbamate (17)

Compound 16 (100 mg, 0.19 mmol) was dissolved in a solution of NH_3_ in MeOH 7N (2 mL). The reaction was stirred at 25 °C. After 4 days, volatiles were removed and the solid obtained was lyophilized. The solid was dissolved in DCM, treated with petroleum ether and isolated by filtration to yield 32 mg (33% yield) of 17, as a white solid. Mp: 107–109 °C. MS (ES, positive mode): *m*/*z* 509 (M + H)^+^. ^1^H NMR (400 MHz, CDCl_3_) *δ*: 0.96 (s, 9H, (CH_3_)_3_), 1.40 (s, 9H, OC(CH_3_)_3_), 1.51 (m, 2H, CH_2_), 1.95 (m, 1H, CH_2_), 2.26 (m, 1H, CH_2_), 2.81 (s, 3H, CH_3_), 3.36 (m, 2H, CH_2_), 3.43 (m, 2H, CH_2_), 4.22 (m, 1H, H_α_), 5.39 (br d, *J* = 8.1 Hz, 1H, NH̲Boc), 5.45 (br s, 1H, CONH_2_), 6.37 (br s, 1H, CONH_2_), 6.67 (dd, *J* = 8.5, 2.5 Hz, 1H, Ar), 6.72 (d, *J* = 3.8 Hz, 1H, Ar), 6.83 (d, *J* = 7.8 Hz, 1H, Ar), 7.13 (t, *J* = 2.3 Hz, 1H, Ar), 7.32 (t, *J* = 8.3 Hz, 1H, Ar), 7.54 (d, *J* = 3.7 Hz, 1H, Ar), 8.82 (s, 1H, Ar). ^13^C NMR (100 MHz, CD_3_OD) *δ*: 20.9 (CH_3_), 28.7 (C(C̲H_3_)_3_), 29.8 (C(C̲H_3_)_3_), 30.6 (C̲(CH_3_)_3_), 31.1 (CH_2_), 40.6 (CH_2_), 47.0 (CH_2_), 53.8 (Cα), 80.7 (OC̲(CH_3_)_3_), 101.7, 109.1, 111.8, 111.9, 120.3, 130.5, 131.2, 139.7, 150.0, 151.0, 151.9 (Ar), 157.9 (CO), 160.9 (Ar), 177.6 (CO). HRMS (ESI): calcd for C_28_H_40_N_6_O_3_ 508.3162, found 508.3162.

#### 4.1.8 (*S*)-2-Amino-4-((3,3-dimethylbutyl)(3-(4-methyl-7*H*-pyrrolo[2,3-*d*]pyrimidin-7-yl)phenyl)amino) butanamide (18)

Compound 17 (40 mg, 0.08 mmol) was dissolved in a solution of HCl 4N in 1,4-dioxane (4 mL). The reaction was stirred at 25 °C for 1 h. Volatiles were removed and the solid obtained was lyophilized to yield 25 mg (72% yield) of 18 as a white solid, corresponding to the compound as HCl salt. MS (ES, positive mode): *m*/*z* 409 (M + H)^+^. ^1^H NMR (400 MHz, DMSO-*d*_6_ + D_2_O) *δ*: 0.94 (s, 9H, (CH_3_)_3_), 1.45 (m, 2H, CH_2_), 2.04 (m, 2H, CH_2_), 2.98 (s, 3H, CH_3_), 3.34 (m, 2H, CH_2_), 3.42 (m, 2H, CH_2_), 3.86 (t, *J* = 6.2 Hz, 1H, Hα), 6.79 (d, *J* = 8.5 Hz, 1H, Ar), 6.96 (d, *J* = 7.9 Hz, 1H, Ar), 7.01 (br s, 1H, CONH_2_), 7.33–7.42 (m, 2H, Ar), 8.31 (s, 1H, Ar), 9.10 (s, 1H, Ar). ^13^C NMR (100 MHz, DMSO-d_6_ + D_2_O) *δ*: 17.6 (CH_3_), 28.2 (CH_2_), 29.3 (C(C̲H_3_)_3_), 29.7 (C̲(CH_3_)_3_), 39.5 (CH_2_), 46.4 (CH_2_), 46.7 (CH_2_), 50.4 (Cα), 103.2, 107.6, 111.5, 118.3, 130.4, 134.0, 137.1, 144.7, 148.0, 149.1, 155.1 (Ar), 170.1 (CO). HRMS (ESI): calcd for C_23_H_32_N_6_O 408.2638, found 408.2642.

#### 4.1.9 *tert*-Butyl *N*^2^-(((9*H*-fluoren-9-yl)methoxy)carbonyl)-*N*^4^-(3-(4-methyl-7*H*-pyrrolo[2,3-*d*]pyrimidin-7-yl)phenyl)-l-asparaginate (19)

To a solution of 11 (ref. 29) (300 mg, 1.37 mmol), Fmoc-Asp-O^*t*^Bu (605 mg, 1.47 mmol) and HATU (762 mg, 2.00 mmol) in anhydrous DMF (13 mL), DIPEA was added (466 μL, 2.67 mmol) and the mixture was stirred for 2.5 h at 40 °C. The reaction was quenched by adding a few drops of a saturated solution of NH_4_Cl and the solvent was removed *in vacuo*. The residue was dissolved in ethyl acetate (20 mL) and washed with a saturated solution of NH_4_Cl (10 mL). The organic layer was dried over Na_2_SO_4_, filtered and evaporated to dryness. The crude was purified by flash chromatography (DCM : MeOH 20 : 1) to yield 415 mg (50% yield) of 19 as a white solid. Mp: 117–119 °C. MS (ES, positive mode): *m*/*z* 618 (M + H)^+^. ^1^H NMR (400 MHz, CDCl_3_) *δ*: 1.46 (s, 9H, (CH_3_)_3_), 2.76 (s, 3H, CH_3_), 2.90 (dd, *J* = 16.0, 4.5 Hz, 1H, CH_2_β), 3.05 (dd, *J* = 16.3, 4.8 Hz, 1H, CH_2_β), 4.19 (t, *J* = 7.2 Hz, 1H, 
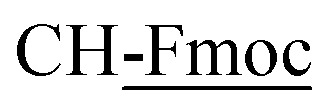
), 4.36 (m, 2H, CH_2_-Fmoc), 4.54 (m, 1H, Hα), 6.11 (d, *J* = 8.0 Hz, 1H, NH̲Fmoc), 6.68 (d, *J* = 3.7 Hz, 1H, Ar), 7.20–7.28 (m, 2H, Ar), 7.35 (t, *J* = 7.6 Hz, 2H, Ar), 7.38–7.41 (m, 2H, Ar), 7.43 (d, *J* = 3.7 Hz, 1H, Ar), 7.50 (m, 1H, Ar), 7.56 (dd, *J* = 7.5, 2.2 Hz, 2H, Ar), 7.72 (d, *J* = 7.6 Hz, 2H, Ar), 8.00 (s, 1H, Ar), 8.62 (br s, 1H, NHCO), 8.80 (s, 1H, Ar). ^13^C NMR (100 MHz, CDCl_3_) *δ*: 22.7 (CH_3_), 28.1 (C(C̲H_3_)_3_), 39.6 (CH_2_β), 47.2 
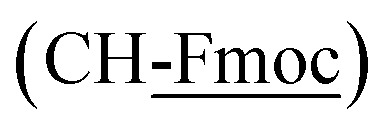
, 51.5 (Cα), 67.4 (CH_2_-Fmoc), 83.0 (OC̲(CH_3_)_3_), 101.1, 115.5, 118.3, 119.0, 119.2, 120.4, 125.3, 127.2, 127.8, 127.9, 130.1, 138.2, 138.9, 141.4, 143.9, 150.3, 152.0 (Ar), 156.5 (CO), 160.1 (Ar), 168.2 (CO), 169.9 (CO).

#### 4.1.10 *tert*-Butyl *N*^4^-(3-(4-methyl-7*H*-pyrrolo[2,3-*d*]pyrimidin-7-yl)phenyl)-l-asparaginate (20)

To a solution of 19 (300 mg, 0.49 mmol) in DCM (5 mL), piperidine (480 μL, 4.86 mmol) was added and the mixture was stirred at 25 °C for 2 h. The crude was concentrated *in vacuo* and directly purified by CCTLC (DCM : MeOH, 10 : 1) to yield 133 mg (70% yield) of 20 as an amorphous solid. MS (ES, positive mode): *m*/*z* 396 (M + H)^+^. ^1^H NMR (400 MHz, CDCl_3_) *δ*: 1.47 (s, 9H, (CH_3_)_3_), 1.95 (br s, 2H, NH_2_), 2.58 (m, 1H, CH_2_β), 2.66–2.86 (m, 4H, CH_2_β, CH_3_), 3.78 (dd, *J* = 9.7, 2.9 Hz, 1H, Hα), 6.70 (d, *J* = 3.6 Hz, 1H, Ar), 7.49 (d, *J* = 3.7 Hz, 1H, Ar), 8.06 (s, 1H, Ar), 8.81 (s, 1H, Ar), 10.31 (br s, 1H, NH). ^13^C NMR (100 MHz, CDCl_3_) *δ*: 21.7 (CH_3_), 28.1 (C(C̲H_3_)_3_), 40.5 (CH_2_β), 51.9 (Cα), 82.5 (OC̲(CH_3_)_3_), 100.9, 113.5, 118.3, 118.9, 119.4, 127.9, 130.0, 138.1, 139.5, 150.2, 152.1, 160.0 (Ar), 169.2 (CO), 173.2 (CO). HRMS (ESI): calcd for CH_17_N_5_O_3_ 395.1957, found 395.1956.

#### 4.1.11 *N* ^4^-(3-(4-Methyl-7*H*-pyrrolo[2,3-*d*]pyrimidin-7-yl)phenyl)-l-asparagine (21)

To a cooled solution of 20 (80 mg, 0.20 mmol) in DCM (2 mL), trifluoroacetic acid (774 μL, 10.12 mmol) was added. The resulting mixture was stirred at 25 °C for 16 h. Volatiles were removed and the solid obtained was lyophilized to yield 90 mg (quantitative yield) of 21 as a white solid, isolated as a TFA salt. MS (ES, positive mode): *m*/*z* 341 (M + H)^+^. ^1^H NMR (400 MHz, DMSO-*d*_6_) *δ*: 2.81 (s, 3H, CH_3_), 3.02 (t, *J* = 5.3 Hz, 2H, CH_2_β), 4.30 (m, 1H, Hα), 7.10 (d, *J* = 3.7 Hz, 1H, Ar), 7.48 (ddd, *J* = 8.0, 2.2, 1.2 Hz, 1H, Ar), 7.54 (t, *J* = 8.0 Hz, 1H, Ar), 7.64 (ddd, *J* = 8.1, 2.1, 1.2 Hz, 1H, Ar), 8.03 (d, *J* = 3.8 Hz, 1H, Ar), 8.16 (t, *J* = 2.1 Hz, 1H, Ar), 8.30 (br s, 3H, NH_3_^+^), 8.87 (s, 1H, Ar), 10.58 (br s, 1H, NHCO). ^13^C NMR (100 MHz, DMSO-*d*_6_) *δ*: 20.4 (CH_3_), 35.9 (CH_2_β), 48.5 (Cα), 101.8, 114.7, 116.5 (*J*_C–F_ = 297.1 Hz, COCF_3_-TFA), 117.8, 118.3, 118.9, 129.5, 129.9, 137.2, 139.5, 149.4, 150.0, 158.1 (*J*_C–F_ = 34.2 Hz, COCF_3_-TFA), 158.6 (CONH), 167.6 (Ar), 170.2 (CO). HRMS (ESI): calcd for C_17_H_17_N_5_O_3_ 339.1331, found 339.1331.

#### 4.1.12 6-Chloro-*N*^4^-(2-methoxy-5-nitrophenyl)pyrimidine-4,5-diamine (24)

A solution of 4,6-dichloropyrimidin-5-amine (23) (500 mg, 3.05 mmol), 2-methoxy-5-nitroaniline (513 mg, 3.05 mmol) and concentrated HCl (150 μL) in 12 mL of isobutanol was heated under MW at 150 °C for 1 h. The resulting solid was filtered and washed with isobutanol, and later with hexane, affording 413 mg (46% yield) of 24 as a clear brown solid. Mp: 91–93 °C. MS (ES, positive mode): *m*/*z* 296 (M + H)^+^, with a Cl isotopic pattern. ^1^H NMR (400 MHz, DMSO-*d*_6_) *δ*: 3.97 (s, 3H, OCH_3_), 6.47 (br s, 2H, NH_2_), 7.31 (d, *J* = 9.2 Hz, 1H, Ar), 7.92 (s, 1H, Ar), 8.07 (dd, *J* = 9.1, 2.8 Hz, 1H, Ar), 8.73 (d, *J* = 2.8 Hz, 1H, Ar), 8.77 (br s, 1H, NH). ^13^C NMR (100 MHz, DMSO-*d*_6_) *δ*: 56.8 (OCH_3_), 111.1, 117.5, 120.1, 125.4, 128.5, 139.9, 140.4, 145.1, 149.1, 155.7 (Ar).

#### 4.1.13 6-Chloro-9-(2-methoxy-5-nitrophenyl)-9*H*-purine (25)

A solution of 24 (822 mg, 2.78 mmol) in trimethyl orthoformate (5.6 mL) and concentrated HCl (220 μL) was heated under MW at 120 °C for 1 h. The resulting solid was filtered and washed with hexane, affording 705 mg (83% yield) of 25 as a clear brown solid. Mp: 241–242 °C MS (ES, positive mode): *m*/*z* 306 (M + H)^+^. ^1^H NMR (400 MHz, CDCl_3_) *δ*: 3.95 (s, 3H, OCH_3_), 7.58 (d, *J* = 9.3 Hz, 1H, Ar), 8.48 (m, 1H, Ar), 8.64 (d, *J* = 2.8 Hz, 1H, Ar), 8.81 (s, 1H, H_2_/H_8_), 8.92 (s, 1H, H_2_/H_8_). ^13^C NMR (100 MHz, CDCl_3_) *δ*: 57.4 (OCH_3_), 113.3, 122.1, 124.2, 126.9, 130.6, 140.3, 147.6, 149.5, 152.3, 152.4, 159.1 (Ar).

#### 4.1.14 9-(2-Methoxy-5-nitrophenyl)-*N*-(4-methoxybenzyl)-9*H*-purin-6-amine (26)

A solution of 25 (684 mg, 2.24 mmol), DIPEA (189 μL, 2.24 mmol) and (4-methoxyphenyl)methanamine (293 μL, 2.24 mmol) in isopropanol (11.2 mL) was heated under MW at 120 °C for 30 minutes. The resulting solid was filtered and washed with isopropanol, then with hexane, affording 681 mg (75% yield) of 26 as a beige solid. Mp: 214–215 °C MS (ES, positive mode): *m*/*z* 407 (M + H)^+^. ^1^H NMR (400 MHz, CDCl_3_) *δ*: 3.71 (s, 3H, OCH_3_), 3.94 (s, 3H, OCH_3_), 4.66 (br s, 2H, CH_2_), 6.87 (d, *J* = 8.5 Hz, 2H, Ar), 7.31 (d, *J* = 8.3 Hz, 2H, Ar), 7.52 (d, *J* = 9.3 Hz, 1H, Ar), 8.22 (br s, 1H, NH), 8.36 (s, 1H, H_2_/H_8_), 8.39–8.47 (m, 2H, Ar, H_2_/H_8_), 8.51 (d, *J* = 2.7 Hz, 1H, Ar). ^13^C NMR (100 MHz, CDCl_3_) *δ*: 42.4 (CH_2_), 55.0 (OCH_3_), 57.2 (OCH_3_), 113.1, 113.6, 118.5, 123.2, 123.7, 126.0, 128.6, 132.0, 140.3, 141.0, 149.4, 153.1, 154.5, 158.1, 159.0 (Ar).

#### 4.1.15 9-(5-Amino-2-methoxyphenyl)-*N*-(4-methoxybenzyl)-9*H*-purin-6-amine (22)

A solution of 26 (564 mg, 1.39 mmol), and tin(ii) chloride (1.33 g, 7.00 mmol) in a mixture of AcOEt : EtOH (2 : 1) (7.5 mL) was heated at 80 °C for 1 h. Then, it was cooled and the reaction was basified until pH 10 with 10 mL of 2.5 M NaOH in water. Later, the slurry was filtered through a Celite pad and washed with MeOH. Volatiles were removed, and the residue was sonicated 15 minutes with 50 mL of AcOEt and 10 mL of brine solution. Then, the aqueous phase was extracted with AcOEt, dried over anhydrous sodium sulfate, and concentrated under reduced pressure. The residue was purified by flash chromatography (20–100% ethyl acetate in hexane), obtaining 368 mg (60% yield) of 22 as an amorphous solid. MS (ES, positive mode): *m*/*z* 377 (M + H)^+^. ^1^H NMR (400 MHz, DMSO-*d*_6_) *δ*: 3.61 (s, 3H, OCH_3_), 3.71 (s, 3H, OCH_3_), 4.66 (br s, 2H, CH_2_), 4.97 (br s, 2H, NH_2_), 6.66–6.74 (m, 2H, Ar), 6.99 (d, *J* = 8.9 Hz, 1H, Ar), 7.31 (d, *J* = 8.6 Hz, 2H, Ar), 8.18 (m, 2H, H_2_/H_8_), 8.31 (br s, 1H, NH). ^13^C NMR (100 MHz, DMSO-*d*_6_) *δ*: 42.4 (CH_2_), 55.0 (OCH_3_), 56.5 (OCH_3_), 113.6, 113.7, 114.3, 114.9, 118.6, 123.4, 128.6, 132.1, 141.4, 142.9, 144.7, 149.5, 152.8, 154.5, 158.1 (Ar).

#### 4.1.16 *tert*-Butyl *N*^2^-(((9*H*-fluoren-9-yl)methoxy)carbonyl)-*N*^4^-(3-(6-((4-methoxybenzyl)amino)-9*H*-purin-9-yl)phenyl)-l-asparaginate (27)

To a solution of 6 (165 mg, 0.48 mmol), Fmoc-Asp-O^t^Bu (216 mg, 0.52 mmol) and HATU (272 mg, 0.714 mmol) in anhydrous DMF (5 mL), DIPEA was added (87 μL, 0.95 mmol) and the mixture was stirred for 2.5 h at 40 °C. The reaction was quenched by adding a few drops of a saturated solution of NH_4_Cl and the solvent was removed *in vacuo*. The residue was dissolved in ethyl acetate (20 mL) and washed with a saturated solution of NH_4_Cl (10 mL). The organic layer was dried over Na_2_SO_4_, filtered and evaporated to dryness. The crude was purified by chromatography on silica gel column (DCM : MeOH, 60 : 1) to yield 195 mg (55% yield) of 27 as a white solid. Mp: 134–136 °C MS (ES, positive mode): *m*/*z* 749 (M + H)^+^. ^1^H NMR (400 MHz, DMSO-*d*_6_) *δ*: 1.36 (s, 9H, (CH_3_)_3_), 2.70 (m, 1H,CH_2_β), 2.88 (m, 1H, CH_2_β), 3.71 (s, 3H, OCH_3_), 4.22 (t, *J* = 6.8 Hz, 1H, 
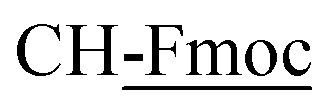
), 4.31 (m, 2H, 
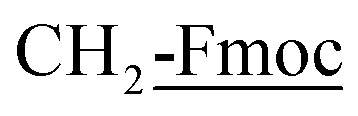
), 4.41 (q, *J* = 7.3 Hz, 1H, Hα), 4.66 (s, 2H, ArCH̲_2_NH), 6.87 (m, 2H, Ar), 7.25–7.34 (m, 4H, Ar), 7.39 (m, 2H, Ar), 7.44–7.55 (m, 2H, Ar), 7.65–7.73 (m, 3H, Ar), 7.75 (br d, *J* = 8.2 Hz, 1H, CHNH̲), 7.87 (m, 2H, Ar), 8.16 (s, 1H, Ar), 8.27 (s, 1H, H_2_/H_8_), 8.44 (br s, 1H, ArCH_2_NH̲), 8.52 (s, 1H, H_2_/H_8_), 10.30 (br s, 1H, CONH). ^13^C NMR (100 MHz, DMSO-*d*_6_) *δ*: 27.6 (C(C̲H_3_)_3_), 38.3 (CH_2_β), 42.4 (ArC̲H_2_NH), 46.6 
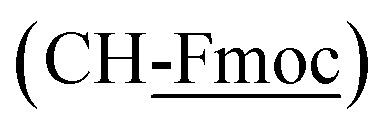
, 51.1 (Cα), 55.6 (OCH_3_), 65.6 
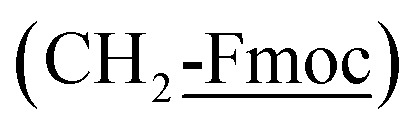
, 80.8 (OC̲(CH_3_)_3_), 113.6, 113.8, 117.8, 118.0, 120.1, 125.2, 127.0, 127.6, 128.6, 128.8, 131.9, 135.2, 139.5, 140.0, 140.7, 143.8, 148.0, 153.1, 154.6 (Ar), 155.8 (CO), 158.1 (Ar), 168.2 (CO), 170.5 (CO).

#### 4.1.17 *tert*-Butyl *N*^2^-(((9*H*-fluoren-9-yl)methoxy)carbonyl)-*N*^4^-(4-methoxy-3-(6-((4-methoxybenzyl)amino)-9*H*-purin-9-yl)phenyl)-l-asparaginate (28)

A solution of 22 (335 mg, 0.89 mmol), Fmoc-Asp-OtBu (403 mg, 0.98 mmol), HATU (508 mg, 1.34) and DIPEA (150 μL, 1.78 mmol) in anhydrous DMF (8.9 mL) was heated at 40 °C for 2.5 h. Then, it was cooled, quenched with a saturated solution of NH_4_Cl and extracted with AcOEt. The organic layer was dried over Na_2_SO_4_, filtered and concentrated under reduced pressure. The residue was purified by flash chromatography (gradient 40–80% ethyl acetate in hexane) to afford 517 mg (75% yield) of 28 as a white solid. Mp: 103–105 °C. MS (ES, positive mode): *m*/*z* 770 (M + H)^+^. ^1^H NMR (400 MHz, DMSO-*d*_6_) *δ*: 1.36 (s, 9H, (CH_3_)_3_), 2.66 (dd, *J* = 15.4, 7.4, Hz, 1H, CH_2_β), 2.82 (dd, *J* = 15.4, 6.4, Hz, 1H, CH_2_β), 3.71 (s, 3H, OCH_3_), 3.74 (s, 3H, OCH_3_), 4.24 (m, 1H, 
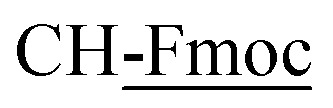
), 4.30 (d, *J* = 6.7 Hz, 2H, 
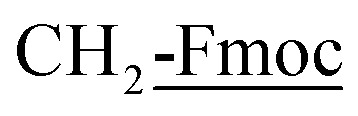
), 4.39 (q, *J* = 7.4 Hz, 1H, Hα), 4.66 (br s, 2H, ArCH̲_2_NH), 6.87 (d, *J* = 8.7 Hz, 2H, Ar), 7.22–7.35 (m, 5H, Ar), 7.39 (t, *J* = 7.5 Hz, 2H, Ar), 7.65–7.75 (m, 4H, Ar), 7.77 (d, *J* = 2.5 Hz, 1H, CONH), 7.87 (d, *J* = 7.5 Hz, 2H, Ar), 8.18 (s, 1H, H_2_/H_8_), 8.24 (s, 1H, H_2_/H_8_), 8.37 (s, 1H, NH), 10.11 (s, 1H, CONH).^13^C NMR (100 MHz, DMSO-*d*_6_) *δ*: 27.6 (C(C̲H_3_)_3_), 38.2 (CH_2_β), 42.4 (ArC̲H_2_NH), 46.6 
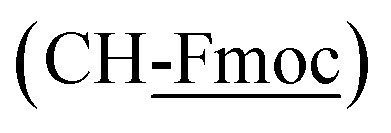
, 51.2 (Cα), 55.0 (OCH_3_), 56.1 (OCH_3_), 65.7 
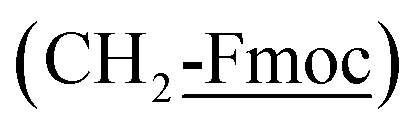
, 80.8 (OC̲(CH_3_)_3_), 112.9, 113.6, 118.5, 119.1, 120.1, 120.6, 122.5, 125.2, 127.1, 127.6, 128.6, 132.1, 132.3, 140.7, 141.3, 143.8, 149.3, 149.6, 153.0, 154.5 (Ar), 155.8 (CO), 158.1 (Ar), 167.7, 170.6 (CO).

#### 4.1.18 *tert*-Butyl *N*^4^-(3-(6-amino-9*H*-purin-9-yl)phenyl)-l-asparaginate (29)

A mixture of 27 (224 mg, 0.30 mmol) and (NH_4_)_2_S_2_O_8_ (138 mg, 0.60 mmol) in PBS : CH_3_CN (1 : 1) (3.0 mL) was heated in a microwave reactor at 80 °C for 45 min. The crude was dissolved in ethyl acetate (20 mL) and washed with a saturated solution of NaHCO_3_ (10 mL). The organic layer was dried over Na_2_SO_4_, filtered and evaporated to dryness. The residue was purified by CCTLC (DCM : MeOH, 10 : 1) to provide a compound with *m*/*z* = 620 (M + H)^+^. To a solution of this compound (62 mg, 0.10 mmol) in DCM (6 mL), piperidine (98 μL, 1.0 mmol) was added. The mixture was stirred at 25 °C for 2 h. The crude was concentrated *in vacuo* and directly purified by column chromatography on silica gel (DCM : MeOH : NH_3_ 20 : 1 : 0.2) to yield 30 mg (25% yield) of 29 as a white solid. Mp: 150–152 °C. MS (ES, positive mode): *m*/*z* 398 (M + H)^+^. ^1^H NMR (400 MHz, DMSO-*d*_6_) *δ*: 1.39 (s, 9H, (CH_3_)_3_), 1.96 (br s, 2H, NH_2_), 2.55 (m, 1H, CH_2_β), 2.65 (dd, *J* = 14.9, 5.7 Hz, 1H, CH_2_β), 3.63 (t, *J* = 6.6 Hz, 1H, Hα), 7.39 (br s, 2H, NH_2_), 7.44–7.54 (m, 2H, Ar), 7.67 (dt, *J* = 7.8, 1.7 Hz, 1H, Ar), 8.13 (t, *J* = 2.0 Hz, 1H, Ar), 8.20 (s, 1H, H_2_/H_8_), 8.51 (s, 1H, H_2_/H_8_), 10.30 (br s, 1H, CONH). ^13^C NMR (100 MHz, MeOH-*d*_4_) *δ*: 28.2 (C(C̲H_3_)_3_), 41.6 (CH_2_β), 52.9 (Cα), 82.8 (OC̲(CH_3_)_3_), 116.5, 120.3, 120.5, 120.6, 131.2, 136.4,141.3, 141.7, 150.5, 154.4, 157.7 (Ar), 171.4 (CO), 174.6 (CO). HRMS (ESI): calcd for C_19_H_23_N_7_O_3_ 397.1862, found 397.1861.

#### 4.1.19 *tert*-Butyl *N*^4^-(3-(6-amino-9*H*-purin-9-yl)-4-methoxyphenyl)-l-asparaginate (30)

A mixture of 28 (300 mg, 0.39 mmol) and (NH_4_)_2_S_2_O_8_ (178 mg, 0.78 mmol) in PBS : CH_3_CN (1 : 1) (4.0 mL) was heated in a microwave reactor at 80 °C for 45 min. The crude was dissolved in ethyl acetate (20 mL) and washed with a saturated solution of NaHCO_3_ (10 mL). The organic layer was dried over Na_2_SO_4_, filtered and evaporated to dryness. Then, to a solution of the intermediate obtained (*m*/*z* = 650 (M + H)^+^; 150 mg, 0.32 mmol) in DCM (3.2 mL), piperidine (317 μL, 3.2 mmol) was added. The mixture was stirred at 25 °C for 2 h. The crude was concentrated *in vacuo* and directly purified by flash chromatography, (gradient 0–5% methanol containing 1% of NH_4_OH in dichloromethane) to afford 68 mg (21% yield) of 30 as a white solid. Mp: 139–142 °C. MS (ES, positive mode): *m*/*z* 428 (M + H)^+^. ^1^H NMR (400 MHz, DMSO-*d*_6_) *δ*: 1.38 (s, 9H, (CH_3_)_3_), 2.53–2.63 (m, 2H, CH_2_β), 3.33 (br s, 2H, NH_2_), 3.61 (t, *J* = 5.7 Hz, 1H, Hα), 3.74 (s, 3H, OCH_3_), 7.24 (d, *J* = 9.0 Hz, 1H, Ar), 7.34 (br s, 2H, NH_2_), 7.66 (m, 1H, Ar), 7.76 (d, *J* = 2.3 Hz, 1H, Ar), 8.11 (s, 1H, H_2_/H_8_), 8.24 (s, 1H, H_2_/H_8_), 10.14 (br s, 1H, CONH).^13^C NMR (100 MHz, DMSO-*d*_6_) *δ*: 27.7 (C(C̲H_3_)_3_), 41.4 (CH_2_β), 52.0 (Cα), 56.1 (OCH_3_), 80.1 (OC̲(CH_3_)_3_), 112.9, 118.2, 119.1, 120.5, 122.6, 132.4, 141.4, 149.5, 150.0, 153.0, 156.2 (Ar), 168.8 (CO), 174.0 (CO). HRMS (ESI): calcd for C_20_H_25_N_7_O_4_ 427.1968, found 427.1970.

#### 4.1.20 *N* ^4^-(3-(6-Amino-9*H*-purin-9-yl)phenyl)-l-asparagine (31)

To a cooled mixture of 29 (10 mg, 0.03 mmol) in DCM (0.25 mL), trifluoroacetic acid (96 μL, 1.3 mmol) was added. The resulting mixture was stirred at 25 °C for 6 h. Volatiles were removed and the solid obtained was lyophilized to yield 10 mg of 31 as a white solid, isolated as TFA salt (73% yield). MS (ES, positive mode): *m*/*z* 342 (M + H)^+^. ^1^H NMR (400 MHz, DMSO-*d*_6_) *δ*: 3.02 (t, *J* = 5.1 Hz, 2H, CH_2_β), 4.30 (s, 1H, Hα), 7.45–7.56 (m, 4H, Ar, NH_2_), 7.67 (dt, *J* = 7.7, 1.8 Hz, 1H, Ar), 8.16 (t, *J* = 2.0 Hz, 1H, Ar), 8.22 (s, 1H, H_2_/H_8_), 8.27 (br s, 2H, NH_2_), 8.53 (s, 1H, H_2_/H_8_), 10.55 (br s, 1H, CONH). ^13^C NMR (100 MHz, DMSO-*d*_6_) *δ*: 35.9 (CH_2_β), 48.5 (Cα), 114.0, 118.1, 118.2, 119.2, 130.0, 135.4, 140.6, 140.7, 149.0, 152.9, 156.1 (Ar),157.8 (q, *J*_C–F_ = 30.6 Hz, C̲OCF_3_-TFA) 167.6 (CO), 170.3 (CO). HRMS (ESI): calcd for C_15_H_15_N_7_O_3_ 341.1236, found 341.1231.

#### 4.1.21 *N* ^4^-(3-(6-Amino-9*H*-purin-9-yl)-4-methoxyphenyl)-l-asparagine (32)

A solution of 30 (60 mg, 0.14 mmol) and TFA (161 μL, 2.11 mmol) in DCM (1.4 mL) was stirred at 25 °C for 48 h. Then, volatiles were removed. The residue obtained was treated with dichloromethane and concentrated to dryness. This procedure was repeated several times. The solid obtained was triturated in Et_2_O, then purified by flash chromatography (C18), (gradient 0–20% acetonitrile in water), obtaining 32 mg (62% yield) of 32 as a white solid. Mp: 199–202 °C. MS (ES, positive mode): *m*/*z* 372 (M + H)^+^. ^1^H NMR (400 MHz, DMSO-*d*_6_) *δ*: 2.71 (dd, *J* = 7.4, 16.4 Hz, 1H, CH_2_β), 2.99 (dd, *J* = 4.5, 16.4 Hz, 1H, CH_2_β), 3.67–3.84 (m, 4H, OCH_3_, Hα) 7.23 (d, *J* = 9.1 Hz, 1H, Ar), 7.33 (br s, 2H, NH_2_), 7.65 (dd, *J* = 2.4, 9.0 Hz, 1H, Ar), 7.81 (d, *J* = 2.5 Hz, 1H, Ar), 8.12 (s, 1H, H_2_/H_8_), 8.23 (s, 1H, H_2_/H_8_), 10.64 (br s, 1H, CONH). ^13^C NMR (100 MHz, DMSO-*d*_6_) *δ*: 37.1 (CH_2_), 50.2 (Cα), 56.2 (OCH_3_), 112.9, 118.2, 119.2, 120.7, 122.6, 132.3, 141.4, 149.6, 150.0, 153.1, 156.2 (Ar), 168.5, 169.8 (CO). HRMS (ESI): calcd for C_16_H_17_N_7_O_4_ 371.1342, found 371.1344.

### 4.2 Enzymatic assays

#### 4.2.1 DENV and ZIKV MTase production and purification

Proteins were produced and purified as previously described.^13^ Briefly, the coding sequence of ZIKV NS5 (aa 4-903) and the ZIKV NS5 MTase (aa 4-278) domain were synthesized (Genscript) based on the sequence of the ZIKV strain H/PF/2013 (GenBank accession no. KJ776791.2) and then cloned into a pQE30 (Qiagen) plasmid with an N-terminal His_6_ tag. The MTase domains (aa 4-278 and aa 1-264) were produced in *Escherichia coli* T7 Express Iq (New England BioLabs). Cells were grown in Terrific Broth at 37 °C until the optical density at 600 nm (OD_600_) reached 0.6. Protein expression was then induced by 0.5 mM IPTG (isopropyl-β-d-thiogalactopyranoside) at 17 °C overnight. Bacteria were harvested by centrifugation. The bacterial pellets from a 2-liter bacterial culture were resuspended in 100 ml lysis buffer (50 mM Tris–HCl [pH 8], 300 mM NaCl, 5% glycerol, 0.1% Triton, 10 μg per ml DNase I, 2 tablets of EDTA-free antiprotease cocktail [Roche], 0.25 mg per ml lysozyme). After 30 min of incubation at 4 °C, cells were sonicated and clarified by centrifugation prior to immobilized metal affinity chromatography (IMAC) purification on a 5-mL His prep column (GE Healthcare), with elution in 50 mM Tris–HCl, 300 mM NaCl, and 250 mM imidazole (pH 8.0). The eluted protein was then loaded on a 16/60 Superdex 200 (GE Healthcare) equilibrated in a mixture of 10 mM HEPES, 500 mM NaCl, glycerol 5%, and 1 mM dithiothreitol (DTT [pH 7.5]).

#### 4.2.2 Filter binding assay (FBA) for MTase activity determination

The FBA MTase assay was carried out in a reaction mixture [40 mM Tris–HCl (pH 8.0), 1 mM DTT, 1.9 μM SAM, and 0.1 μM ^3^H-SAM (PerkinElmer)] in the presence of 0.7 μM GpppAC_4_ synthetic RNA and the MTase (500 nM), according to the method previously described.^39^ Briefly, the MTase was first mixed with the compound suspended in DMSO (5% final DMSO) before the addition of RNA substrate and SAM and then incubated at 30 °C. Reactions mixtures were stopped after 30 min by their 10-fold dilution in ice-cold water. Samples were transferred to diethylaminoethyl (DEAE) filtermat (PerkinElmer) using a Filtermat Harvester (Packard Instruments). The RNA-retaining mats were washed twice with 10 mM ammonium formate pH 8.0, twice with water and once with ethanol. They were soaked with scintillation fluid (PerkinElmer), and ^3^H-methyl transfer to the RNA substrates was determined using a Wallac MicroBeta TriLux liquid scintillation counter (PerkinElmer).

### 4.3 Computational studies

The 3D structures of compounds 18, 29 and 31 were generated using Maestro,^40^ energy minimizations were carried out using Macromodel,^41^ and pKa of all protonable groups were calculated with Epik,^42^ implemented in the Schordinger Suite. The crystal structure of ZIKV MTase from the Protein Data Bank^43^(PDB ID: 5ULP).

Docking studies were carried out using AutoDock 4.2.^44^ Preparation of MTase and studied compounds pdbqt files were performed with AutoDock Tools 1.5.6. A three-dimensional cubic grid, consisting of 45 × 50 × 45 points with a spacing of 0.375 Å, was defined at the SAM/SAH binding pocket (grid box coordinates: −3.853, +1.019, 26.338). Electrostatic, desolvation, and affinity maps for the atom types present in the studied compounds were calculated using AutoGrid 4.2.6. Docked conformations within the predicted binding cavity were generated using the Lamarckian genetic algorithm in AutoDock 4.2, which explores molecular orientations and torsion angles by randomly altering the molecule's overall position and all rotatable bonds. Default settings were used except for the number of runs, population size, and maximum number of energy evaluations, which were fixed at 250, 100, and 250 000, respectively. Rapid intra- and intermolecular energy evaluations of each configuration was achieved by having the receptor's atomic affinity potentials for aliphatic and aromatic carbon, oxygen, nitrogen, and hydrogen atoms pre-calculated in the three-dimensional grid. A distance-dependent dielectric function was used in the computation of electrostatic interactions. The 250 poses obtained were analysed, clustered, and ranked based on the calculated binding energies given by Autodock.

The best pose of compound 31 was used as a starting point for MD simulations that were carried out using the Amber22 suite of programs.^45^ The ff14SB force field^46^ was used for the protein in combination with the TIP3P water model^47^ and the GAFF2 (ref. 48) for the parametrization of the ligand. The molecular system consisting of compound 27 and ZIKV MTasa was neutralized by the addition of 10 Cl^−^ ions^49^ and immersed in a truncated octahedron of 9.300 TIP3P water molecules. Periodic boundary conditions were applied and electrostatic interactions were treated using the smooth particle mesh Ewald (PME) method^50^ with a grid spacing of 1 Å. The cutoff distance for the non-bonded interactions was 9 Å. The SHAKE algorithm^51^ was applied to all bonds and an integration step of 2.0 fs was used throughout. Solvent molecules and counterions were energy-minimized and allowed to equilibrate around the positionally restrained solute (restrained with a force constant of 5 kcal mol^−1^ Å^−2^) during a 50 ps molecular dynamics simulation conducted at constant temperature (300 K) and pressure (1 atm). These initial harmonic restraints were gradually reduced in a series of progressive energy minimizations until they were completely removed. The resulting systems were heated again from 100 to 300 K during 20 ps and allowed to equilibrate in the absence of any restraints for 10 ns during which system coordinates were collected every 2 ps for further analysis. Three-dimensional complexes structures and trajectory were visually inspected using the computer graphics program PyMOL.^52^ Interatomic distances and root-mean-square deviations (RMSD) from a given structure were monitored using the cpptraj^53^ module in AMBER. Free binding energy and energy contributions per residue between 31 and WNV RdRp were calculated employing the MM_ISMSA method over 500 snapshots taken from the MD trajectory.^34^

### 4.4 Antiviral assays

Assessment of the antiviral activity against ZIKV in VeroE6 cells (African green monkey kidney cells; Vero 76, clone E6; ATCC: Cat# CRL-1586) was done as previously described.^35^ The antiviral assays in the neuroblastoma SH-SY5Y cells (CRL-2266; ATCC) were conducted as follows: SH-SY5Y cells were seeded in assay medium (MEM supplemented with 2% fetal bovine serum, 2 mM l-glutamine and 0.075% sodium bicarbonate; all from Thermo Fisher Scientific) at a density of 15 000 cells/well in 96-well plates. Cells were incubated overnight at 37 °C and 5% CO_2_. The next day, a serial dilution of the compounds was added to the cells, followed by inoculation of the cells with ZIKV MR766 (MOI = 0.01). Following a 7-day incubation at 37 °C and 5% CO_2_, ZIKV-induced cytopathic effect was determined by means of the MTS readout method, analogous to the assay with the VeroE6 cells.^35^ The 50% effective concentration (the compound concentration that is required to inhibit virus-induced CPE by 50%; EC_50_) and the 50% cytotoxic concentration (*i.e.* the compound concentration that is required to inhibit the cell growth by 50%; CC_50_) was determined using logarithmic interpolation.

## Author contributions

Natalia del Río: investigation; Iván Arribas-Álvarez: investigation, validation; José-María Orduña: investigation; Priscila Sutto-Ortiz: investigation; Johan Neyts: supervision, funding acquisition; Suzanne Kaptein: supervision, writing; Etienne Decroly: supervision, funding acquisition; Eva-María Priego: conceptualization, data curation, funding acquisition, writing original draft; María-Jesús Pérez-Pérez: conceptualization, funding acquisition, project administration, supervision, writing original draft, writing – review & editing. All authors have read and agreed to the published version of the manuscript.

## Conflicts of interest

The authors report there are no competing interests to declare.

## Supplementary Material

RA-015-D5RA05362E-s001

## Data Availability

The authors indicate that the data supporting the findings of this study are available within the article and its Supplementary Information (SI). Supplementary information: synthesis of methyl (*S*)-2-((*tert*-butoxycarbonyl)amino)-4-oxobutanoate; Fig. S1, S2, S3 and S4; ^1^H and ^13^C NMR spectra of the most relevant compounds. Docking pdb files ZIKVMTasa-18.pdb, ZIKVMTasa-29.pdb and ZIKVMTasa-31.pdb are available as SI Files. See DOI: https://doi.org/10.1039/d5ra05362e.
