# Supplementary material for: Design, synthesis and evaluation of arylpurine-based sinefungin mimetics as zika virus methyltransferase inhibitors
Source: RSC Adv. 2025 Oct 7;15(44):37309–24. doi: 10.1039/d5ra05362e (PMC12501849; doi:10.1039/d5ra05362e)

Supplementary Information (SI) for RSC Advances.
This journal is © The Royal Society of Chemistry 2025

*Supplementary information related to the article*

# Design, Synthesis and Evaluation of Arylpurine-Based Sinefungin Mimetics as Zika Virus Methyltransferase Inhibitors

Natalia del Río [a,b], Iván Arribas-Álvarez [a], José-María Orduña [a], Priscila Sutto-Ortiz [c], Johan Neyts [d], Suzanne Kaptein [d], Etienne Decroly [c], Eva-María Priego [a*] and María-Jesús Pérez-Pérez [a*]

[a]Instituto de Química Médica (IQM, CSIC) Juan de la Cierva 3, 28006 Madrid (Spain).

[b]Escuela de Doctorado, Universidad Autónoma de Madrid (Spain)

[c]Architecture et Fonction des Macromolécules Biologiques (AFMB), Aix-Marseille Univ., CNRS, Faculté des Sciences Campus Luminy, Marseille, France

[d]KU Leuven, Department of Microbiology, Immunology and Transplantation, Rega Institute for Medical Research, Virology, Antiviral Drug & Vaccine Research Group, Leuven, Belgium

Includes:

- Synthesis of methyl (S)-2-((tert-butoxycarbonyl)amino)-4-oxobutanoate S2
- Figure S1 S3
- Figure S2 S4
- Figure S3 S5
- Figure S4 S6
- $^{1}$H and $^{13}$C NMR spectra of the most relevant compounds. S7

**Methyl (*S*)-2-((*tert*-butoxycarbonyl)amino)-4-oxobutanoate**

To a mixture containing methyl (*tert*-butoxycarbonyl)-L-homoserinate (370 mg, 1.60 mmol) and sodium bicarbonate (1.3 g, 16.00 mmol) in DCM (8 mL), Dess-Martin periodinane suspended in DCM was added (1.0 g, 2.4 mmol, 0.3 M in DCM). The mixture was stirred at rt for one hour and a solution of 1M sodium thiosulphate was added (2 mL). Then the mixture was vigorously stirred for 5 minutes, and 10 mL of a saturated solution of sodium bicarbonate were added. The aqueous phase was extracted with DCM (3x10 mL). The organic phases were combined and washed with a brine solution (20 mL). The organic extract was evaporated, and the residue obtained was purified by column chromatography (hexane/EtOAc, 4/1), affording 271 mg of an amorphous solid containing the aldehyde. $^{1}$H NMR (400 MHz, DMSO-$d_6$) δ: 1.39 (s, 9H, $(CH_3)_3$), 2.72 (ddd, J = 17.4, 8.2, 1.7 Hz, 1H, $CH_{2a}$), 2.84 (ddd, J = 17.4, 5.4, 1.3 Hz, 1H, $CH_{2b}$), 3.63 (s, 3H, $OCH_3$), 4.50 (m, 1H, Cα), 7.32 (d, J = 7.9 Hz, 1H, NH), 9.59 (s, 1H, CHO).

**Figure S1**. A) HPLC/MS of the reaction mixture after treatment of **7** with neat TFA at 70 °C overnight. B) HPLC/MS of the reaction mixture after treatment of **7** with neat TFA at 70 °C for 90 min. C) Proposed chemical structures of the compounds obtained in the reaction of compound **7** with neat TFA at 70 °C based on the m/z values observed in the HPLC/MS chromatograms.

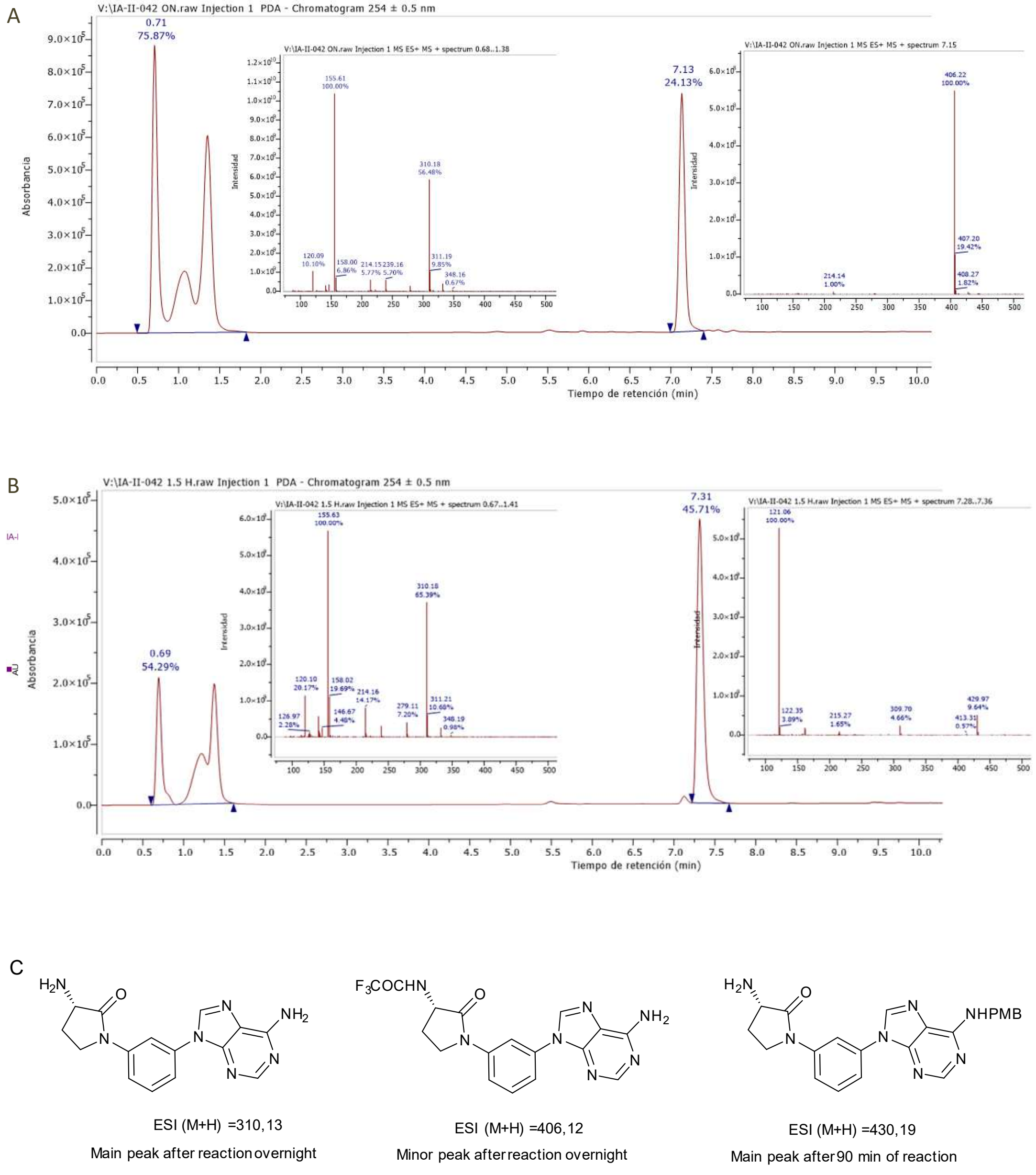

**Figure S2.** (A) Frontal view of the docking pose for compound **31** (orange sticks). ZIKV MTase (PDB: 5ULPD) is shown as grey surface. (B) Lateral detailed view for the docking pose. Space around position 2 of the aromatic ring in **31** is highlighted.

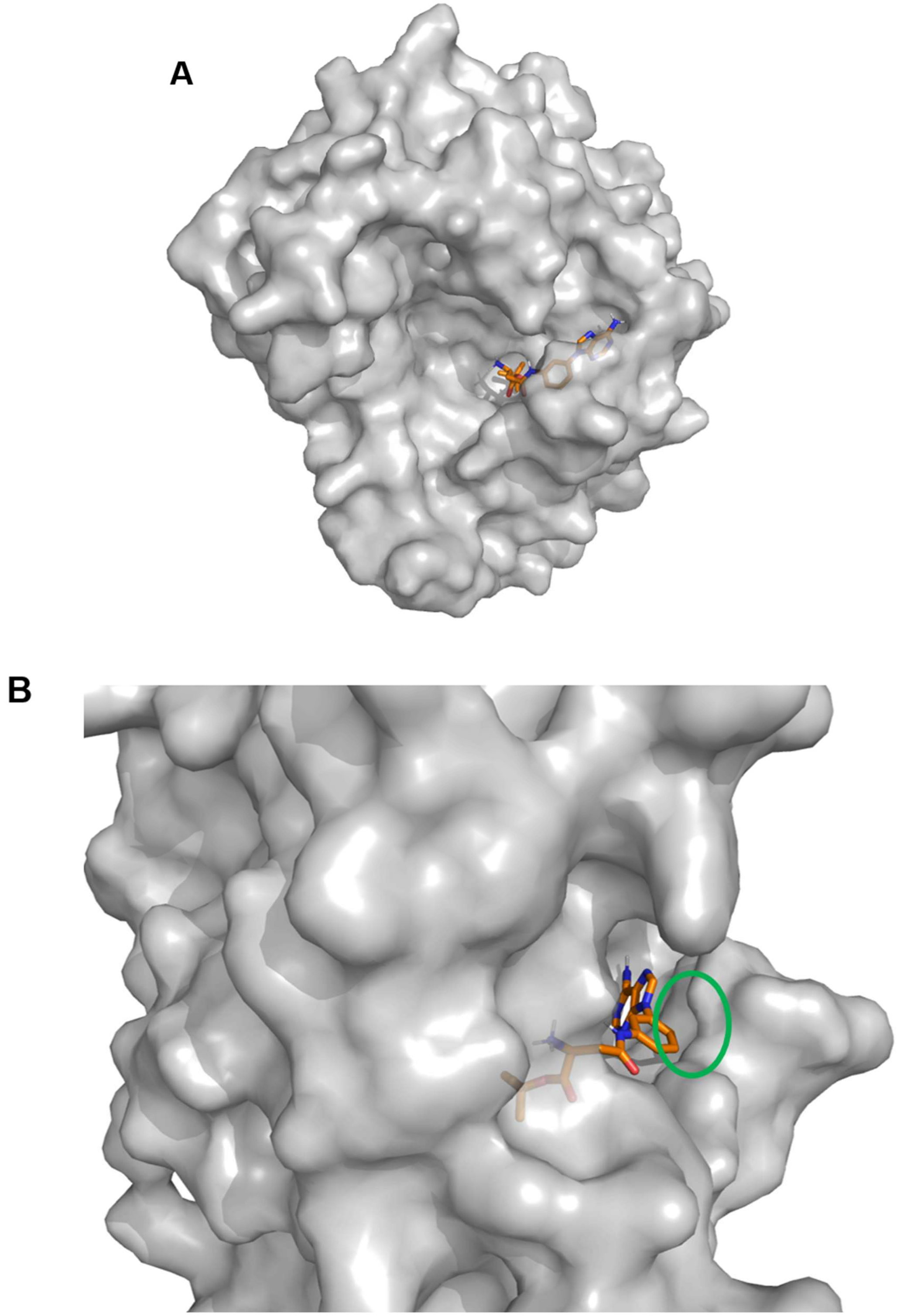

**Figure S3.** A) HPLC/MS of the reaction mixture after treatment of **28** with neat TFA at 70 °C for 1 hour. B) Proposed chemical structures of the compounds obtained in the reaction of compound **28** with neat TFA at 70 °C based on the m/z values observed in the HPLC/MS chromatograms

A

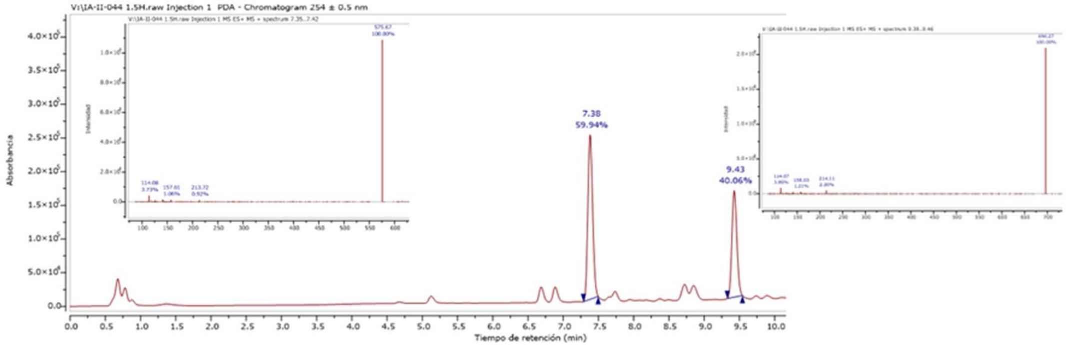


B

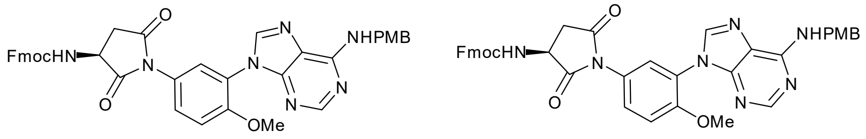


ESI (M+H$^+$) =695,25

ESI (M+H$^+$) =575,19

**Figure S4.** Analysis of MD simulations of ZIKA MTase-**31** complex. (A) RMSD ZIKV MTase (blue) and compound **31** (orange). (B) Distance Asp131- $NH_2$ (blue) and Val132-N (orange) (C) Distance Lys82-COO- (blue) and Gly148-$COO^-$ (orange). (D) Distance Asp146-$NH_3^+$

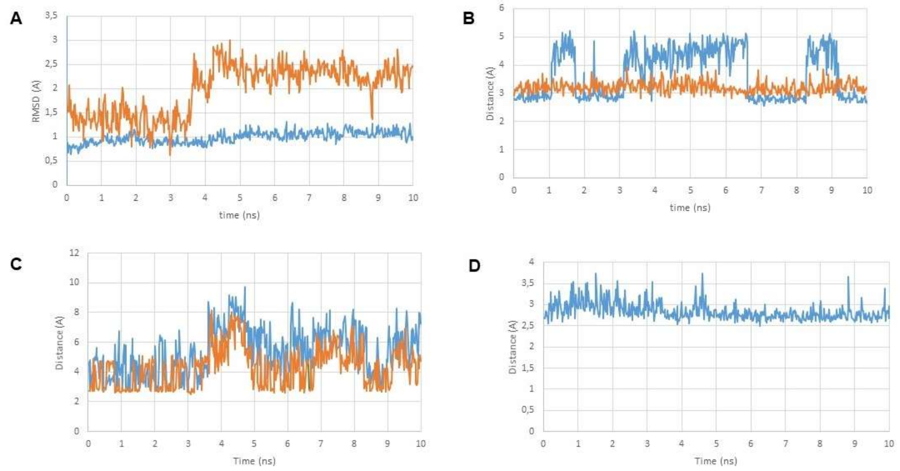

***tert*-Butyl *(S)*-2-((*tert*-butoxycarbonyl)amino)-4-((3-(6-((4-methoxybenzyl)amino)-9*H*-purin-9-yl)phenyl)amino)butanoate (9).**

$^{1}$H NMR (400 MHz, $CDCl_3$)

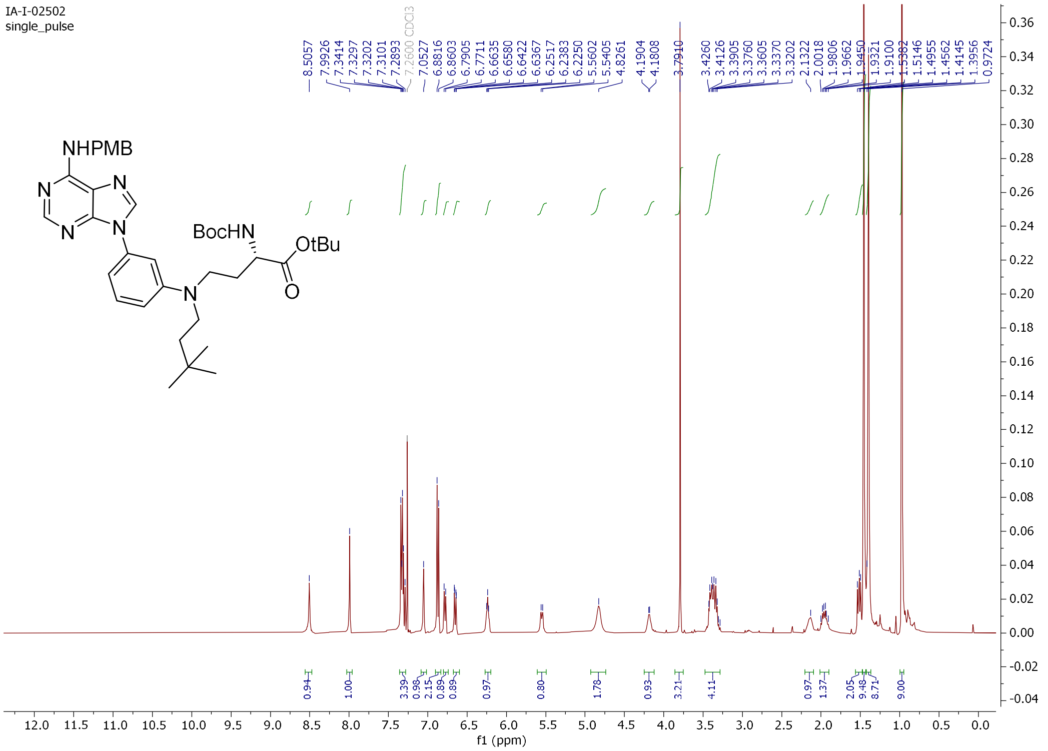


$^{13}$C NMR (100 MHz, $CDCl_3$)

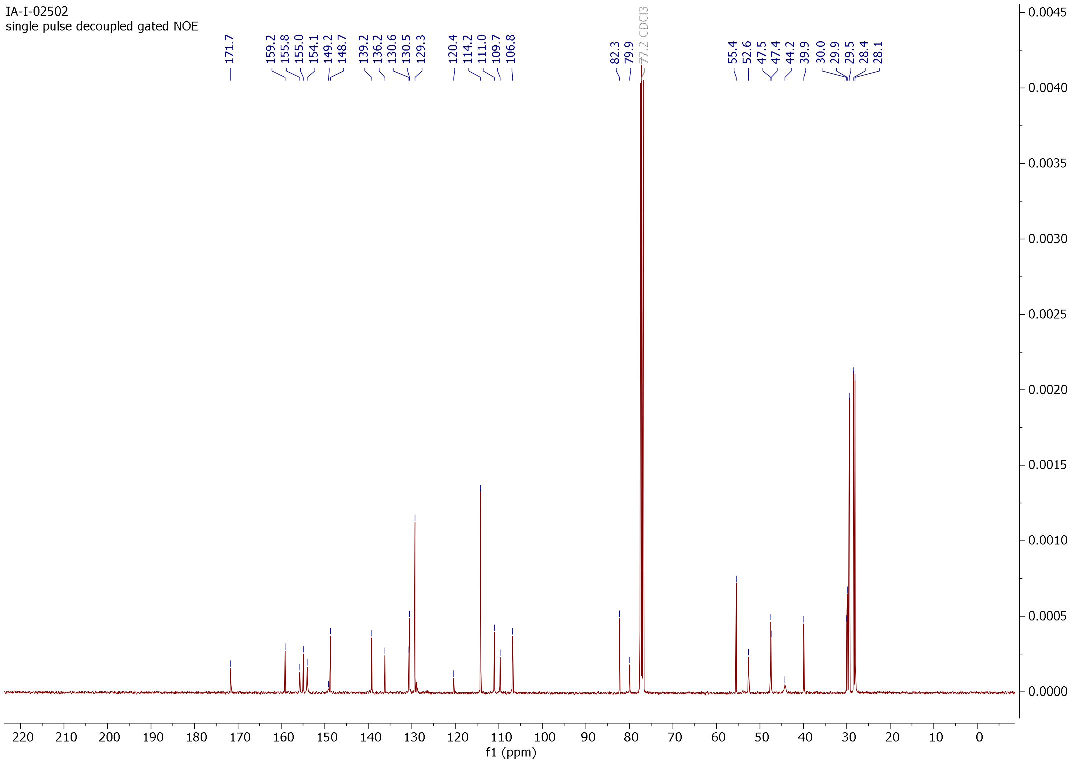

**(*S*)-2-Amino-4-((3-(6-amino-9*H*-purin-9-yl)phenyl)(3,3-dimethylbutyl)amino)butanoic acid (10).**

$^{1}$H NMR (400 MHz, DMSO-$d_6$)

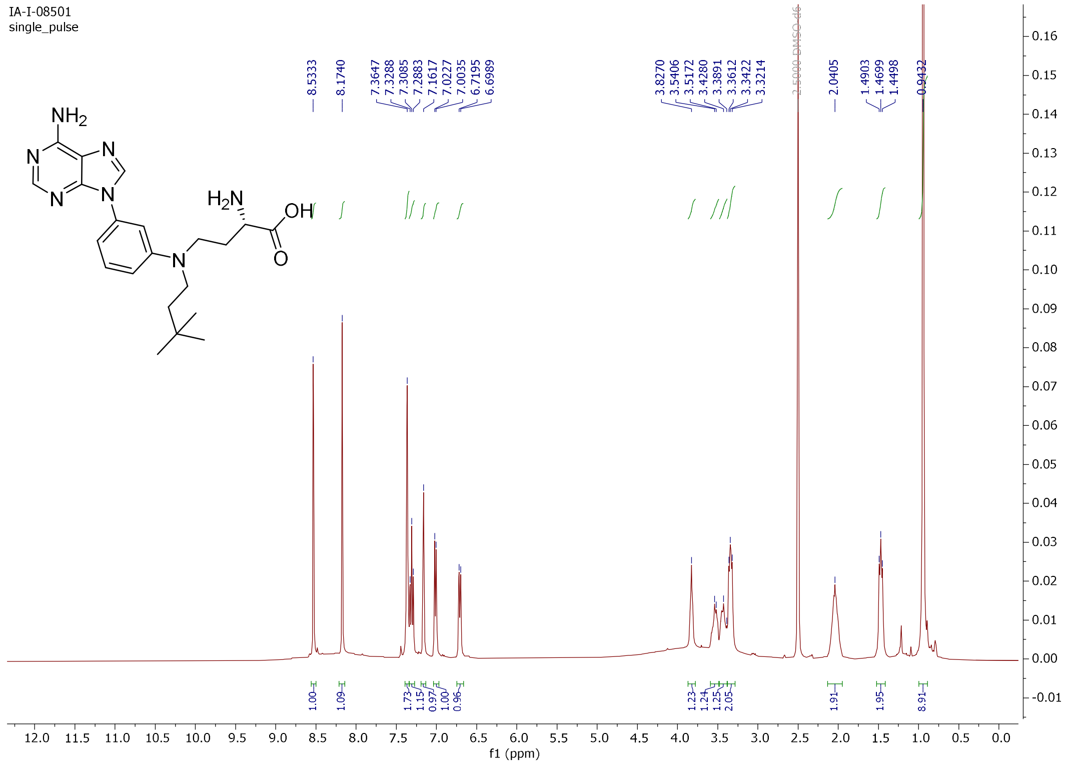


$^{13}$C NMR (100 MHz, DMSO-$d_6$)

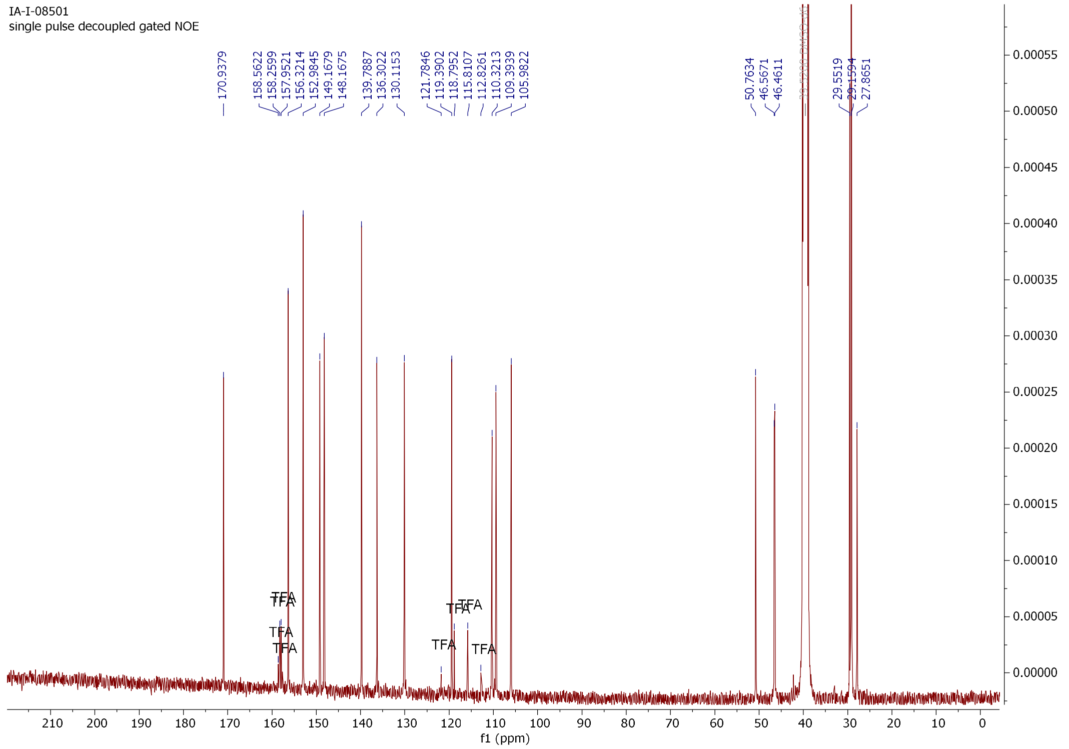

**_tert_-Butyl _(S)_-2-((_tert_-butoxycarbonyl)amino)-4-((3,3-dimethylbutyl)(3-(4-methyl-7_H_-pyrrolo[2,3-_d_]pyrimidin-7-yl)phenyl)amino)butanoate (13).**

$^1$H NMR (400 MHz, $CDCl_3$)

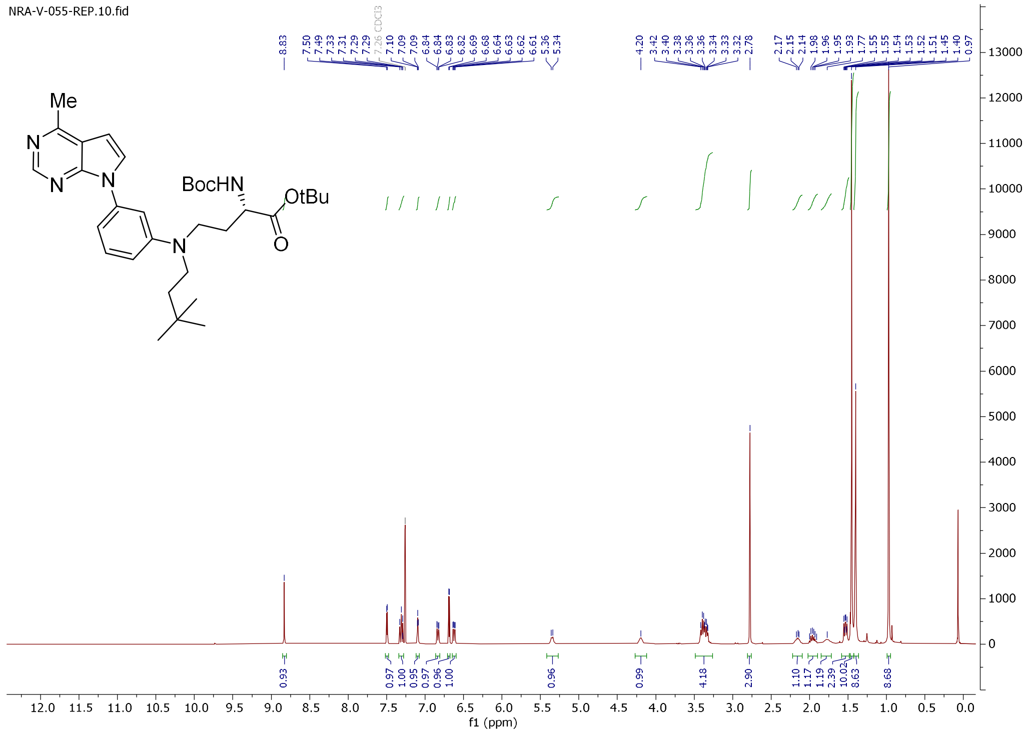


$^{13}$C NMR (100 MHz, $CDCl_3$)

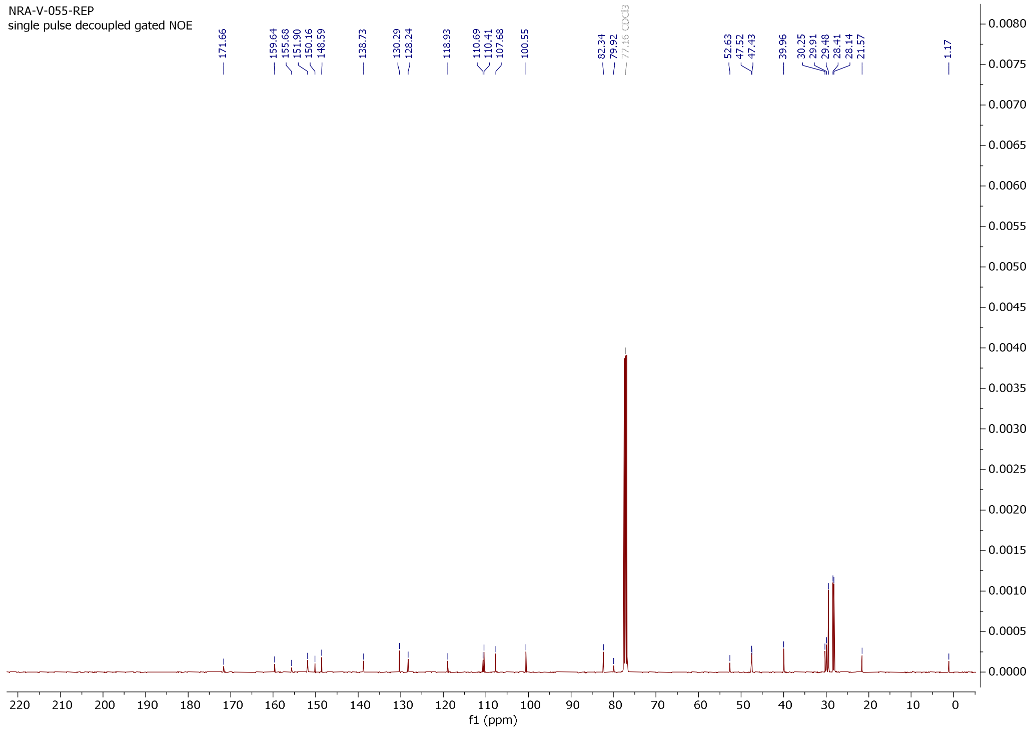

**(*S*)-2-Amino-4-((3,3-dimethylbutyl)(3-(4-methyl-7*H*-pyrrolo[2,3-*d*]pyrimidin-7-yl)phenyl)amino)butanoic acid (14).**

$^{1}$H NMR (400 MHz, DMSO-$d_6$)

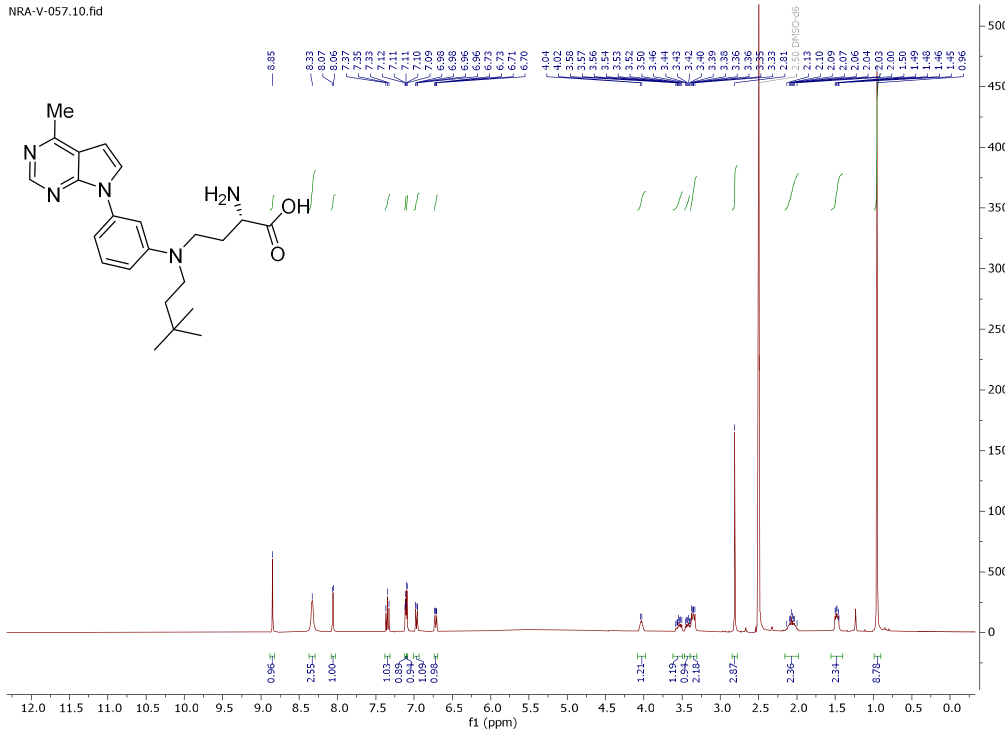


$^{13}$C NMR (100 MHz, DMSO-$d_6$)

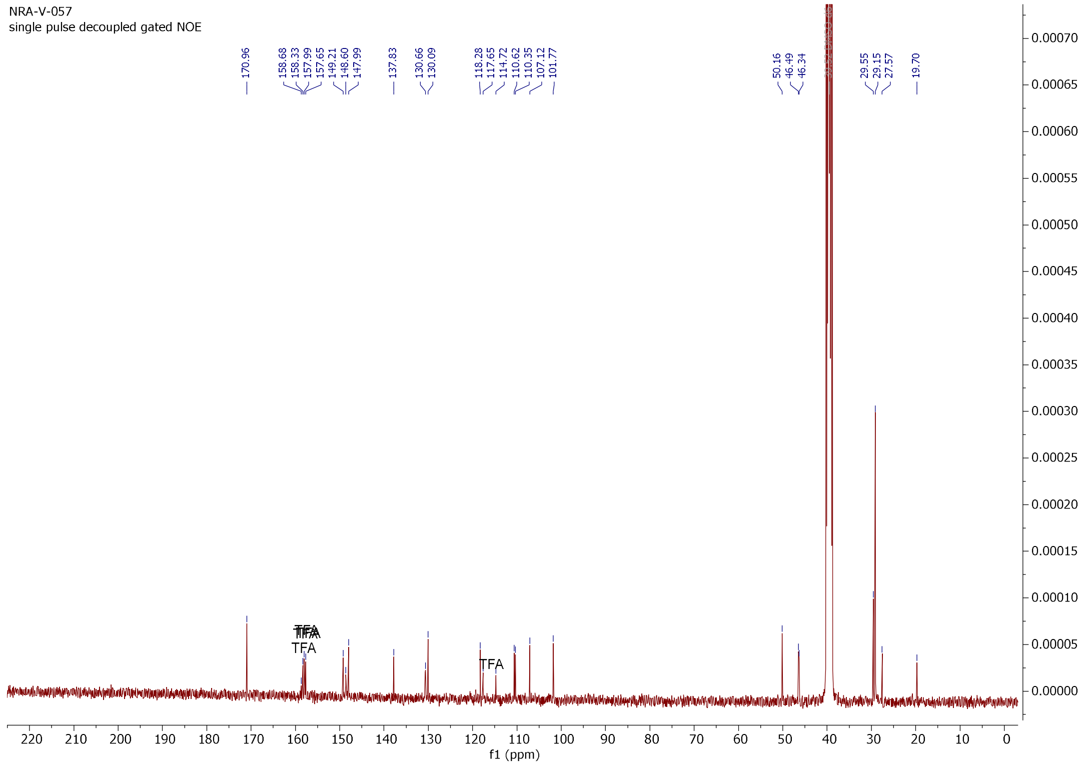

**Methyl *(S)*-2-((tert-butoxycarbonyl)amino)-4-((3-(4-methyl-7*H*-pyrrolo[2,3-*d*]pyrimidin-7-yl)phenyl)amino)butanoate (15).**

$^1$H NMR (400 MHz, CDCl$_3$)

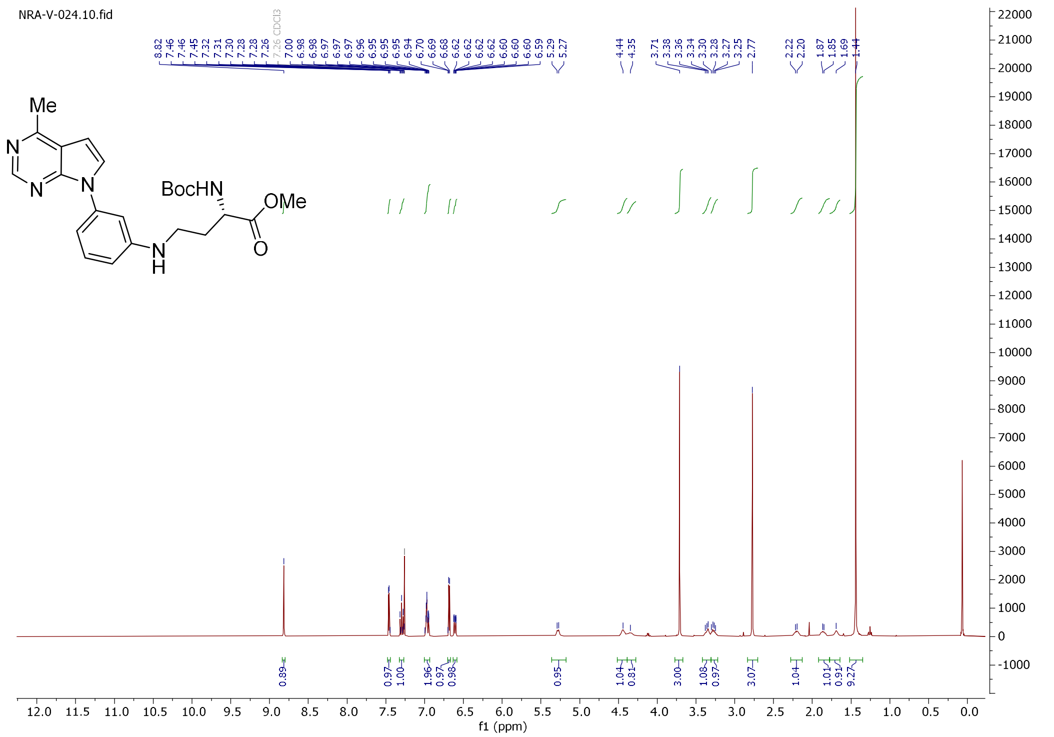


$^{13}$C NMR (100 MHz, CDCl$_3$)

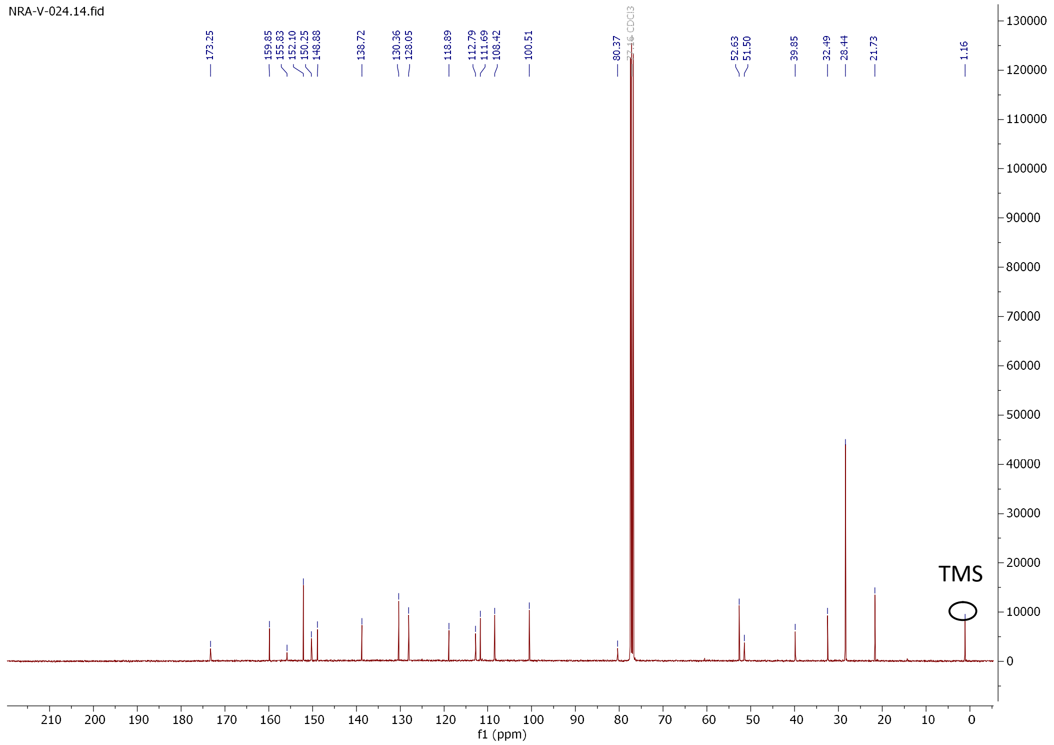

**Methyl *(S)*-2-((*tert*-butoxycarbonyl)amino)-4-((3,3-dimethylbutyl)(3-(4-methyl-7*H*-pyrrolo[2,3-*d*]pyrimidin-7-yl)phenyl)amino)butanoate (16).**

$^{1}$H NMR (400 MHz, $CDCl_3$)

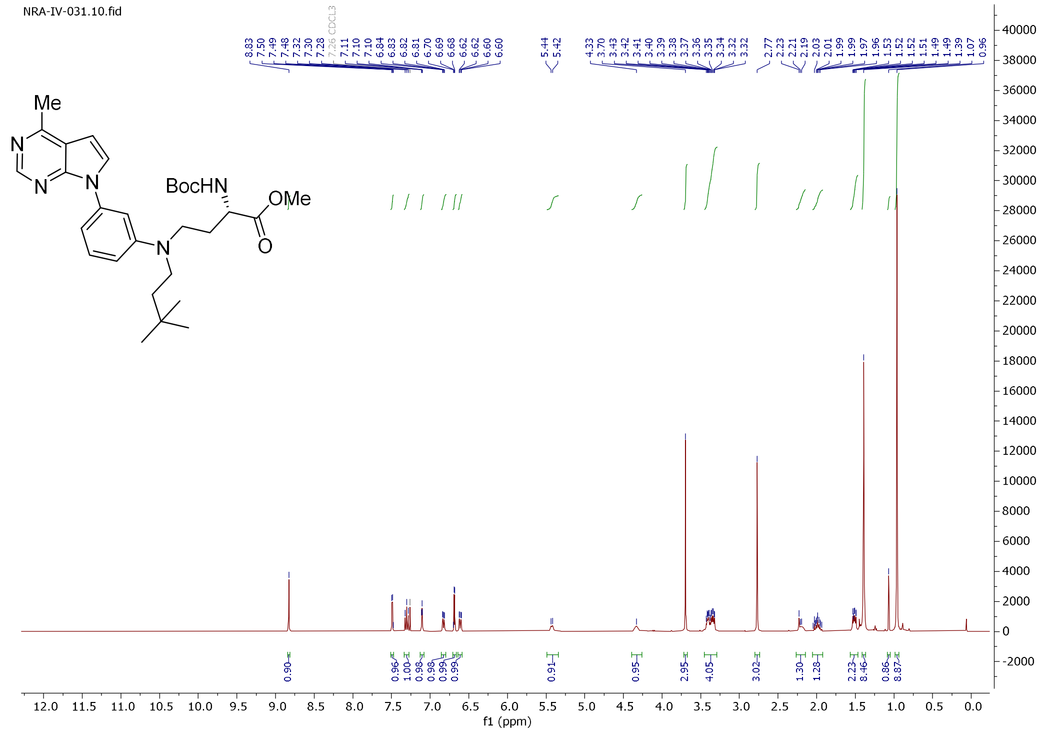


$^{13}$C NMR (100 MHz, $CDCl_3$)

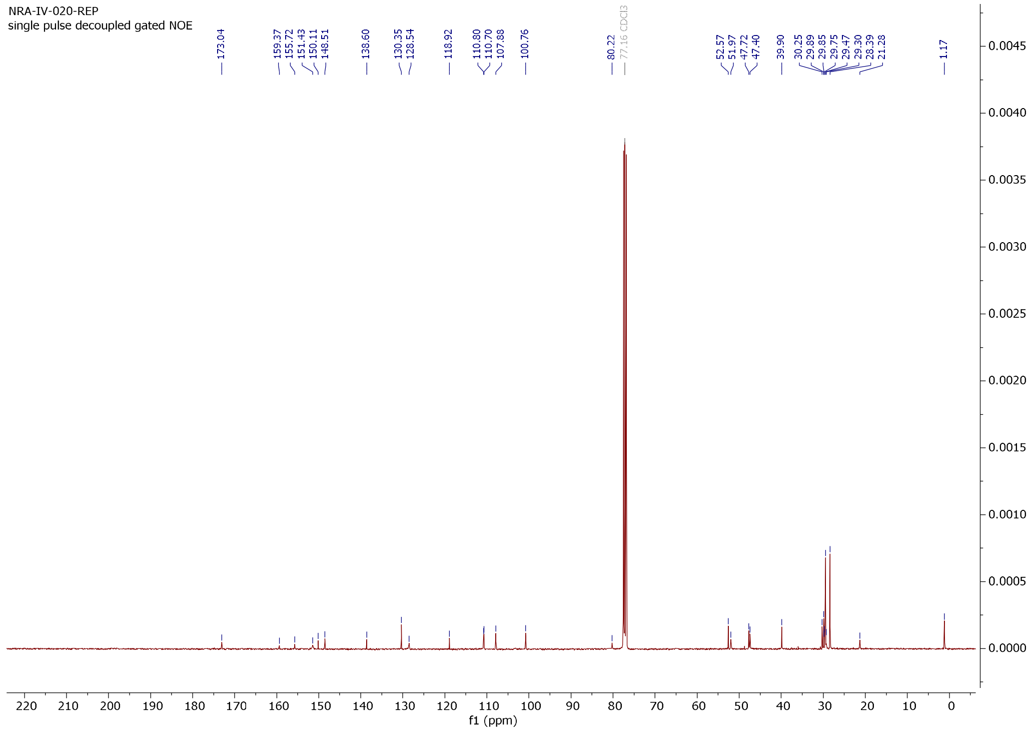

**_tert_-Butyl _(S)_-(1-amino-4-((3,3-dimethylbutyl)(3-(4-methyl-7_H_-pyrrolo[2,3-_d_]pyrimidin-7-yl)phenyl)amino)-1-oxobutan-2-yl)carbamate (17).**

$^1$H NMR (400 MHz, $CDCl_3$)

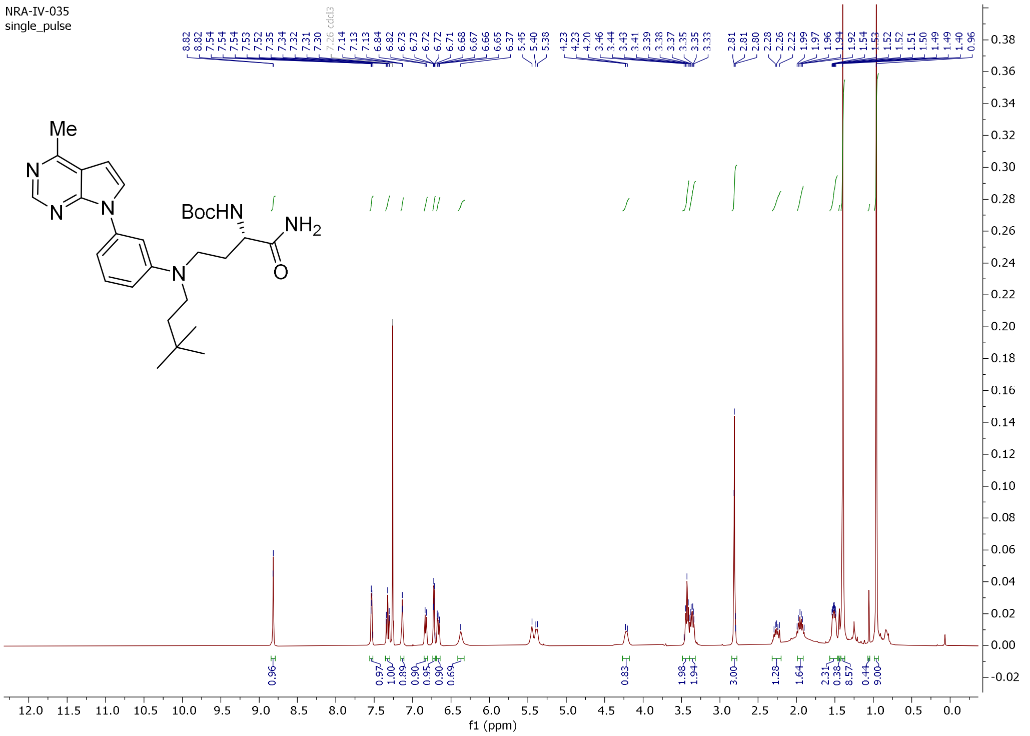


$^{13}$C NMR (100 MHz, $CD_3OD$)

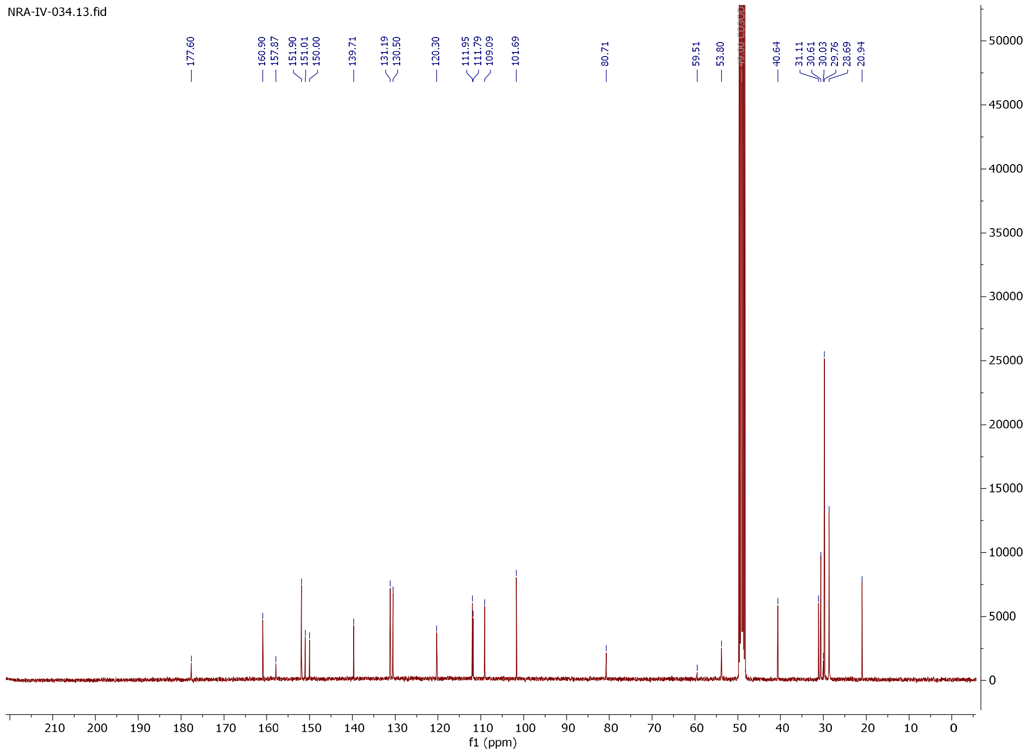

**(S)-2-Amino-4-((3,3-dimethylbutyl)(3-(4-methyl-7*H*-pyrrolo[2,3-*d*]pyrimidin-7-yl)phenyl)amino)butanamide (18).**

$^{1}$H NMR (400 MHz, DMSO-$d_6$ + $D_2O$)

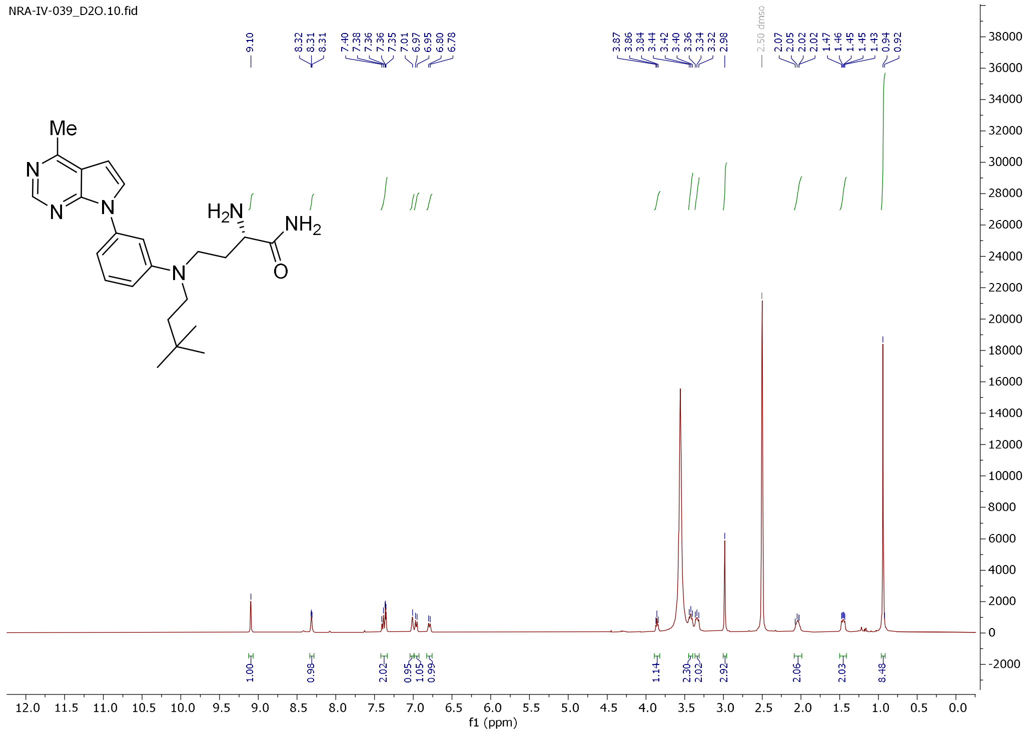


$^{13}$C NMR (100 MHz, DMSO-$d_6$ + $D_2O$)

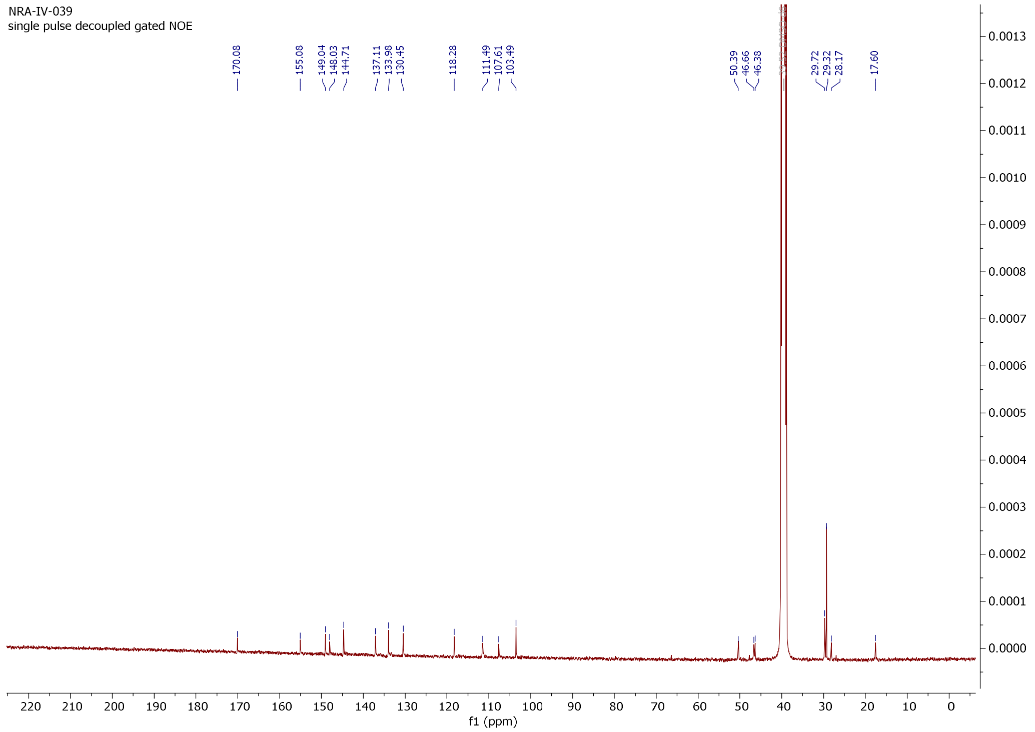

## ***tert*-Butyl *N*[2]-(((9*H*-fluoren-9-yl)methoxy)carbonyl)-*N*[4]-(3-(4-methyl-7*H*-pyrrolo[2,3-*d*]pyrimidin-7-yl)phenyl)-*L*-asparaginate (19).**

$^1$H NMR (400 MHz, $CDCl_3$)

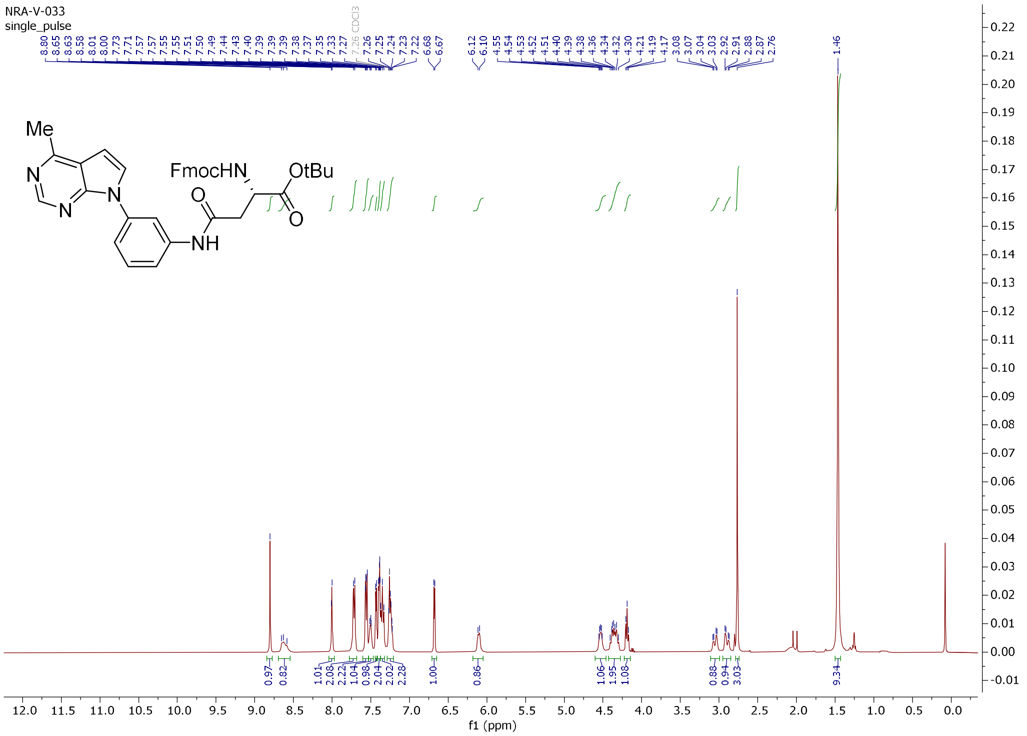


$^{13}$C NMR (100 MHz, $CDCl_3$)

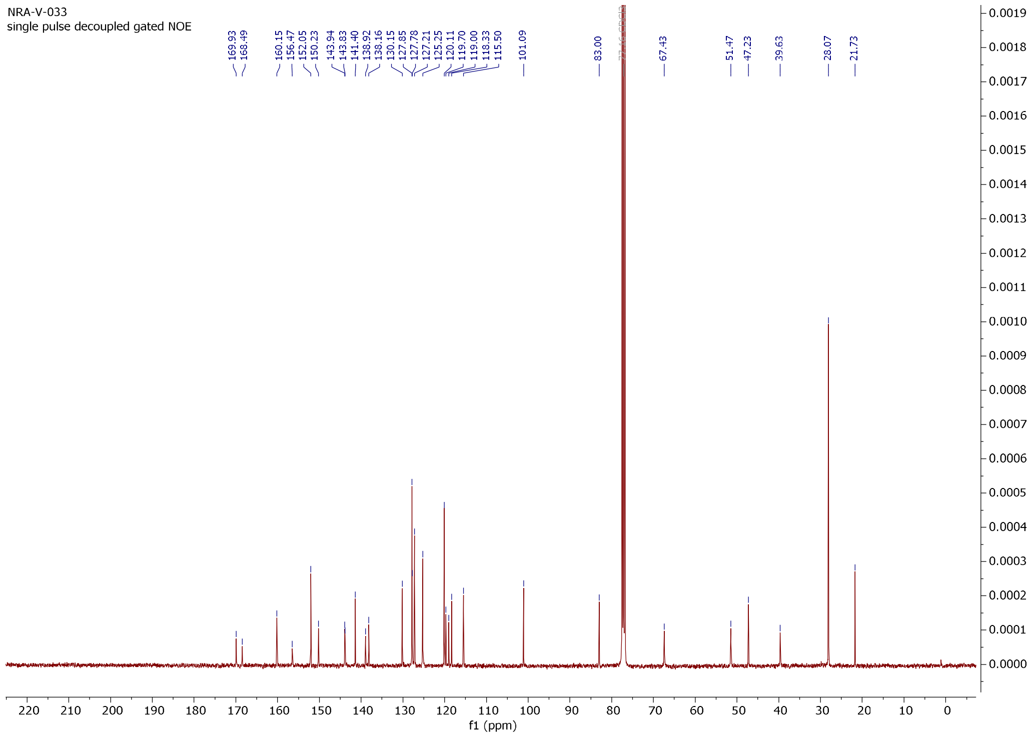

## ***tert*-Butyl *N*[4]-(3-(4-methyl-7*H*-pyrrolo[2,3-*d*]pyrimidin-7-yl)phenyl)-*L*-asparaginate (20).**

$^{1}$H NMR (400 MHz, $CDCl_3$)

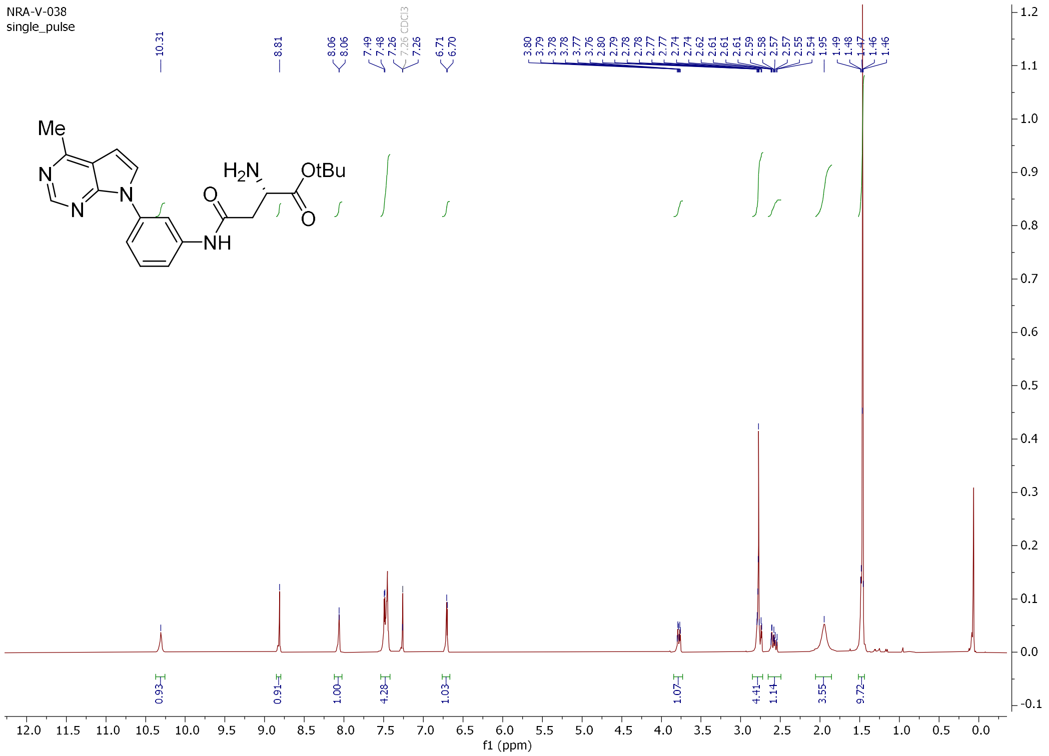


$^{13}$C NMR (100 MHz, $CDCl_3$)

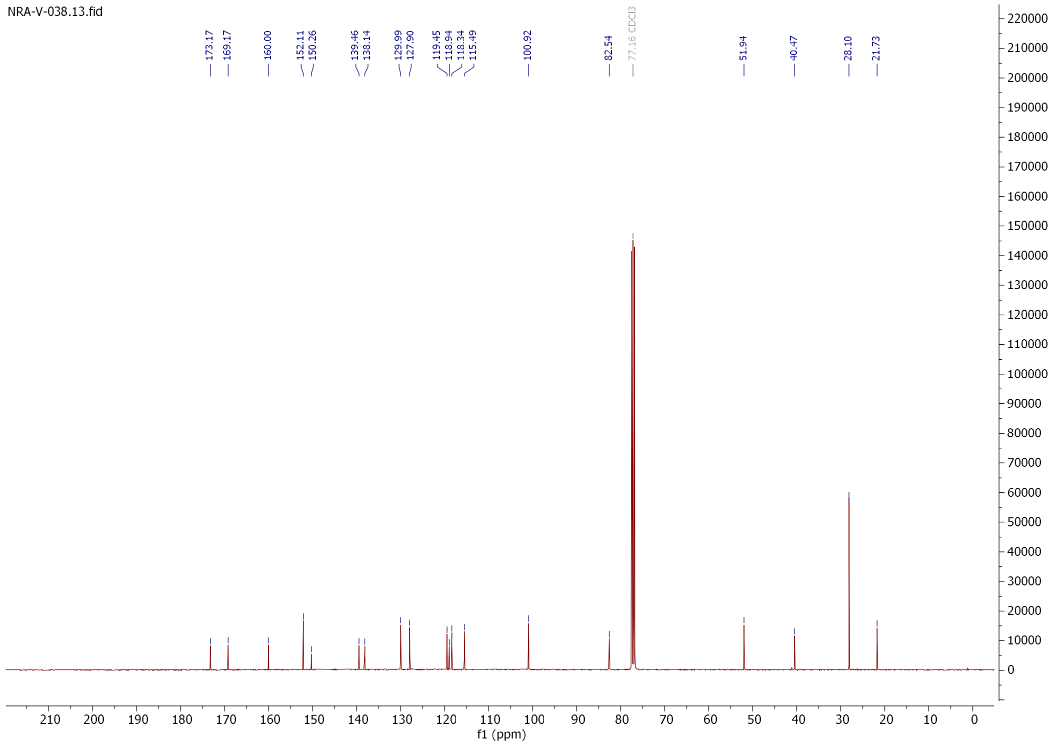

**$N^4$-(3-(4-Methyl-7*H*-pyrrolo[2,3-*d*]pyrimidin-7-yl)phenyl)-*L*-asparagine (21).**

$^1H$ NMR (400 MHz, DMSO-$d_6$)

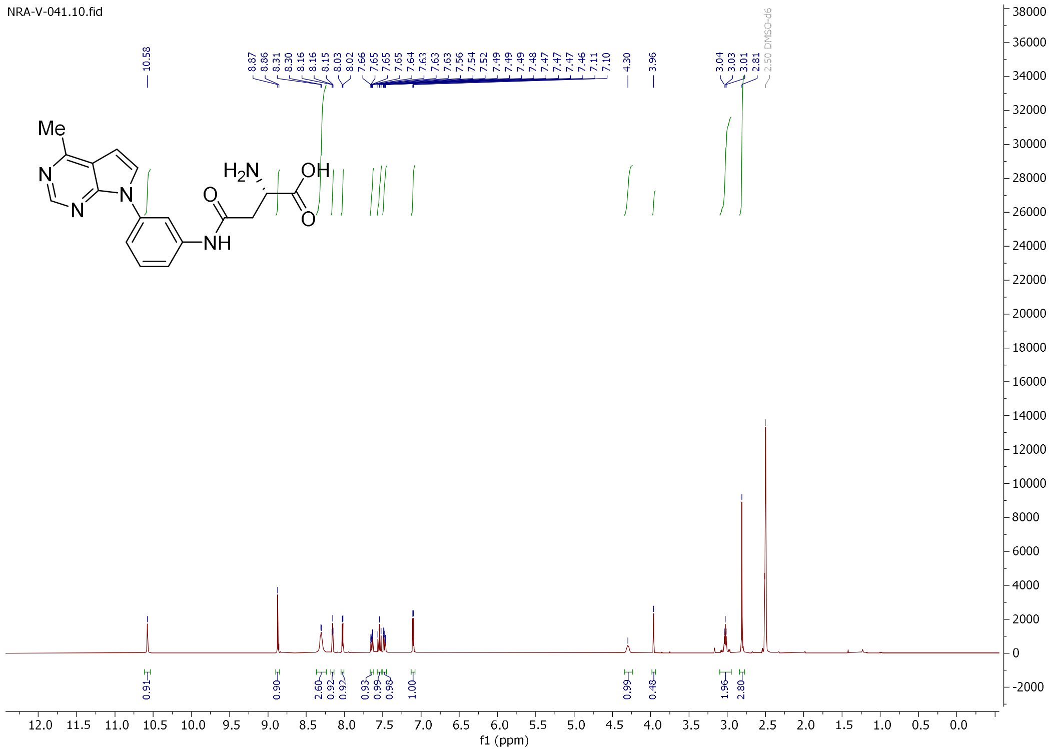


$^{13}C$ NMR (100 MHz, DMSO-$d_6$)

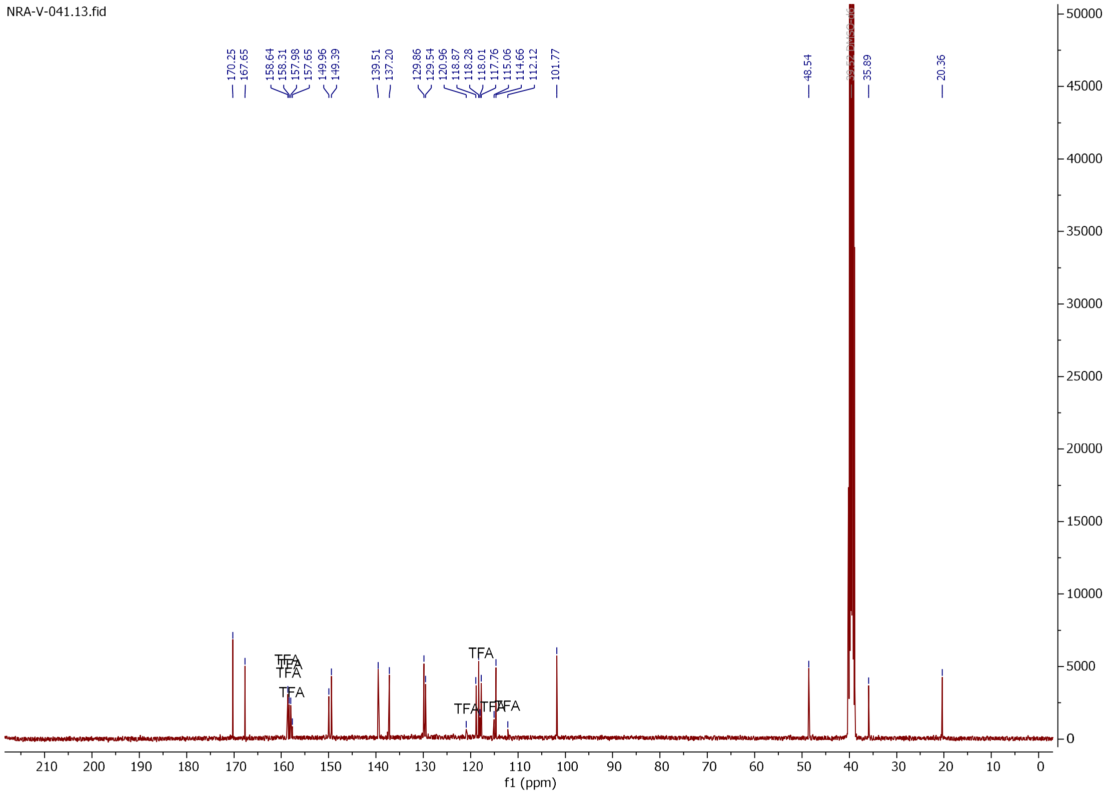

**9-(5-Amino-2-methoxyphenyl)-*N*-(4-methoxybenzyl)-9*H*-purin-6-amine (22).**

$^{1}$H NMR (400 MHz, DMSO-$d_6$)

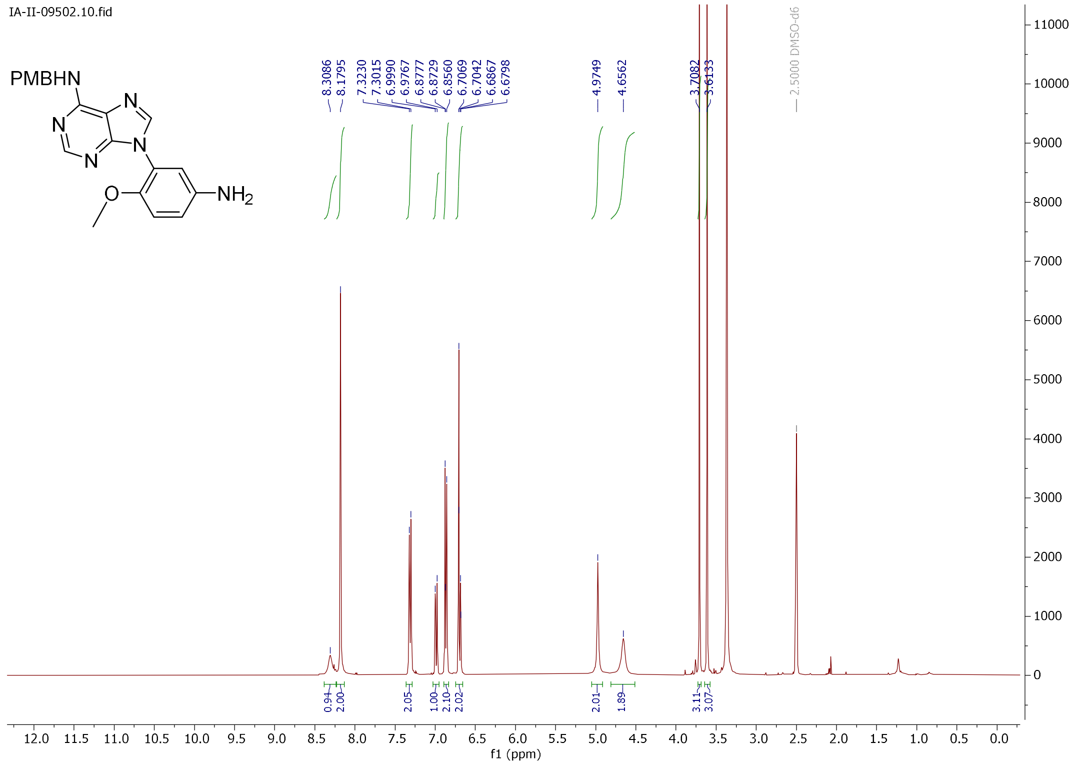

$^{13}$C NMR (100 MHz, DMSO-$d_6$)

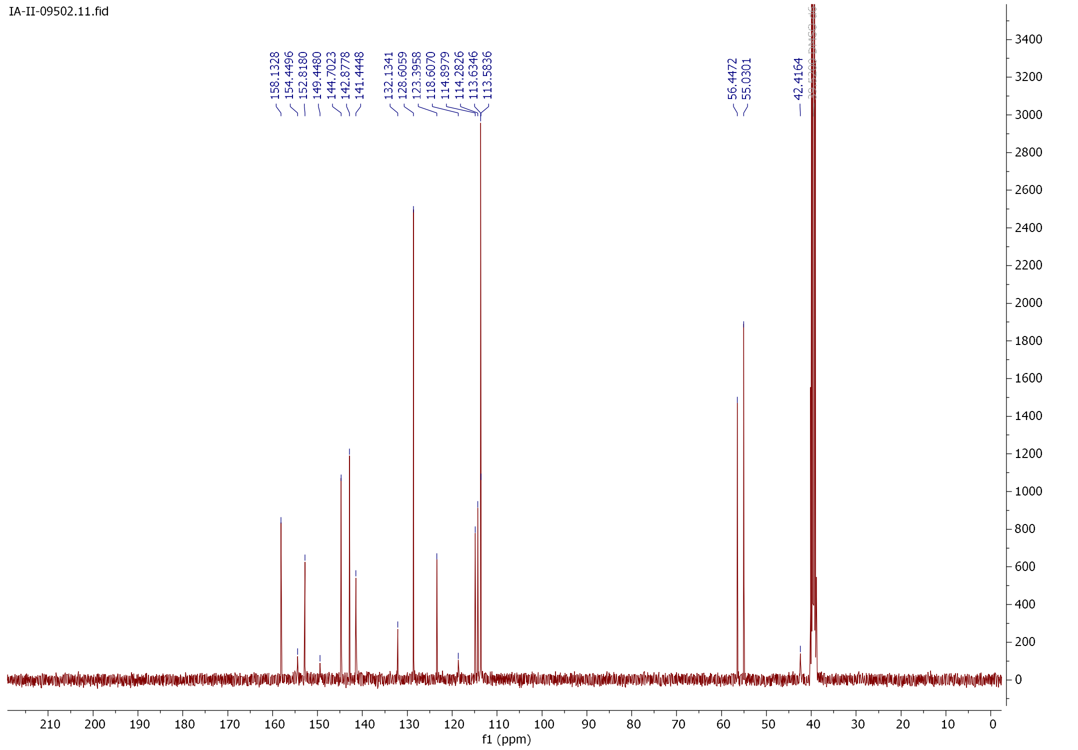

**6-Chloro-$N^4$-(2-methoxy-5-nitrophenyl)pyrimidine-4,5-diamine (24).**

$^{1}H$ NMR (400 MHz, DMSO-$d_6$)

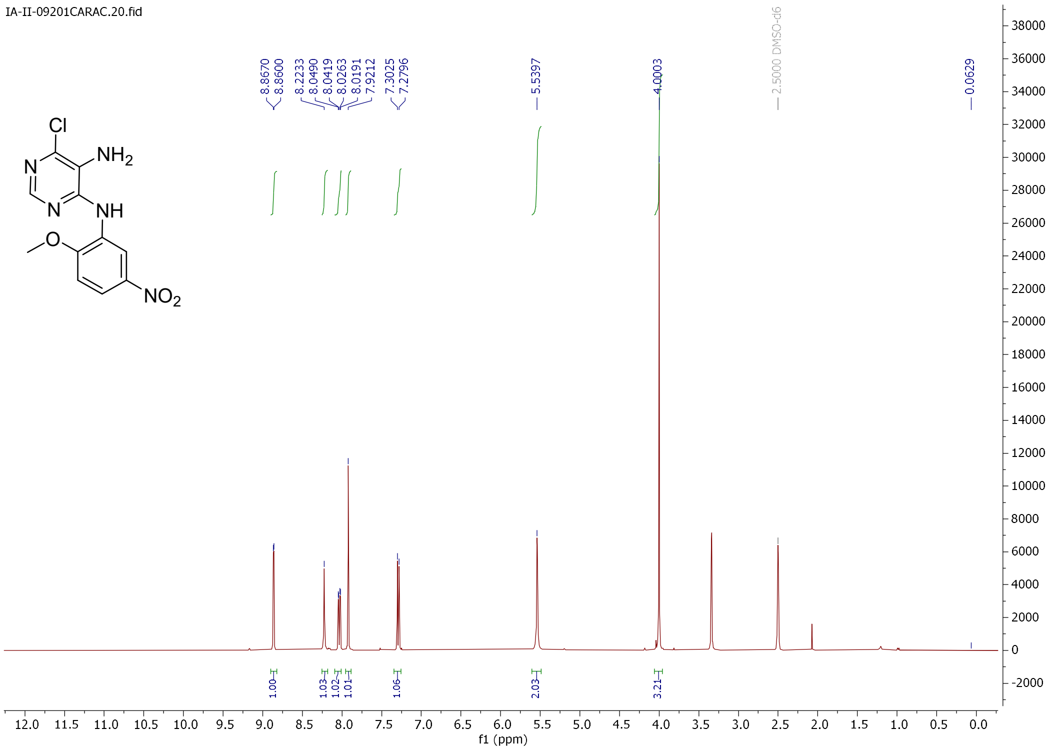


$^{13}C$ NMR (100 MHz, DMSO-$d_6$)

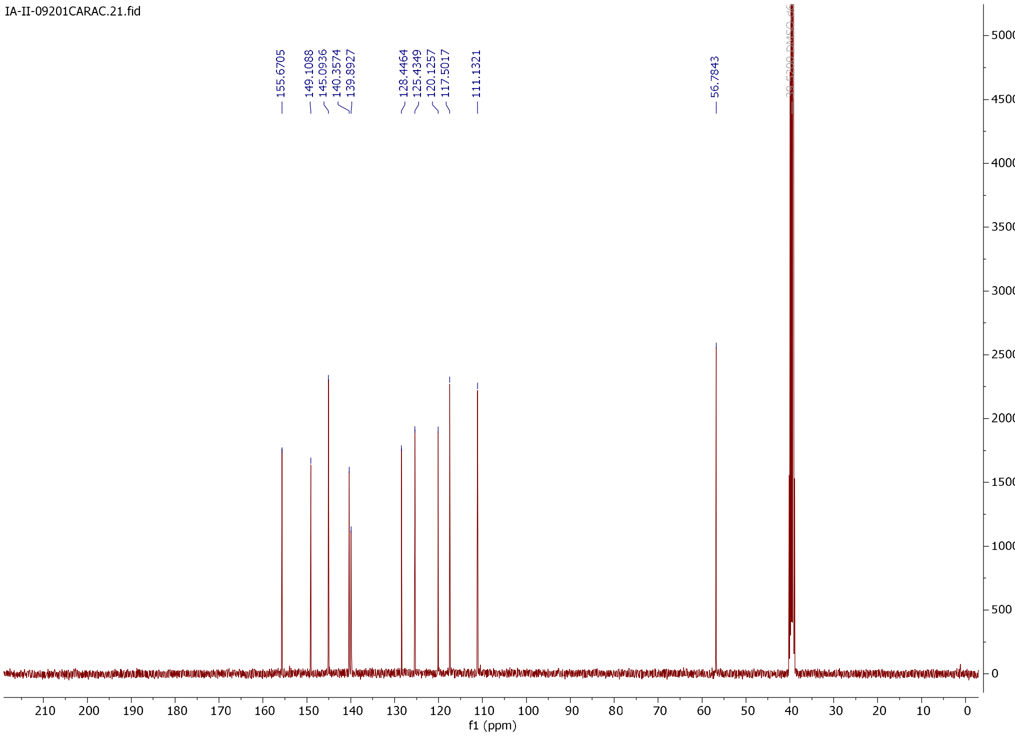

**6-Chloro-9-(2-methoxy-5-nitrophenyl)-9*H*-purine (25).**

$^{1}$H NMR (400 MHz, DMSO-$d_6$)

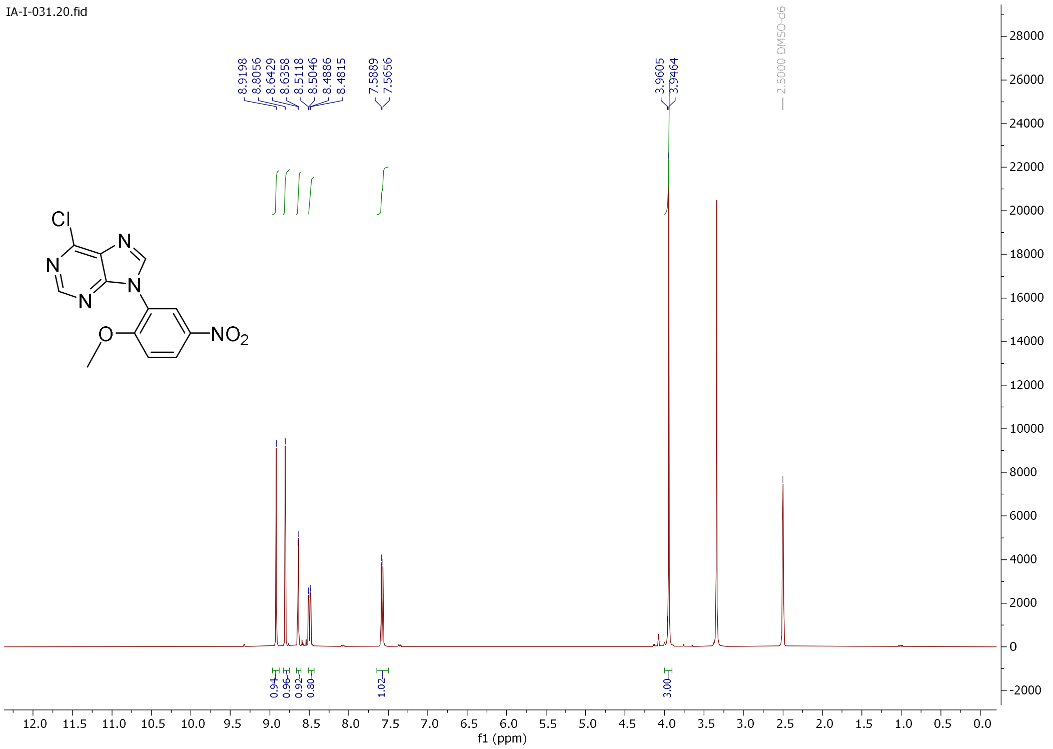


$^{13}$C NMR (100 MHz, DMSO-$d_6$)

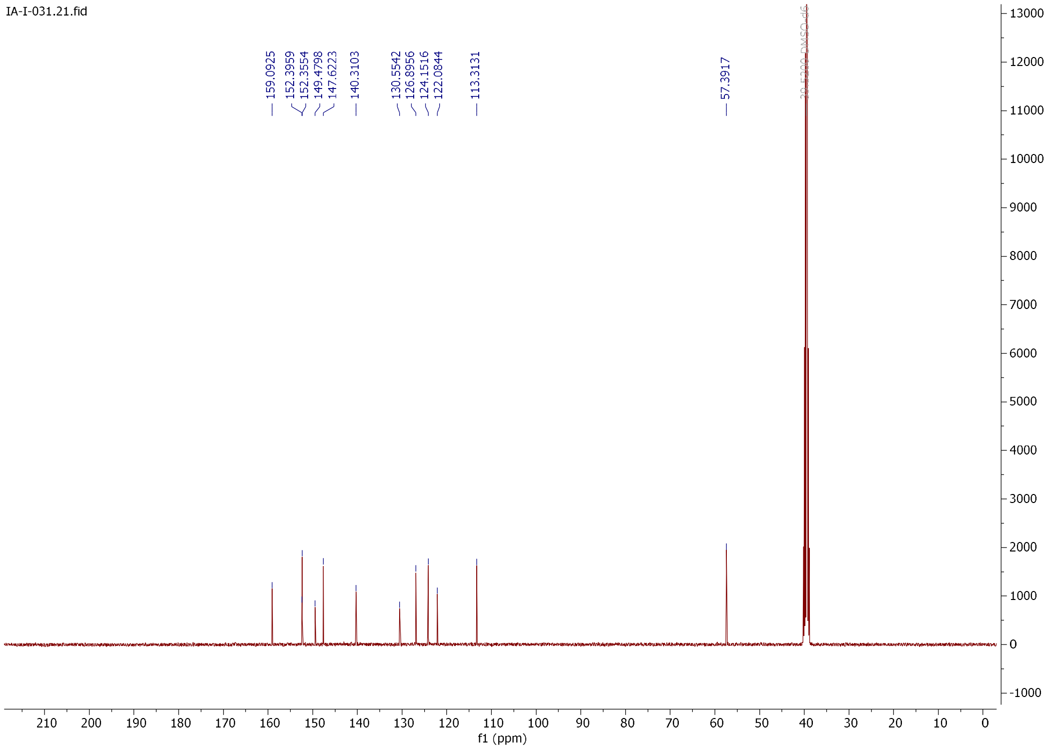

**9-(2-Methoxy-5-nitrophenyl)-*N*-(4-methoxybenzyl)-9*H*-purin-6-amine (26).**

$^{1}$H NMR (400 MHz, DMSO-$d_6$)

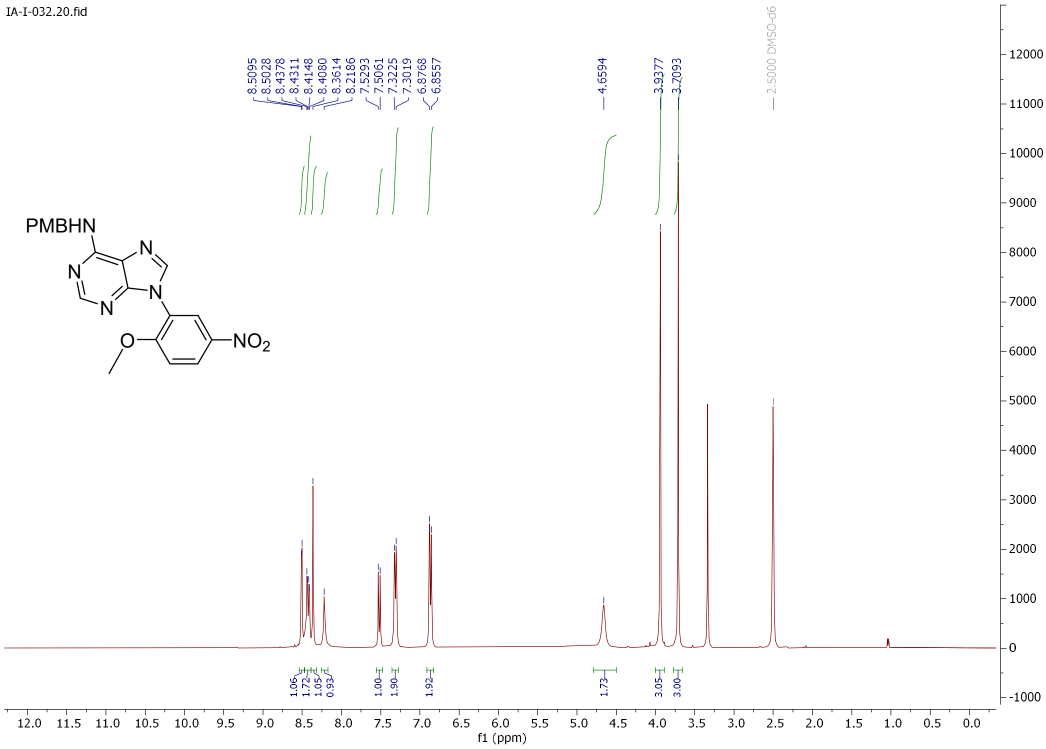


$^{13}$C NMR (100 MHz, DMSO-$d_6$)

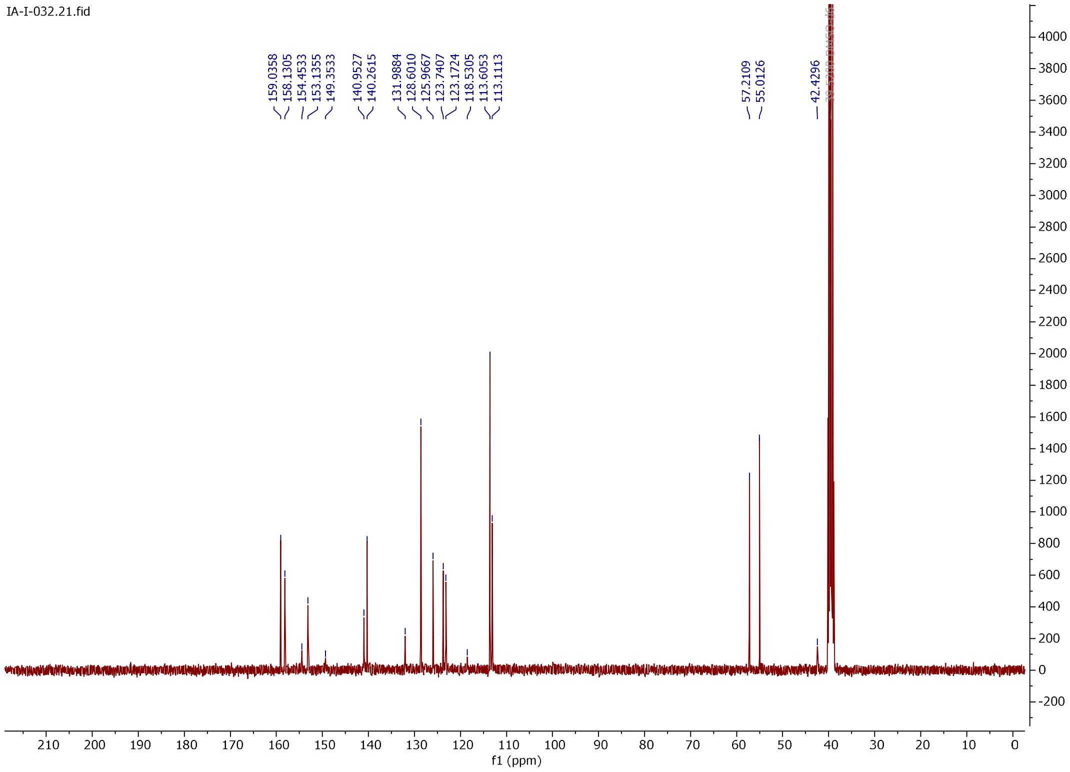

**_tert_-butyl _N_²-(((9_H_-fluoren-9-yl)methoxy)carbonyl)-_N_⁴-(3-(6-((4-methoxybenzyl)amino)-9_H_-purin-9-yl)phenyl)-_L_-asparaginate (27).**

$^{1}H$ NMR (400 MHz, DMSO-$d_6$)

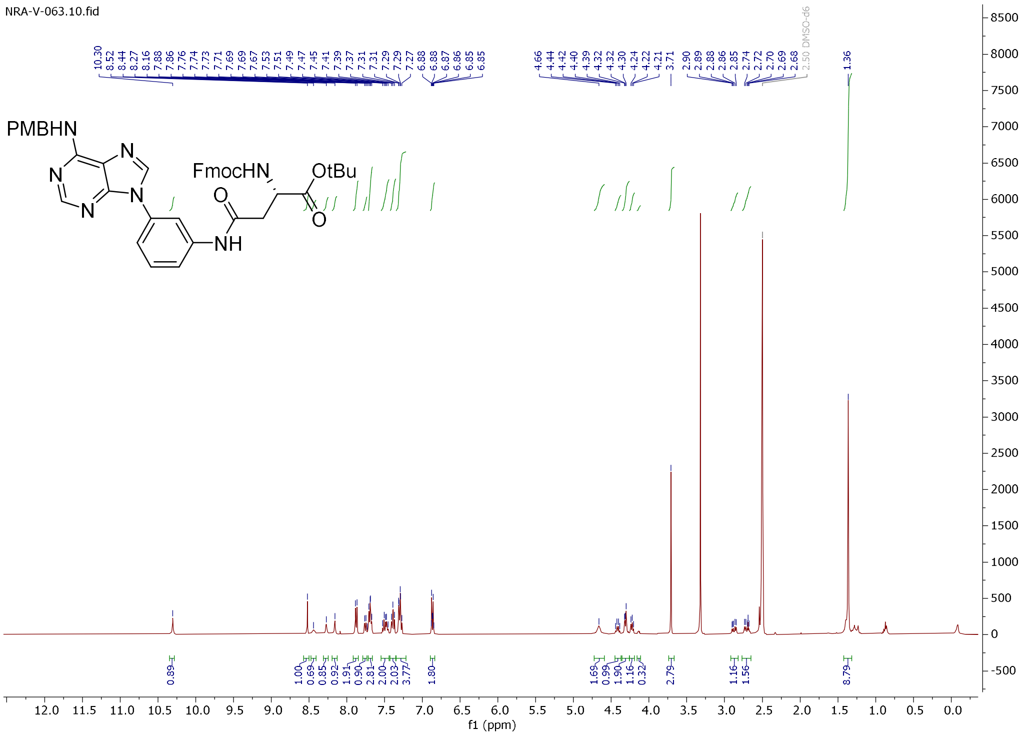


$^{13}C$ NMR (100 MHz, DMSO-$d_6$)

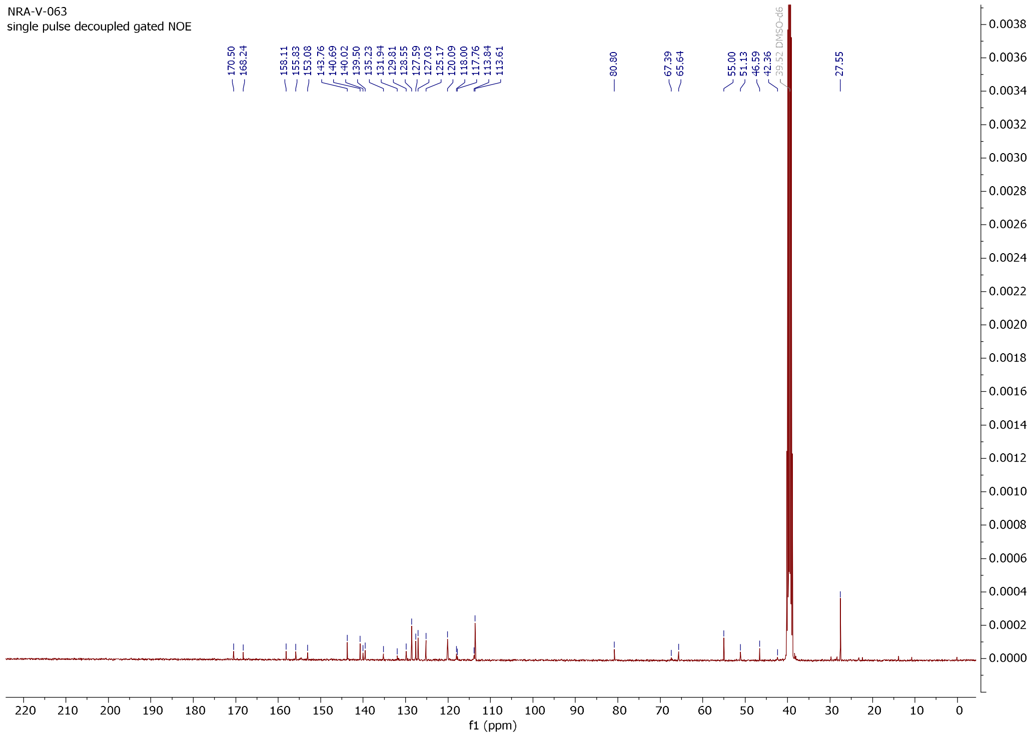

**_tert_-Butyl _N_[2]-(((9_H_-fluoren-9-yl)methoxy)carbonyl)-_N_[4]-(4-methoxy-3-(6-((4-methoxybenzyl)amino)-9_H_-purin-9-yl)phenyl)-_L_-asparaginate (28).**

$^1$H NMR (400 MHz, DMSO-$d_6$)

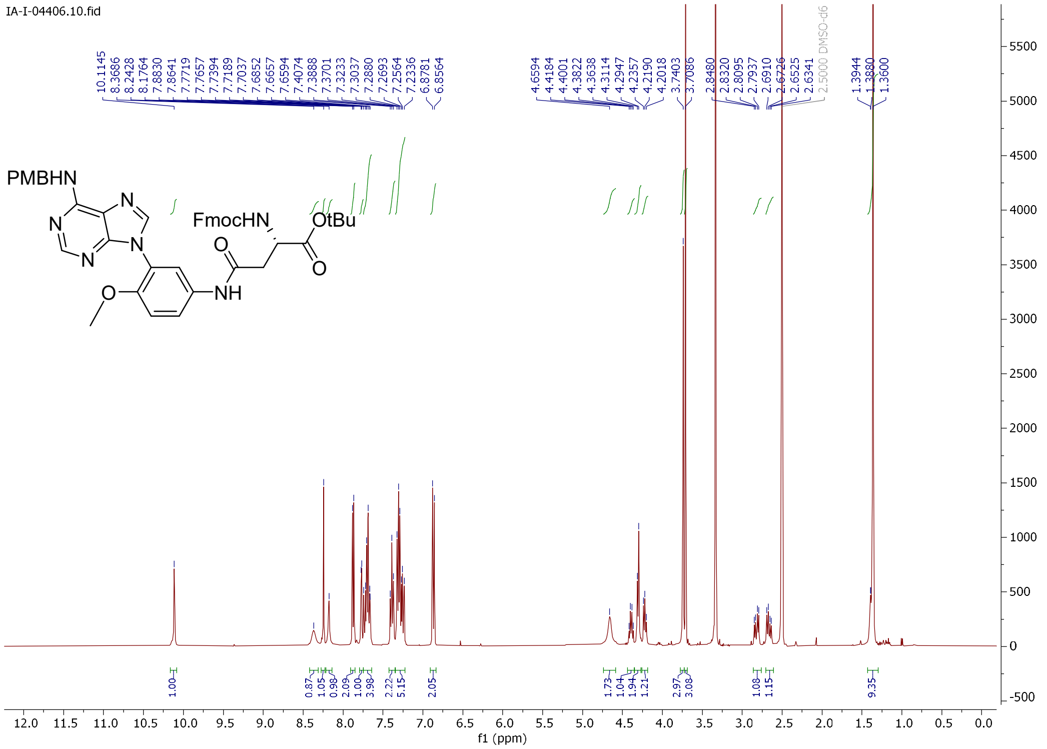


$^{13}$C NMR (100 MHz, DMSO-$d_6$)

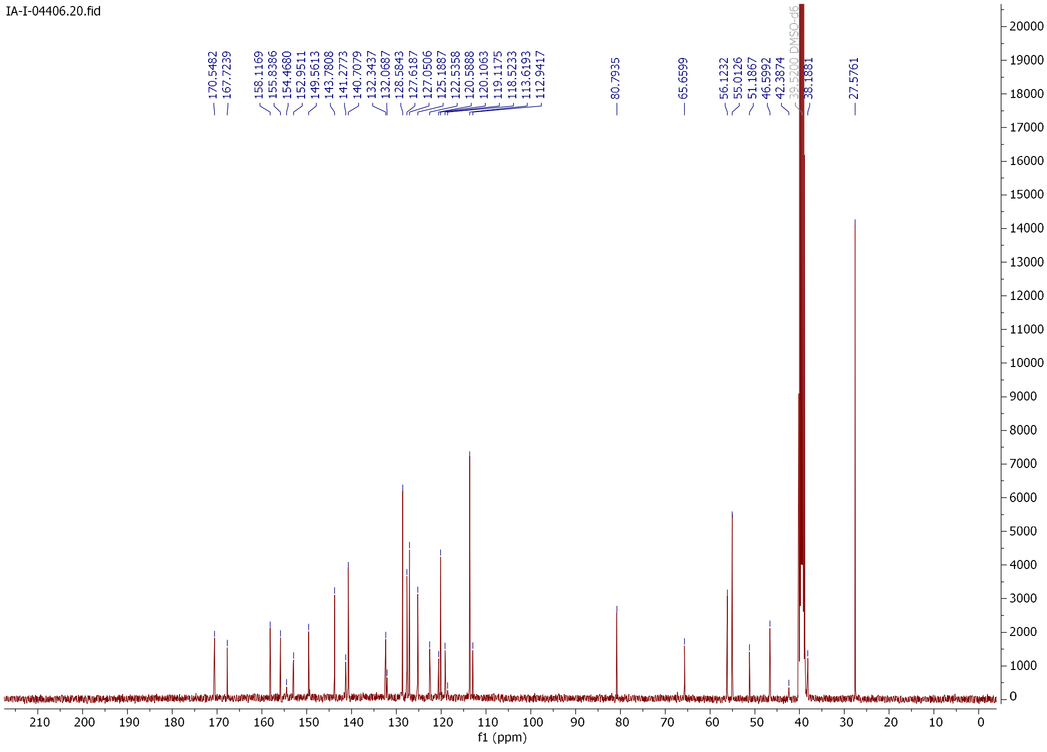

## ***tert*-Butyl *N*[4]-(3-(6-amino-9*H*-purin-9-yl)phenyl)-*L*-asparaginate (29).**

$^{1}$H NMR (400 MHz, DMSO-$d_6$)

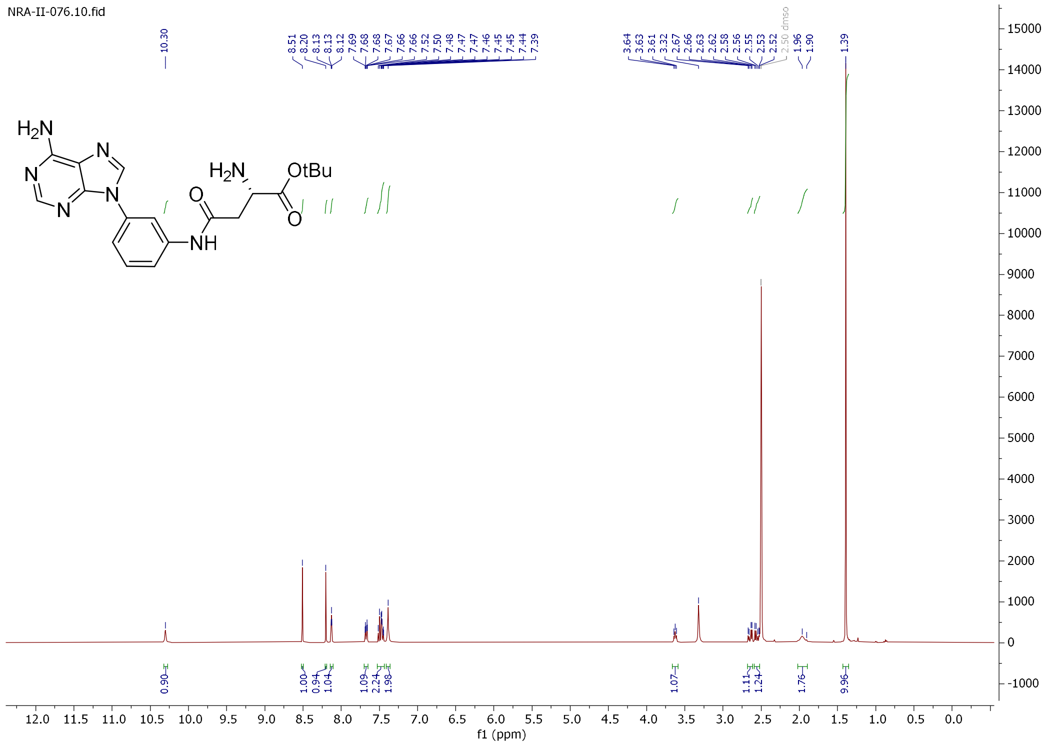


$^{13}$C NMR (100 MHz, DMSO-$d_6$)

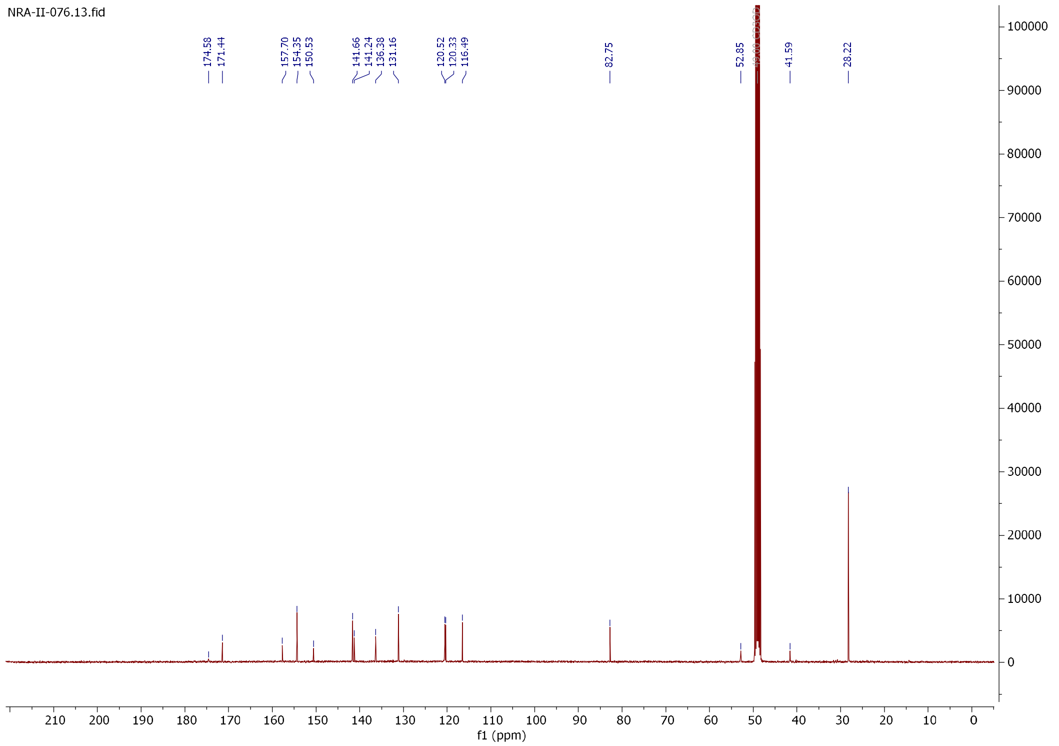

**_tert_-Butyl $N^4$-(3-(6-amino-9$H$-purin-9-yl)-4-methoxyphenyl)-$L$-asparaginate (30).**

$^1$H NMR (400 MHz, DMSO-$d_6$)

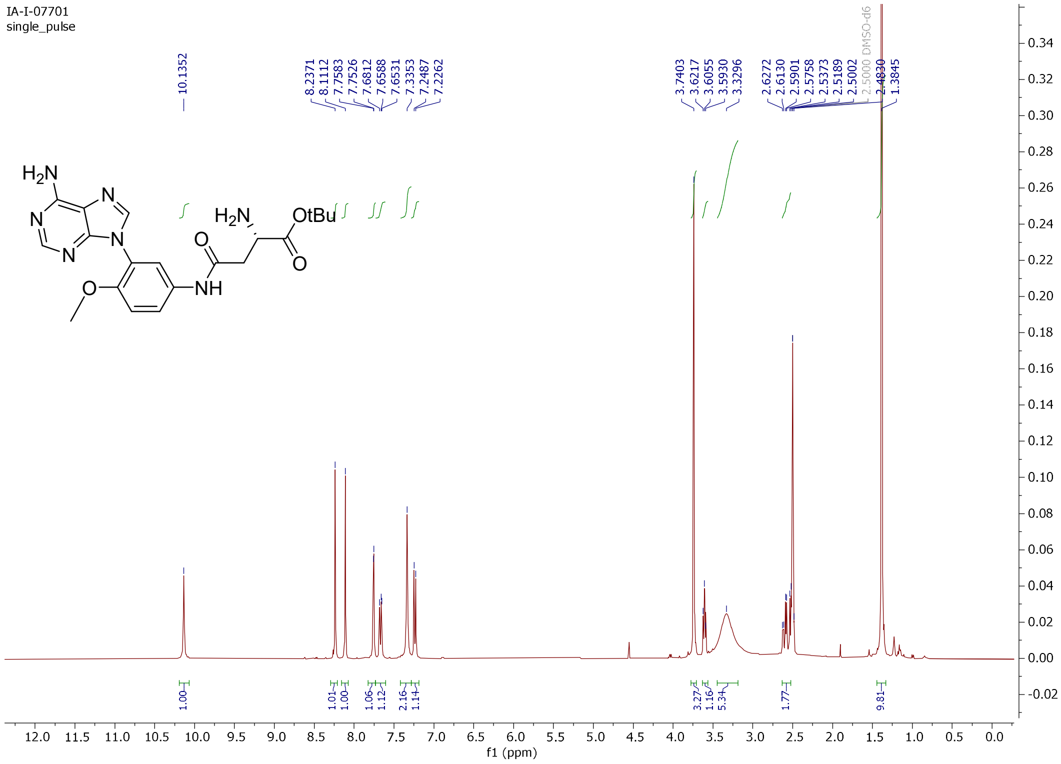


$^{13}$C NMR (100 MHz, DMSO-$d_6$)

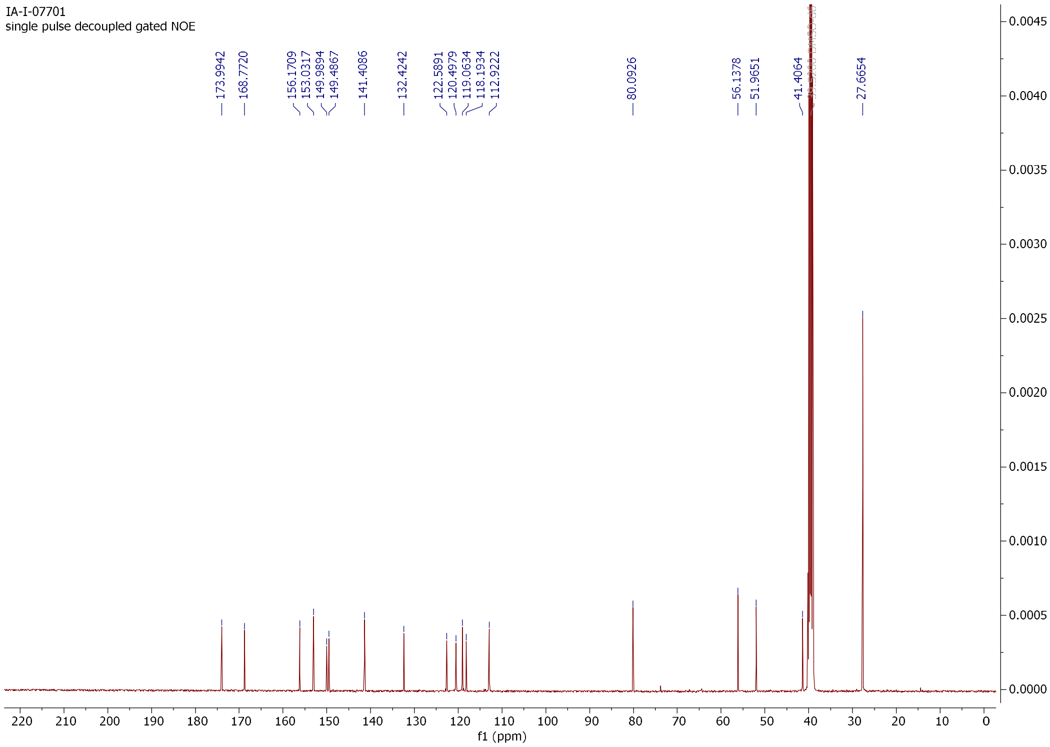

## ***N*⁴-(3-(6-Amino-9*H*-purin-9-yl)phenyl)-*L*-asparagine (31).**

$^{1}$H NMR (400 MHz, DMSO-*d*$_6$)

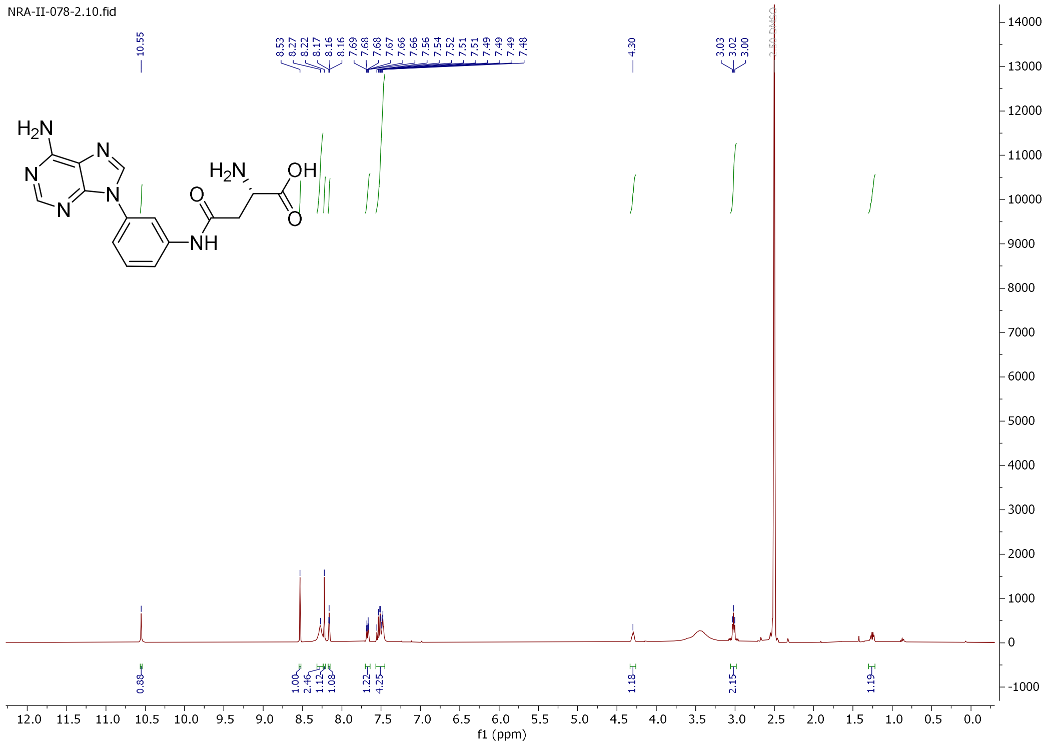


$^{13}$C NMR (100 MHz, DMSO-*d*$_6$)

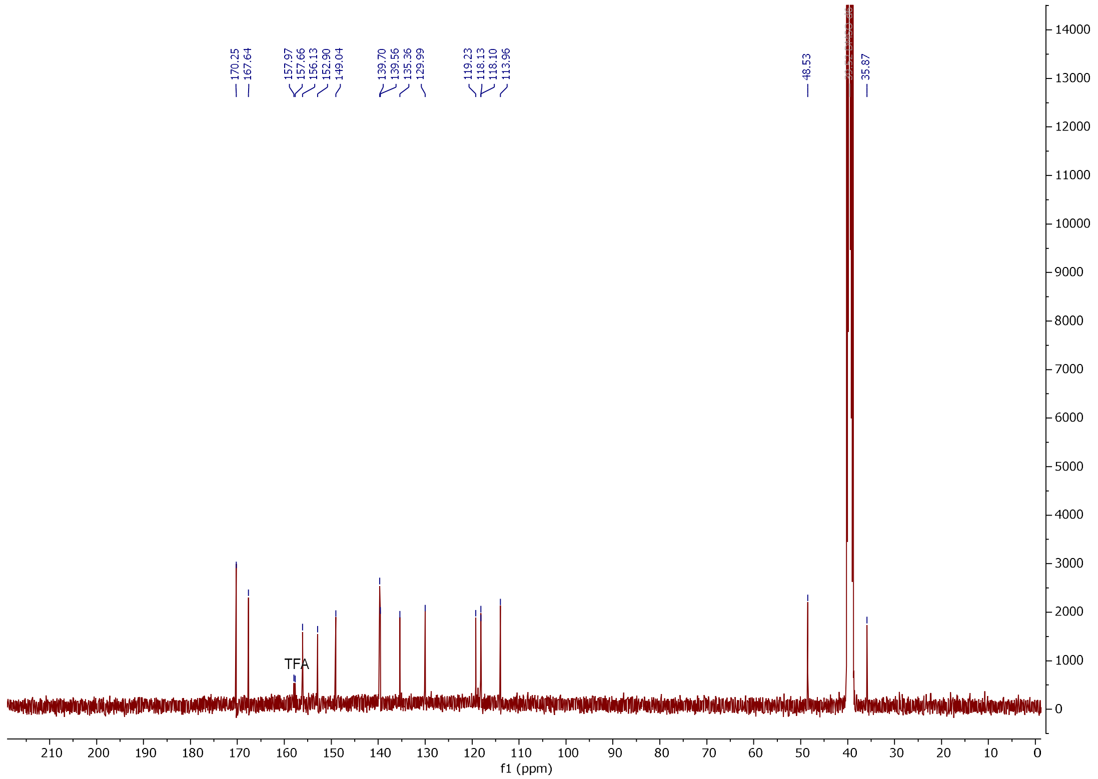

**$N^4$-(3-(6-Amino-9*H*-purin-9-yl)-4-methoxyphenyl)-*L*-asparagine (32).**

$^{1}$H NMR (400 MHz, DMSO-$d_6$)

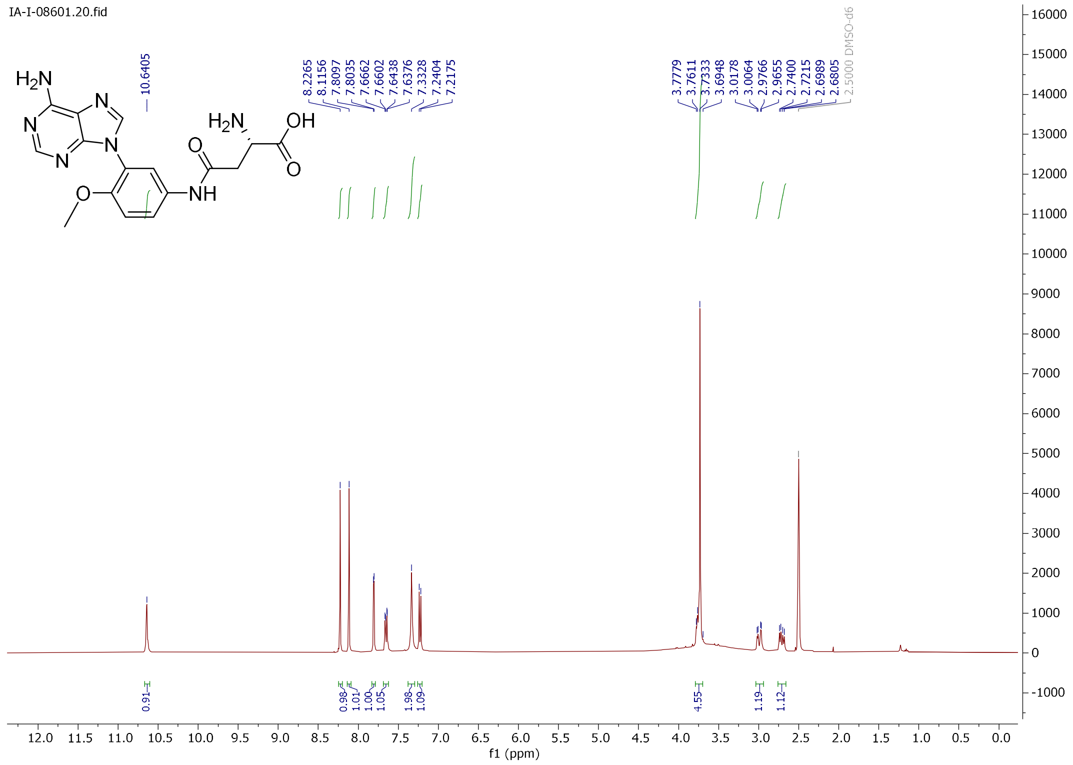


$^{13}$C NMR (100 MHz, DMSO-$d_6$)

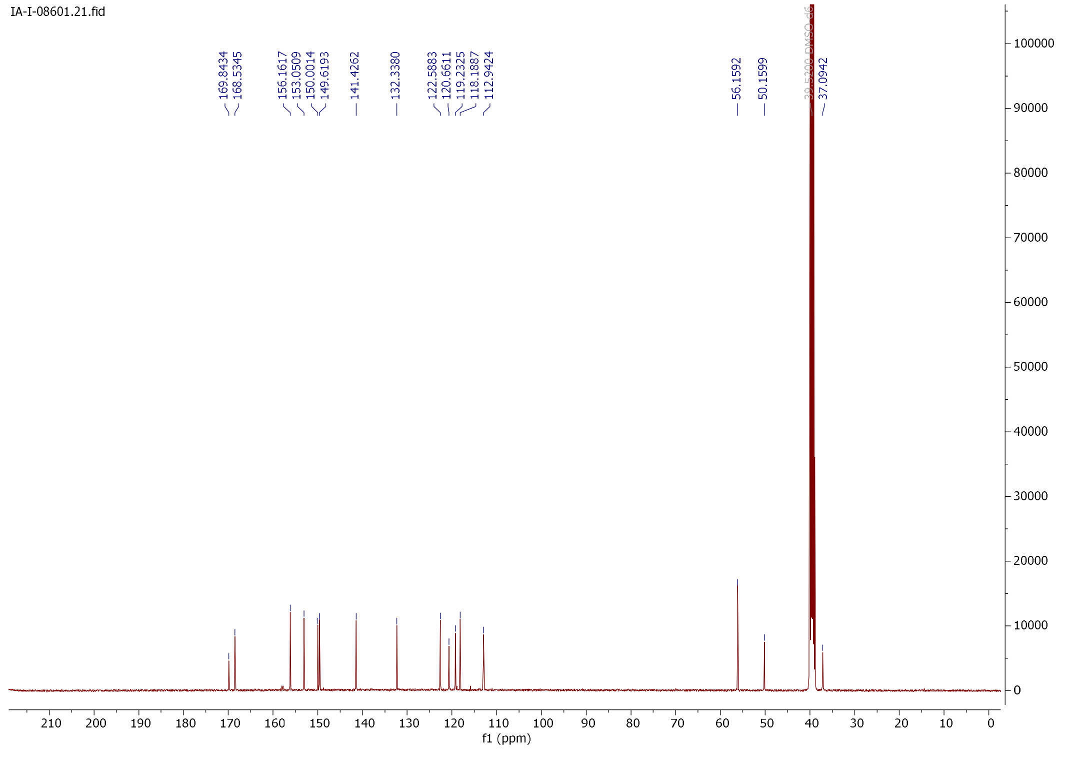

Supplement: RA-015-D5RA05362E-s001 [file RA-015-D5RA05362E-s001.pdf]
